# Ythdc1‐p300‐Klf5 Complex‐Mediated Golgi Dysfunction Promotes Aortic Aneurysm

**DOI:** 10.1002/advs.202512116

**Published:** 2025-11-29

**Authors:** Wen‐Li Wang, Zhi‐Xue Song, Si‐Ming Bu, Yi‐Mei Liu, Qing Li, Wen‐Xia Zhang, Xu‐Rong Sun, Xin Zhang, Yu‐Xuan Xiao, Di Bo, Xu‐Bin Miao, Chao Liu, Xin‐Hua Zhang, Lei Zheng, Hong‐Ye Zhao, Bao‐Lian Zhang, Wen Jia, Kui Chi, Yu Liu, Yong‐Bo Zhao, Bin Zheng, Dong Ma, Jin‐Kun Wen

**Affiliations:** ^1^ Department of Biochemistry and Molecular Biology Key Laboratory of Neural and Vascular Biology Ministry of Education and Hebei Key Laboratory of Cardiovascular Homeostasis and Aging Hebei Medical University Shijiazhuang Hebei 050017 China; ^2^ Department of Obstetrics and Gynecology Vascular Surgery Cardiac Surgery The Second Hospital of Hebei Medical University No. 215 Heping West Road Shijiazhuang 050000 China; ^3^ Cardiac Surgery Department The Fourth Hospital of Hebei Medical University Shijiazhuang Hebei 051000 China

**Keywords:** AA, AD, Golph3l, KLF5, m6A VSMC

## Abstract

Klf5 and Golph3l‐mediated regulation of Golgi morphology has been implicated in the apoptosis of vascular smooth muscle cells (VSMCs), which leads to aortic wall thinning and aneurysm. However, the molecular link between Klf5, Golph3l, and Golgi morphology alteration in the context of the aneurysm is unclear. Here, we show that apoptosis‐induced proliferation (AIP) in VSMCs occurs in aortic dissection (AD) and aortic aneurysm of humans and mice. Golph3l upregulation by Klf5 facilitates TNF‐α and TNFSF12 secretion from AngII‐stimulated VSMCs by inducing Golgi compaction, which is essential for AIP and aneurysm development. Mechanistically, AngII‐induced elevation of global m6A RNA level, especially m6A‐Gm40097, is responsible for Golph3l upregulation and AIP in VSMCs. Further, m6A‐Gm40097 mediates Klf5 interaction with Ythdc1 and p300 to form a transcription complex on the Golph3l promoter, thus activating transcription. Finally, a predictive website for post‐operative short‐term death is built based on AD patient's features, providing a platform to be able to predict risk stratification of AD patients. Collectively, this study identifies a novel lncRNA m6A modification‐dependent regulatory mechanism of chromatin remodeling and transcription activation. Targeting the Ythdc1‐p300‐Klf5 complex may serve as potential therapeutic strategy to improve Golgi dysfunction and aortic aneurysm.

## Graphical abstract

In their Research Article (DOI: https://doi.org/10.1002/advs.202512116* lF: 14.1 Q1), Wenli Wang and co‐workers identifies a novel lncRNA m6A modification‐dependent regulatory mechanism of vascular homeostasis, and constructs a predictive model for post‐operative short‐term death based on AD patient's features, providing a platform to be able to predict risk stratification of AD patients. The cover highlights the Ythdc1‐p300‐Klf5 complex serving as a potential therapeutic strategy to improve Golgi dysfunction and aortic aneurysm.

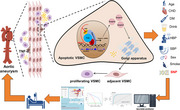

## 1 Introduction

VSMCs)are the major cell type within the aortic wall and play a crucial role in regulating vessel tone and blood pressure as well as in maintaining vascular homeostasis. In response to vascular injury or pathological stimuli, such as angiotensin II (AngII), oxidative stress, and inflammatory factors, the abnormal proliferation, migration, and apoptosis of VSMCs, a common event in many vascular diseases, lead to arterial remodeling. Despite the accumulating evidence showing that pathophysiological mechanisms responsible for vascular remodeling involve the loss of VSMCs, extracellular matrix (ECM) degradation, and inflammatory cell infiltration, all of which contributes to weakening of the aortic wall^[^ ^1^, ^2^, ^3^ ^]^ the loss of VSMCs caused by VSMC apoptosis in the medial layer of the aortic wall is a key mechanism leading to decreased regeneration of ECM, weakened aortic wall, and dilated blood vessel, ultimately resulting in the development of aortic aneurysms and dissections.^[^ ^4^, ^5^ ^]^ The density/number of VSMCs within the arterial wall is determined by the balance between VSMC proliferation and apoptosis. To maintain tissue homeostasis after pathological stimulus‐induced apoptosis, apoptotic cells are able to stimulate neighboring surviving cells to undergo proliferation to compensate for the tissue loss, a phenomenon termed (AIP).^[^ ^6^, ^7^ ^]^ In this regard, a recent study demonstrated that VSMC apoptosis can also induce apoptosis or proliferation in adjacent live VSMCs by releasing cytokines involved in the regulation of cell proliferation and apoptosis.^[^ ^8^ ^]^ However, the precise mechanism underlying AIP in VSMCs during aortic remodeling remains to be clarified.

Krüppel‐like zinc‐finger transcription factor Klf5 regulates various cellular processes, including proliferation, differentiation, and apoptosis, which play indispensable roles in the development of vascular remodeling.^[^ ^9^ ^]^ Thus, Klf5 has been identified as a significant factor associated with various types of cardiovascular complications during the last two decades.^[^ ^10^ ^]^ In VSMCs, Klf5 is regulated by AngII signaling and is an essential regulator of cardiovascular remodeling.^[^ ^11^ ^]^ Our recent studies show that Klf5 is highly upregulated in VSMCs of human and experimental mouse abdominal aortic aneurysm (AAA); Klf5 knockdown exacerbates, while its overexpression suppresses AngII‐induced AAA formation.^[^ ^12^, ^13^ ^]^ Although Klf5 is known to promote VSMC proliferation by activating cell cycle genes and to prevent VSMC apoptosis by regulating apoptosis‐related proteins, including Bax, Bcl‐2, surviving, and caspase‐3/9,^[^ ^14^, ^15^ ^]^ it has not been investigated whether and how Klf5 regulates AIP in VSMCs during aortic remodeling.

The Golgi apparatus is a membrane‐bound organelle, and it plays a crucial role in regulating post‐translational protein modification as well as in sorting out proteins and other biomolecules to the cell surface and to the extracellular milieu.^[^ ^16^ ^]^ In addition to these functions, the Golgi also serves as a signaling hub to participate in the regulation of calcium and pH homeostasis, stress response, apoptosis, and autophagy.^[^ ^17^, ^18^ ^]^ The Golgi undergoes significant changes in structure and function under various stress conditions, such as DNA damage, oxidative stress, aging, and proapoptotic stimulation, etc.^[^ ^19^ ^]^ For example, in the process of apoptosis, the Golgi undergoes irreversible fragmentation by caspase2/3‐mediated cleavage of some Golgi‐resident proteins, leading to the dispersal of the Golgi.^[^ ^19^ ^]^ Morphological changes in the Golgi during apoptosis include swelling, loss of its ribbon‐like structure, and the failure to maintain the perinuclear distribution.^[^ ^20^ ^]^ Previous studies have demonstrated that Golgi morphology and function are regulated by the Golph3/Golph3l pathway.^[^ ^21^, ^22^ ^]^ Although Golph3 and Golph3l are both required for efficient secretion, they exert opposing roles in maintaining Golgi morphology. Overexpression of Golph3l results in Golgi compaction, whereas its depletion leads to Golgi dispersal and impairs Golgi‐to‐plasma membrane secretory trafficking. Conversely, overexpression of Golph3 results in a more dispersed Golgi.^[^ ^21^, ^22^ ^]^ Despite the existing evidence showing that the Golph3/Golph3l pathway is activated in response to DNA damage and plays a role in the pathophysiology of neurodegenerative disease, the molecular link between Klf5, Golph3l, and Golgi morphology alteration in the context of the aortic remodeling has not been investigated.

In this study, we found that AIP in VSMCs occurs in the aortic tissues of mouse AD and AAA models as well as in human clinical samples. Therefore, we utilized an AngII‐induced AIP experiment to investigate how AngII‐induced apoptotic VSMCs exert the stimulatory effect on adjacent VSMC proliferation. We show that upregulation of Golph3l by Klf5 facilitates TNF‐α and TNFSF12 secretion from AngII‐stimulated VSMCs by modulating the Golgi morphology, which is essential for AIP in VSMCs and aortic aneurysm formation. Furthermore, we identified that Klf5‐mediated transcriptional activation of the Golph3l promoter involves the synergistic effect of epigenetic regulation and chromatin remodeling by recruiting Ythdc1, p300, and Klf5 to the Golph3l promoter in an m6A‐Gm40097‐dependent manner. Eventually, we demonstrated that SNP in the upstream regulatory region of Golph3l gene is associated with the development of AD and AAA. Based on our findings combined with routinely available clinical data, a machine learning‐based website for the prediction of the postoperative short‐term death of AD patients was developed.

## 2 Results

### 2.1 AIP Occurs in VSMCs of AD and AAA Tissues

**Figure 1:**
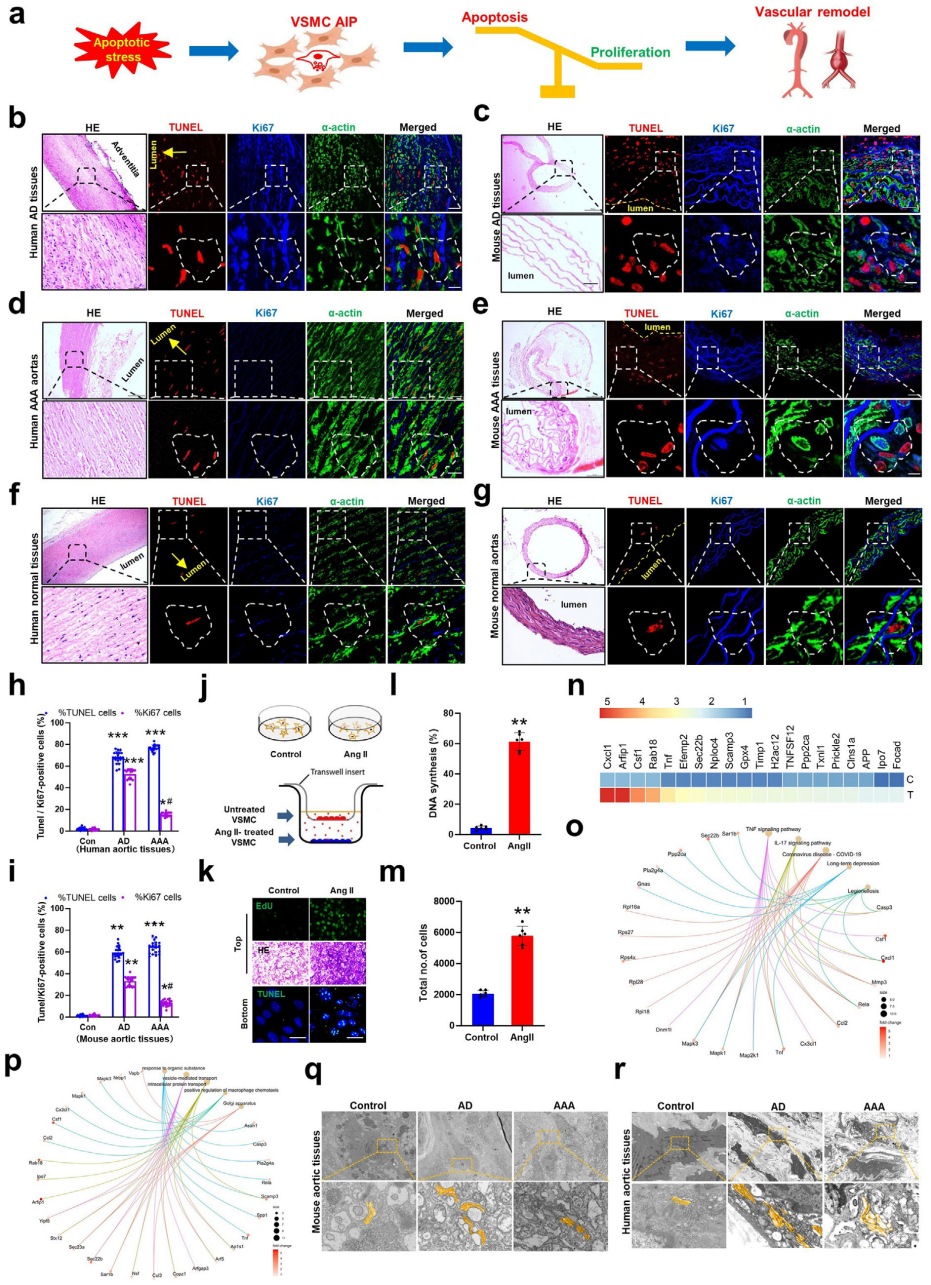
Apoptosis‐induced proliferation (AIP) in VSMCs is associated with vascular remodeling. a) Schematic showing the AIP of VSMCs impaired vascular homeostasis. b–g) Representative photographs of hematoxylin and eosin (HE) staining and immunostaining with VSMC marker (α‐actin, SMAα), terminal deoxynucleotidyl transferase‐mediated deoxyuridine triphosphate nick end labeling (TUNEL), and Ki‐67 in human aortic dissection (AD) (*n* = 21), abdominal aortic aneurysm (AAA) (*n* = 13), and normal aortas (*n* = 8) as well as in mouse AD, AAA, and normal descending thoracic aortic sections (each group *n* = 5). Scale bars = 50 µm. h,i) Statistics of TUNEL+ SMAα‐positive cells and Ki67+ SMAα‐positive cells in human h) and mouse i) AD, AAA tissues and control, respectively. *p < 0.05, **p < 0.01, and ***p < 0.001 versus control or ^#^ *p* < 0.05 versus AD. j) An in vitro coculture system is shown where VSMCs were seeded in the top compartment and angiotensin II (AngII)‐stimulated VSMCs in the bottom compartment. k) TUNEL assay showing apoptotic VSMCs induced by AngII in the bottom compartment. Scale bars = 20 µm. The nucleus was counterstained with 4′,6‐diamidino‐2‐phenylindole (DAPI). Middle: representative photographs showing an increased number of VSMCs stained by hematoxylin and eosin (HE) in the top compartment. Top: fluorescence image of 5‐ethynyl‐2′‐deoxyuridine (EdU) incorporation assay showing proliferative activity of VSMCs in the top compartment. l,m) Statistic of the percentage of DNA synthesis l) and the total number m) of mouse VSMCs in top compartment. *n* = 3 for each group. **p < 0.01 versus control. n) Heatmap of IP‐MS analysis showing top 20 proteins enriched in cultured medium from VSMCs stimulated with or without AngII. o) Kyoto encyclopedia of genes and genomes (KEGG) pathway analysis of the secreted proteins showing a significant up‐regulation of TNF‐α signaling pathway‐related protein expression. p) Gene ontology (GO) analysis showing a significant up‐regulation of Golgi apparatus‐related proteins. q,r) Transmission electron microscopy reveals Golgi morphology in mouse q) and human r) AD and AAA tissues as well as in their corresponding controls.

Because AIP occurs widely in normal development and tissue homeostasis, and apoptosis of VSMCs contributes to multiple vascular pathologies,^[^ ^23^ ^]^ we first sought to demonstrate the association between AIP in VSMCs and vascular remodeling‐related diseases, such as aortic dissections and aneurysms. According to the hypothesis that the balance between apoptosis and proliferation of VSMCs was destroyed in aortic diseases (**Figure** **1**a), we determined the AIP alternation in human thoracic AD and AAA tissues (Participants' basic information is shown in Table S1, Supporting Information) and in mouse models of AD and AAA by immunofluorescence staining of VSMC marker (smooth muscle α‐actin, SMA), Ki‐67, and TUNEL. The results showed that AIP was observed in the histological sections from mouse AD and AAA (Figure 1b,e) and clinical samples (Figure 1c,d) as well as corresponding control (Figure 1f,g). Importantly, the percentages of TUNEL‐positive and adjacent Ki‐67‐positive VSMCs were significantly increased in two diseased arteries of mice (Figure 1i) and humans (Figure 1h) than in the normal aortas (AD, TUNEL‐positive: 59.1% ± 6.7% vs 2.4% ± 0.89%, Ki‐67‐positive: 36.6% ± 1.9% vs 2.9% ± 0.72%; AAA, TUNEL‐positive: 66.7% ± 5.4% vs 2.4% ± 0.89%, Ki67‐positive: 15.1% ± 1.6% vs 2.9% ± 0.72%). Remarkably, the number of Ki‐67‐positive VSMCs was higher in AD tissues than that in AAA.

The above results indicate the existence of an excessive AIP during pathological vascular remodeling. It is well known that AngII promotes pathological vascular remodeling by inducing VSMC proliferation, migration, hypertrophy, and apoptosis.^[^ ^24^, ^25^ ^]^ We next performed an AngII‐induced AIP experiment in mouse VSMCs by coculturing AngII‐treated/untreated VSMCs in a transwell chamber culture system, where AngII‐untreated VSMCs were seeded in the upper chamber and AngII‐stimulated VSMCs in the lower chamber (Figure 1j). Compared with control group, treating VSMCs (bottom compartment) with AngII markedly induced cell apoptosis, as evidenced by TUNEL staining, which was accompanied by increased proliferation of VSMCs grown in the top compartment, as shown by hematoxylin andHE staining and EdU incorporation assays (Figure 1k–m). These suggest that AngII‐induced apoptotic VSMCs are able to stimulate adjacent VSMC proliferation. To identify and characterize which protein(s) secreted by AngII‐induced apoptotic VSMCs induces the proliferation of adjacent VSMCs, we used mass spectrometry‐based secretome analysis to determine the differentially expressed proteins in serum‐free medium between the AngII‐treated VSMCs and AngII‐untreated VSMCs. As shown in Table S2 (Supporting Information), 69 differentially expressed proteins (>1.5 fold) were identified from AngII‐treated versus untreated VSMCs, and top 20 significantly upregulated proteins were shown in Figure 1n. KEGGpathway analysis revealed that the functional terms of the differentially expressed proteins were related to the TNF‐α signaling, IL‐17 signaling, and cellular secretion pathway (Figure 1o). To confirm whether TNF‐α exerts a key role in driving AIP of VSMCs, we used anti‐TNF‐α antibody Golimumab to neutralize TNF‐α and then tested the effectiveness of Ang II‐stimulated AIP in the VSMC coculture system. Indeed, compared with control group, AngII‐induced proliferation of adjacent cells was largely prevented in the presence of Golimumab (Figure S1, Supporting Information), suggesting that neutralizing TNF‐α attenuates AIP. This implies that Ang II‐stimulated VSMCs promote proliferation of neighboring cells by releasing TNF‐α.

The GO analysis revealed that treating VSMCs with AngII significantly upregulated the expression of genes highly enriched in terms related to the Golgi apparatus (GA) (Figure 1p). The Golgi complex plays a crucial role in protein secretion by regulating cargo sorting and trafficking.^[^ ^26^ ^]^ Considering that GA is one of the organelles to be affected during apoptosis, and several Golgi matrix proteins relevant to Golgi structure maintenance are cleaved by caspases during apoptosis, leading to Golgi fragmentation,^[^ ^27^, ^28^ ^]^ we checked the Golgi morphology changes in VSMCs of AD and AAA tissues from mouse models and clinical samples using transmission electron microscopy. Unbiased screening led to the discovery that a significantly compacted Golgi was presented in VSMCs of the two diseased aortic tissues from mice (Figure 1q) and humans (Figure 1r), whereas a dispersed Golgi pattern was displayed prominently in control artery tissues.

Additionally, to establish a causal relationship between apoptosis and proliferation, activation of AIP was also detected in VSMCs during staurosporin‐induced apoptosis, a commonly used way of provoking programmed cell death (Figure S2a, Supporting Information). As expected, staurosporin yielded the same effect as AngII in inducing AIP of VSMCs. In line with this, Golgi dynamic change and TNF‐α level revealed a trend consistent with that of AIP activation (Figure S2b,c, Supporting Information). This suggests that staurosporin‐induced apoptosis can also enhance the proliferation of neighboring VSMCs. Furthermore, to determine the link between apoptosis and proliferation, AngII‐induced AIP at different stages of mouse AD was examined (Figure S3, Supporting Information). Our results showed that, in the early stage (day 7) of Ang II‐induced aneurysm‐related AIP, apoptotic VSMCs predominately induced the proliferation of neighboring VSMCs, whereas in the middle (day 14) and late (day 21) stages, apoptotic VSMCs mainly promote the adjacent VSMC apoptosis, indicating an unbalance between apoptosis and proliferation of VSMCs, which in turn leads to the loss of VSMCs and aneurysm. Collectively, these results imply a causal relationship between VSMC AIP and pathological vascular remodeling, and suggest that dysregulated protein secretions elicited by Golgi structure alteration might be responsible for AIP in VSMCs.

### 2.2 Increased m6A RNA Methylation is Correlated with AIP in VSMCs During the Development of AD and AAA

**Figure 2:**
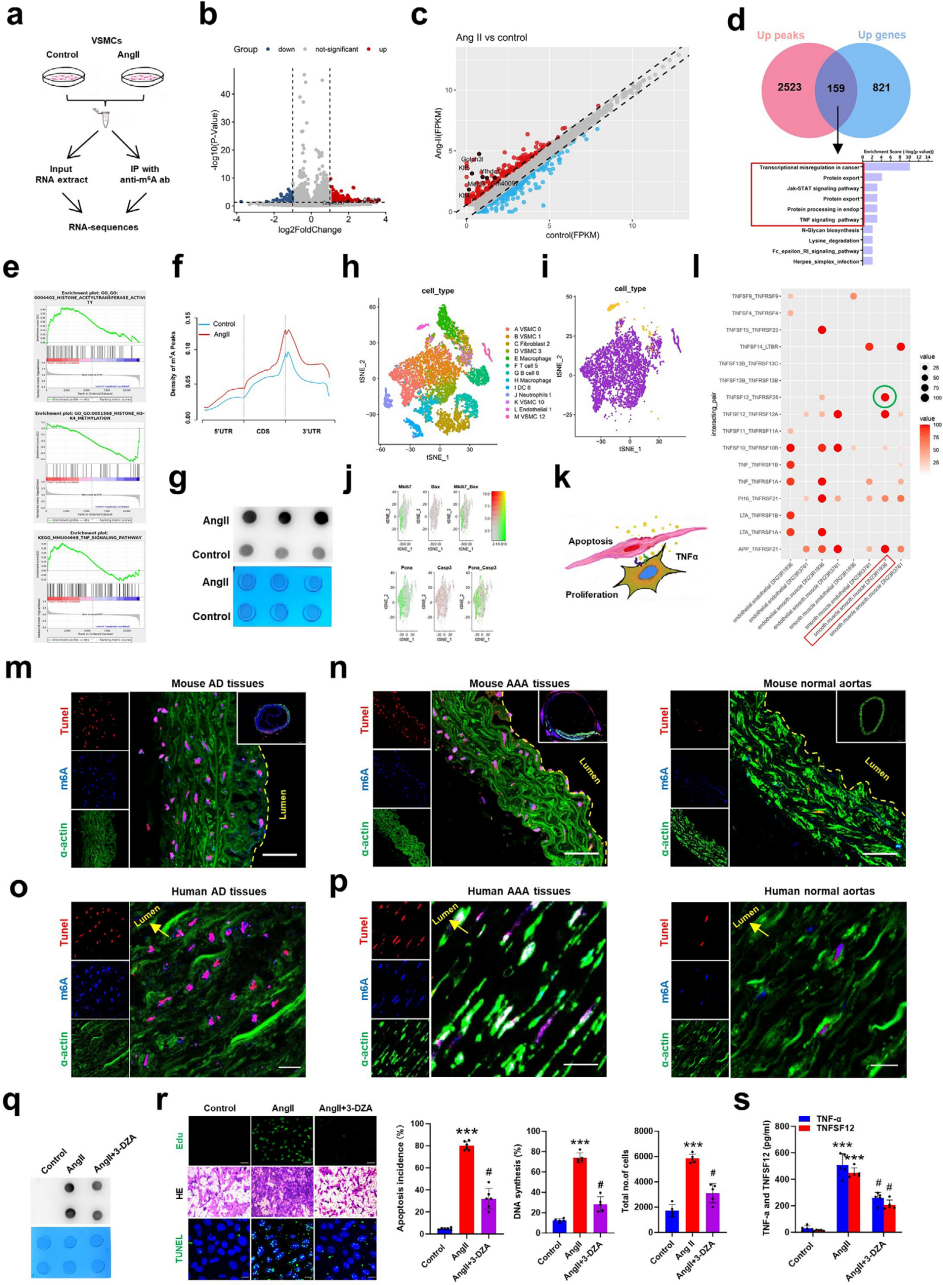
Increased m6A RNA methylation is correlated with AIP in VSMCs. a) Illustration of the combined m6A MeRIP‐seq and RNA‐seq experimental design in mouse VSMCs treated with and without AngII (10^−7^ mol L^−1^) for 24 h. b) Volcano plot of MeRIP‐seq peaks showing the differential expressed m6A levels in AngII‐induced apoptotic VSMCs and untreated VSMCs (control). The blue dots and red dots represent hyper‐methylated and hypo‐methylated peaks, respectively. c) Scatterplot of the differential expressed genes (DEGs) with blue and red dots represent the downregulated and upregulated DEGs, respectively. d) Venn diagram and KEGG pathway maps visualizing the 159 overlapping genes between the upregulated m6A peaks and upregulated mRNAs in mouse VSMCs treated or not with AngII. e) GSEA for interpreting the differential expressed genes and m6A modification profiles between AngII‐treated cells and control groups. f) Distribution of m6A peaks throughout the mRNA loci from 5′UTR to 3′UTR. Blue line and red line represent the total peaks in control and AngII‐treated group, respectively. g) Dot blot assay using anti‐m6A antibodies revealed a higher global m6A level in AngII‐treated group than that in control. h) tSNE clustering plots showing all color‐coded cell clusters in BAPN/AngII‐injured mouse AD tissues. i) tSNE plot showing all VSMCs (violet dotted line circles) and endothelial cells (yellow dotted line circles) in AngII‐induced mouse AD tissue colored by clusters. j) Relative expression of apoptotic marker (Bax and Caspase3) and proliferative marker (PCNA and Ki‐67) in VSMC clusters projected onto a tSNE plot. k) Schematic illustration of the proposed model: the pro‐proliferative effects of apoptotic VSMCs on adjacent VSMCs through releasing signaling molecules. l) CellPhone DB analysis for ligand‐receptor pairs expressed by apoptotic VSMCs. m–p) Confocal immunofluorescence staining performed on mouse m,n) and human o,p) AD and AAA sections as well as their corresponding control sections stained with SMA‐α (green), m6A (blue) antibodies, and TUNEL (red). Scale bars = 50 µm. q) Mouse VSMCs were treated with vehicle and 3‐DZA for 24 h, and then stimulated with Ang II. Global m6A level of RNA was detected by dot blot assay using anti‐m6A antibodies. r) Representative photographs of EdU incorporation assay (Top), HE staining (Middle), and TUNEL assay (Down) for detecting VSMC proliferation and apoptosis, respectively. Percentage of TUNEL‐positive cells, EdU‐positive cells and total number of cells are shown as histograms. s) Enzyme‐linked immunosorbent assay (ELISA) showing the levels of TNF‐α and TNFSF12 in cultured medium from VSMCs with AngII or AngII+3‐DZA treatment. The data represent mean ± SEM of 5 independent experiments. ***p < 0.001 versus control, ^#^ *p* < 0.05 versus AngII group.

N6‐methyladenosine (m6A) RNA modification is the most abundant and prevalent epigenetic modification of mRNA and has been reported to promote the occurrence and development of cardiovascular diseases, including pulmonary arterial hypertension,^[^ ^29^ ^]^ cardiac remodeling, heart failure, and atherosclerosis.^[^ ^30^ ^]^ To define whether m6A modification participates in AIP in VSMCs during vascular remodeling, we performed an integrated analysis of m6A antibody‐based methylated RNA immunoprecipitation sequence (MeRIP‐seq) and RNA‐sequencing (RNA‐seq) for input in mouse VSMCs subjected to AngII treatment (**Figure** **2**a). The resulting volcano plots and scatterplots revealed markedly up‐regulated m6A peaks (*n* = 2681 peaks, Figure 2b) and increased mRNA expression (*n* = 980, Figure 2c) in AngII‐treated VSMCs compared to control. Furthermore, transcriptome‐wide analysis of RNA m6A modification revealed a significant increase in m6A peaks in AngII‐treated VSMCs at mRNA containing loci (Figure 2d). Notably, we observed an overlap between up‐regulated m6A peaks and increased mRNAs in 159 out of the 980 actively expressed genes (Figure 2d). These overlapped genes were mainly enriched in signal pathways correlated with transcriptional misregulation, protein export, TNF‐α signaling, protein processing, and so on (Figure 2d). Consistently, gene set enrichment analysis (GSEA) showed that the up‐regulated genes with increased m6A in AngII‐stimulated VSMCs not only involved TNF‐α signaling pathway, but also included histone acetyltransferase and histone H3K4 methyltransferase, both of which play an important role in the regulation of histone modifications and transcription (Figure 2e). Additionally, the results of dot blot assay using anti‐m6A antibody further confirmed that the increased m6A methylated RNAs were consistent with the sequencing results of the whole mRNA sequence (Figure 2f,g).

To further delineate the crosstalk between cellular components in diseased aortas, we performed the single‐cell RNA sequencing (scRNA‐seq) of aortic tissues of mice that were infused with saline or BAPN+AngII for 21 days. Among the 12915 qualified cells from all samples, we identified 13 clusters that were assigned to 10 cell lineages through clustering of the datasets visualized by the *t‐*distributed stochastic neighbor embedding (*t*‐SNE) plot as well as according to the expression of cell type‐specific marker genes (Figure 2h). To gain an overall insight into VSMC AIP during aortic remodeling, we performed interactively unsupervised clustering and classified the entire VSMC population into two clusters or two phenotypes, i.e., apoptosis and proliferation, based on their respective “‘specific”’ markers: Bax (Bcl‐2‐associated X protein) and Ki‐67 or Caspase 3 and PCNA (Figure 2i,j). The results of immunofluorescent staining using anti‐Bax antibody further demonstrated the AIP happened in aortic tissues from mouse AD model (Figure S4, Supporting Information). Inference of cell‐cell communication from scRNA‐seq data showed apparently increased interactions of receptor–ligand pairs associated with TNF‐α signaling between apoptotic and proliferative VSMCs, especially TNFSF12‐TNFRSF25 (Figure 2k,l). This is consistent with the view that TNF‐α released from apoptotic cells can enhance the proliferation of neighboring cells in a secretome‐dependent manner. Further, we determined the m6A methylation in the apoptotic VSMCs of AD and AAA tissues from mouse models and clinical samples using confocal fluorescence microscopy by staining with anti‐SMA (VSMC marker), anti‐m6A, and TUNEL staining. As expected, the increased m6A methylation was detected in the VSMCs of the two diseased aortic tissues from mouse models (Figure 2m,n) and clinical samples (Figure 2o,p) compared with the corresponding control. We next examined whether m6A RNA modification is essential for AngII‐induced AIP in VSMCs and found that inhibition of m6A methylation by treating AngII‐stimulated VSMCs with 3‐deaza‐adenosine (3‐DZA, a methylation inhibitor), as verified by dot blot assay (Figure 2q), significantly attenuated AngII‐induced VSMC apoptosis, which was simultaneously accompanied by a decreased proliferation of VSMCs treated without AngII (Figure 2r). Importantly, TNF‐α and TNFSF12 expression was substantially upregulated in AngII‐stimulated VSMCs, whereas inhibition of m6A methylation by 3‐DZA treatment was able to abrogate their upregulation (Figure 2s). All together, these findings suggest that increased RNA m6A modification is correlated with AIP in VSMCs and that the VSMCs undergoing apoptosis can release TNF‐α to promote proliferation of neighboring cells during the development of aortic remodeling.

### 2.3 Golph3l is Upregulated in AngII‐Stimulated VSMCs and is Essential for AIP and Formation of AD and AAA

**Figure 3:**
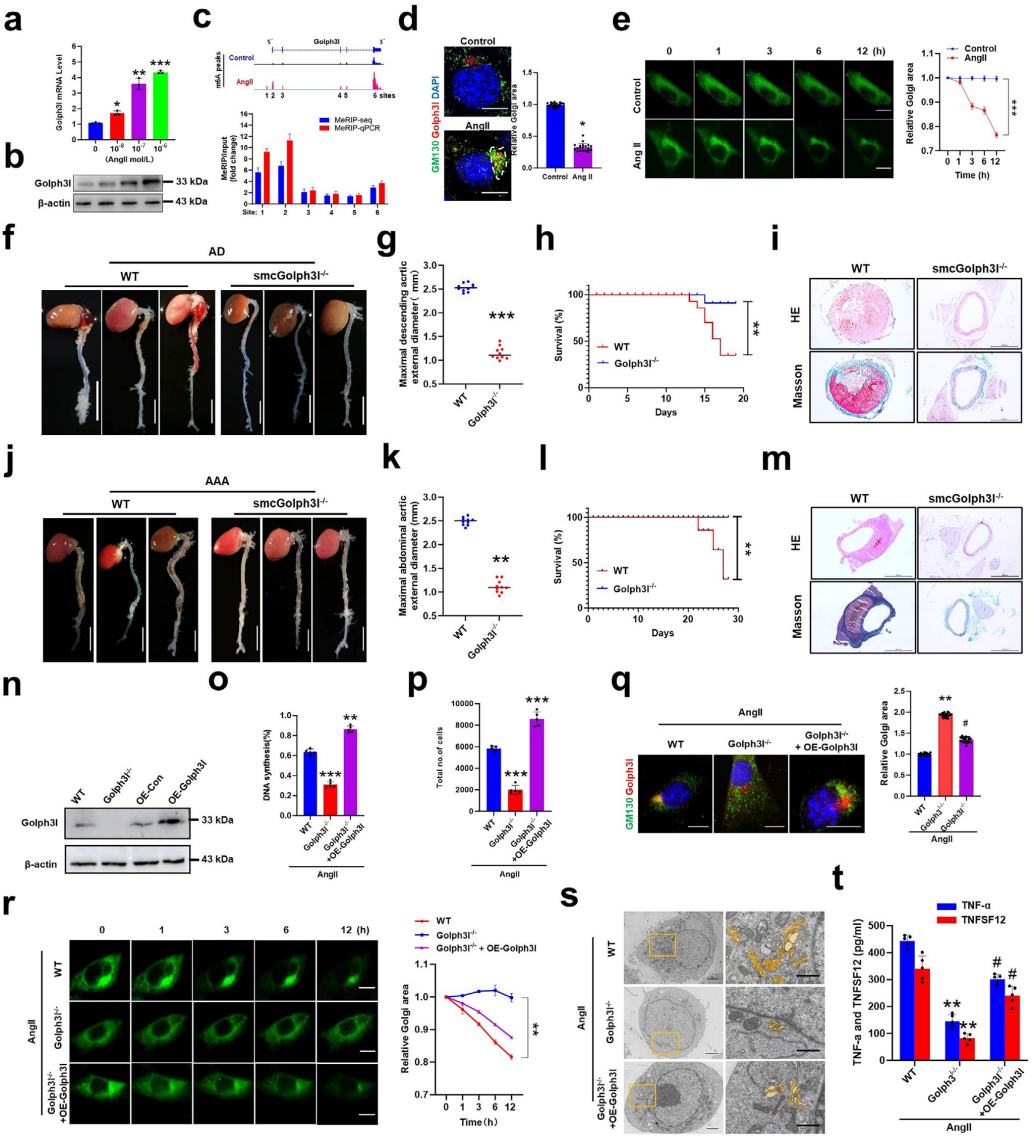
Golph3l is upregulated in AngII‐stimulated VSMCs and is essential for AIP. a,b) Mouse VSMCs were stimulated with different concentrations of AngII (0, 10^−8^, 10^−7^, 10^−6 ^mol L^−1^), and then Golph3l expression was detected by qRT‐PCR a) and western blotting b), respectively. *N* = 3 for each group. c) The visualization of m6A abundances in Golph3l mRNA transcripts by Integrative Genomics Viewer (IGV) in VSMCs treated or not with AngII. Down: Methylated RNA immunoprecipitation (MeRIP)‐qPCR confirmation of distinct m6A modification sites obtained from MeRIP‐seq results. d) Immunofluorescence staining of cis‐Golgi protein GM130 (green), Golph3l (red), and DAPI staining (blue). Scale bars = 10 µm. Right: Statistical analysis of Golgi area ratio in AngII‐treated cells relative to control. e) The Golgi apparatus was stained with Golgi‐Tracker green and traced using time‐lapse imaging in AngII‐stimulated VSMCs. Right: Quantitative analysis of Golgi‐Tracker green area in live cells treated with or without AngII, along with Golgi area being measured (pooled from three independent experiments) and *p* values being indicated (by unpaired *t‐*test). f) Representative photographs of mouse aortas from β‐aminopropionitrile (BAPN) and AngII‐induced AD in WT and smcGolph3l^−/−^ mice. g,h) Statistical analysis of the maximal aortic external diameter g) and the survival rate by log‐rank test h). i) HE‐stained (top row) and Masson‐stained (bottom row) aortic sections. j) Representative photographs of mouse aortas from AngII‐induced AAA in ApoE^−/−^ and ApoE^−/−^+ smcGolph3l^−/−^ mice. Scale bars = 2.5 mm. k,l) Statistical analysis of the maximal aortic external diameter k) and the survival rate by log‐rank test l). Data represent the mean ± SD. **p < 0.01, ***p < 0.001 versus WT mice. *n* = 10 for each group. m) HE‐stained (top row) and Masson‐stained (bottom row) aortic sections. Scale bars = 50 µm. n) Primary VSMCs of smcGolph3l^−/−^ mice were transfected with pcDNA3.1 (OE‐Con) or pcDNA3.1‐Golph3l (oe‐Golph3l) for 24 h, and then Golph3l expression was detected by western blotting. o,p) Percentage of EdU‐positive cells o) and total number of cells p) are shown as histograms. The data represent mean ± SEM of 5 independent experiments. **p < 0.01, ***p < 0.001 versus WT. q) Immunofluorescence staining of GM130, Golph3l, and nuclei (DAPI) was performed to visualize Golgi morphology. Right: Statistical analysis of Golgi‐Tracker green area relative to WT. Data represent mean ± SEM of 3 independent experiments in which 50 cells were analyzed. **p < 0.01 versus WT, ^#^ *p* < 0.05 versus Golph3l^−/−^. r) A time‐lapse image showing the Golgi dynamics in live mouse VSMCs treated as described in (n). Right: Quantitative analysis of Golgi‐Tracker green Golgi‐Tracker green area. **p < 0.01 versus WT. s) Golgi morphology was observed by transmission electron microscopy in WT, Golph3l^−/−^, and Golph3l^−/−^ +OE‐Golph3l‐transfected VSMCs. t) ELISA showing the levels of TNF‐α and TNFSF12 in cultured medium of VSMCs treated as described in (n). The data represent mean ± SEM of 5 independent experiments. **p < 0.01 versus WT, ^#^ *p* < 0.05 versus Golph3l^−/−^.

To explore the molecular mechanism regarding how AIP in VSMCs is regulated in the aortic remodeling, we further analyzed and compared MeRIP‐seq and RNA‐seq data from AngII‐treated versus untreated VSMCs. We found that an overlapping gene Golph3l between MeRIP‐seq and RNA‐seq data was ranked on the top based on fold of changes (Figure 2b,c). This gene, whose expression product is a Golgi membrane protein, caught our attention since it is involved in maintaining Golgi morphology and exocytosis in only a subset of tissues and cell types, particularly secretory tissues.^[^ ^22^ ^]^ Remarkably, mRNA and protein expression levels of Golph3l in AngII‐treated VSMCs were significantly upregulated in an AngII concentration‐dependent manner (**Figure** **3**a,b). Concurrently, the m6A occupancy of Golph3l mRNA in AngII‐treated VSMCs was obviously increased compared to control cells, particularly at site 1 and site 2 of m6A peaks identified and validated by IGV software and MeRIP‐PCR, respectively (Figure 3c), which was accompanied by alteration of Golgi morphology from dispersed Golgi to compacted Golgi, as visualized by immunofluorescence staining with GM130, a Golgi marker, and anti‐Golph3l antibody (Figure 3d). The compacted Golgi structure in AngII‐treated VSMCs was further demonstrated by quantification of the area of the Golgi apparatus (Figure 3d), which is in line with Golgi dynamic change in VSMCs during staurosporin‐induced apoptosis (Figure S2b, Supporting Information). Next, we performed live time‐lapse imaging, which allows the observation of dynamic cellular processes while maintaining the native organization of the cell,^[^ ^31^ ^]^ to monitor the dynamic changes of the Golgi in living VSMCs treated with and without AngII. As shown in Figure 3e, compared with AngII‐untreated cells, the Golgi structure in AngII‐stimulated VSMCs exhibited a marked time‐dependent compaction, as evidenced by decreased Golgi area.

To further investigate the role of Golph3l‐mediated AIP in aortic dissection and aneurysms, we generated VSMC‐specific Golph3l‐inducible deleted mice (smcGolph3l^−/−^) by crossing the Golph3l^fl/fl^ mice with transgenic mice expressing Tgln‐Cre recombinase under control of regulatory sequences from the smooth‐muscle specific Tgln gene (Figure S5a,S5b, Supporting Information). We used BAPN+AngII or HFD+AngII infusion to establish mouse AD and AAA models and demonstrated a dramatic downregulation of Golph3l expression in aortic tissues of AD and AAA models compared to wild type (WT) mice, as confirmed by Western blot analysis (Figure S5c, Supporting Information). Golph3l protein expression in the aorta of smcGolph3l^−/−^ mice was not fully abolished due to the presence of other vascular cells, including endothelial cells, fibroblasts, and macrophages. Compared to littermate control WT mice, smcGolph3l^−/−^ mice had no macroscopic abnormality and death, no differences in body weight (Figure S6a, Supporting Information), blood pressure (Figure S6b, Supporting Information) and cardiac function (Figure S6c,S6d, Supporting Information). Moreover, plasma levels of TNF‐α and TNFSF12F were not significantly different between groups (Figure S6e,S6f, Supporting Information). These findings confirm that the knockout animals do not exhibit intrinsic abnormalities in cardiovascular function and inflammation prior to injury. However, smcGolph3l^−/−^ attenuated AngII‐promoted AD (Figure 3f) and AAA (Figure 3j) formation, along with a significantly reduced aortic diameter (Figure 3g,k). Moreover, the survival rates were also significantly higher in smcGolph3l^−/−^ mice than in WT mice (Figure 3h,l). Simultaneously, HE and masson staining showed that Golph3l deficiency in VSMCs decreased collagen deposition and local thickness of the media in smcGolph3l^−/−^ mice compared with those of WT mice (Figure 3i,m).

Because Golph3l is known to determine Golgi morphology and function,^[^ ^22^ ^]^ we sought to know the effect of SMC‐specific knockout of Golph3l on AngII‐induced AIP, Golgi structure, and TNF‐α secretion. Figure 3n shows successful overexpression of Golph3l in primary mouse VSMCs from smcGolph3l^−/−^ mice by transfecting Golph3l expression plasmid (OE‐Golph3l). We found that AngII‐induced AIP in Golph3l^−/−^ VSMCs was significantly suppressed, whereas the enforced expression of Golph3l in Golph3l^−/−^ VSMCs was able to reverse this effect, as assessed by EdU incorporation assays and cell counting (Figure 3o,p). Accordingly, dispersed Golgi structure in Golph3l^−/−^ VSMCs treated with AngII was rescued by overexpression of Golph3l, as indicated by the immunofluorescence staining with Golgi marker and anti‐Golph3l antibody (Figure 3q). Moreover, this change in Golgi dynamic morphology was further demonstrated in living VSMCs by time‐lapse imaging (Figure 3r). Consistently, the electron microscopy showed that majority of the Golgi existed as a compacted shape in WT VSMCs, however, Golgi structure in Golph3l^−/−^ VSMCs became disperse, but this could be reversed by Golph3l overexpression in Golph3l^−/−^ VSMCs (Figure 3s). Furthermore, Golph3l deficiency also led to a significant decrease in secretion of TNF‐α and TNFSF12 following AngII stimulation. However, overexpression of Golph3l in Golph3l^−/−^ VSMCs rescued the secretion of them (Figure 3t), indicating that Golph3l is essential for the AIP, Golgi dynamics, and TNF‐α signaling in VSMCs. In the further experiment, we demonstrated that Golph3l expression was significantly increased in VSMCs of AD and AAA tissues of clinical samples (Figure S7, Supporting Information).

Together, these results suggest that Golph3l expression is upregulated in AngII‐stimulated VSMCs, and that increased Golph3l expression is essential for AIP and the development of AD and AAA.

### 2.4 The Upregulation of Golph3l by AngII Depends on Gm40097 m6A Modification and Its Interaction with Ythdc1

**Figure 4:**
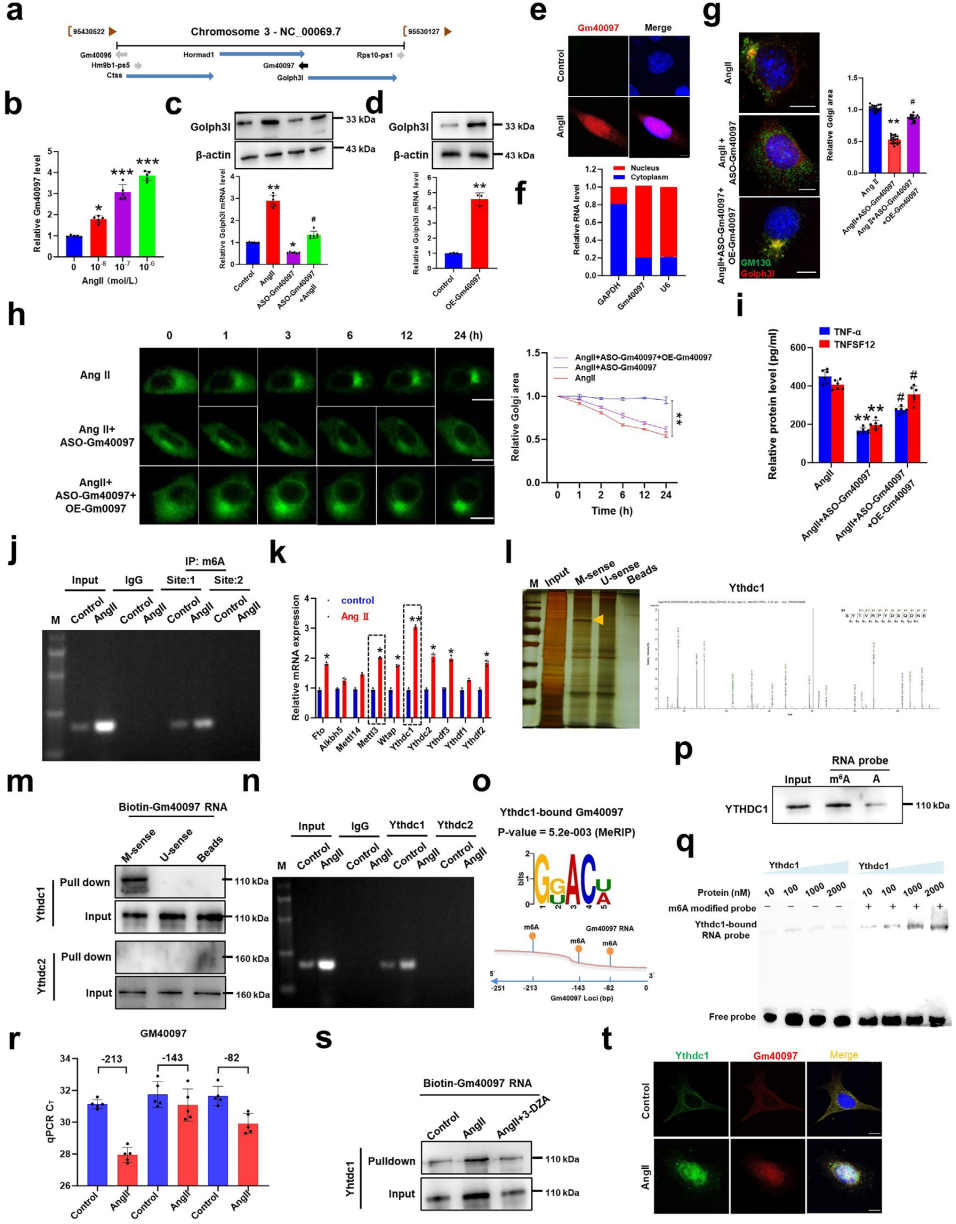
The upregulation of Golph3l by AngII depends on Gm40097 m6A modification. a) Schematic showing Gm40097 gene and its adjacent coding genes on chromosome 3, including Golph3l, Hormad1, Ctss, and Ensa (downloaded from http://genome.ucsc.edu/). b) Mouse VSMCs were stimulated with AngII (0, 10^−8^, 10^−7^, 10^−6^ mol L^−1^) for 24 h. The expression of Gm40097 was analyzed by qRT‐PCR. c) VSMCs were transfected with ASO targeting Gm40097 (ASO‐Gm40097) or nonspecific ASO (Control) for 24 h and then treated with AngII for 24 h. Up: Western blot assay detected Golph3l protein expression. Down: qPCR analysis for Golph3l mRNA expression. d) Western blotting (up) and qPCR (down) detected Golph3l protein and mRNA expression in VSMCs transfected with empty vector or OE‐Gm40097 plasmids. β‐actin was used as a loading control. *p < 0.05, **p < 0.01, and ***p < 0.001 versus control, ^#^ *p* < 0.05 versus AngII. e) RNA fluorescence in situ hybridization (FISH) for Gm40097 localization in AngII‐treated VSMCs and untreated cells as control. Nuclei were counterstained with DAPI. Scale bar = 50 µm. f) qPCR detection for relative expression of GAPDH, Gm40097, and U6 in the nucleus and cytoplasm of AngII‐treated mouse VSMCs. g) VSMCs were transfected with ASO‐Gm40097 or ASO‐Gm40097+OE‐Gm40097 plasmids for 24 h, and then treated with AngII. Golgi morphology was visualized as described in (Figure 3d). Right: Statistical analysis of Golgi area ratio relative to AngII or AngII+ASO‐Gm40097. The data represent mean ± SEM of 3 independent experiments in which 50 cells were analyzed. **p < 0.01 versus AngII, and ^#^ *p* < 0.05 versus AngII+ASO‐Gm40097. h) Time‐lapse image showing the Golgi dynamics in live mouse VSMCs treated with indicated constructs. Right: Quantitative analysis of Golgi‐Tracker green area. **p < 0.01 versus AngII. i) ELISA assay showing the relative levels of TNF‐α and TNFSF12 in cultured medium of VSMCs treated as described in (g). The data represent mean ± SEM of 3 independent experiments. **p < 0.01 versus AngII, ^#^ *p* < 0.05 versus AngII+ASO‐Gm40097. j) MeRIP assay showing an increased m6A modification of Gm40097 in AngII‐treated mouse VSMCs. IgG was used as negative control. k) qRT‐PCR detection of differential expression of a subset of genes involved in the m6A modification, including writer, eraser, and reader‐related genes in AngII‐treated mouse VSMCs. Data represent mean ± SEM of 3 independent experiments. *p < 0.05 and **p < 0.01 versus control, *n* = 3. l) Silver‐staining gel image of RNA pull‐down experiment using Biotin‐labeled Gm40097 with and without m6A modification in mouse VSMCs treated with AngII for 24 h. The arrowhead indicates Ythdc1 identified by liquid chromatography‐tandem mass spectrometry (LC‐MS) method (Right). m) RNA pull‐down assay demonstrating the association between Gm40097 and Ythdc1, not Ythdc2. n) RIP experiments showing the binding of Gm40097 with Ythdc1, not Ythdc2. o) The canonical m6A motif GGACU is enriched in the Gm40097 RNA sequences, including three m6A modification sites (m6A‐213, m6A‐143, and m6A‐82) identified by MeRIP‐seq. p,q) RNA pull‐down p) and EMSA analysis q) showing Ythdc1 binding to Gm40097 with m6A modification. *n* = 3 biologically independent experiments. r) qPCR showing the m6A modification level on three sites of GM40097 in the immunoprecipitate of mouse VSMCs treated or not with AngII. **p < 0.01 versus control. *n* = 5 for each group. s) VSMCs were treated with 3‐DZA (inhibitor of m6A) for 24 h, and then stimulated with AngII (10^−7^ mol L^−1^) for 24 h. RNA pull‐down analysis showing Ythdc1 binding to Gm40097 with m6A modification. *n* = 3 biologically independent experiments. t) In situ hybridization of Gm40097 (red) combined with Ythdc1 staining (green) were performed and observed by confocal microscopy. Scale bar = 10 µm.

Next, we investigated the upstream regulatory mechanism leading to the upregulation of Golph3l expression in AngII‐stimulated VSMCs. It is well known that neighboring genes may be coexpressed when they share the same open or closed chromatin conformation,^[^ ^32^ ^]^ and their expressions may be regulated by adjacent genes or noncoding RNAs.^[^ ^33^ ^]^ To determine whether Golph3l expression is regulated by its neighboring genes, we mapped the adjacent genes of Golph3l on chromosome 3 (**Figure** **4**a) and examined the effect of treating VSMCs with different concentrations of AngII on their expressions. qRT‐PCR analysis showed that lncRNA Gm40097 expression was highly upregulated in a AngII concentration‐dependent manner (Figure 4b), rather than other adjacent genes, including hormad1, Ctss, and Rps‐ps1 (Figure S8, Supporting Information). Further, we conducted loss‐ and gain‐of‐function experiments to assess the influence of Gm40097 on Golph3l expression. The results revealed that targeted knockdown of Gm40097 by its antisense oligonucleotides (ASO) transfection^[^ ^34^ ^]^ led to a significant decrease in Golph3l expression regardless of AngII treatment (Figure 4c). Reversely, overexpression of Gm40097 in VSMCs by Gm40097‐expressing plasmid significantly increased Golph3l expression (Figure 4d). Additionally, the nuclear distribution of Gm40097 detected by fluorescence in situ hybridization (FISH) was markedly increased in AngII‐stimulated VSMCs compared to control cells (Figure 4e). A similar result was obtained by qRT‐PCR analysis of cytoplasmic‐to‐nuclear expression ratios of Gm40097 (Figure 4f). We then investigated the effects of knockdown and overexpression of Gm40097 in VSMCs on Golgi morphology by immunofluorescence staining with Golgi marker GM130 and anti‐Golph3l antibody. The results showed that Gm40097 knockdown by ASO‐Gm40097 significantly reduced AngII‐induced Golgi compaction, whereas its overexpression rescued Golgi morphology in Gm40097‐depleted VSMCs upon AngII treatment (Figure 4g). Likewise, time‐lapse imaging analysis revealed that Gm40097 depletion in VSMCs attenuated AngII‐induced Golgi compaction, whereas the decreased compaction was reversed by Gm40097 overexpression, as evidenced by altered Golgi area (Figure 4h). Correspondingly, the secretion of TNF‐α and TNFSF12 into culture medium was significantly reduced in Gm40097‐depleted VSMCs treated with AngII, and the decreased secretion of the two factors was rescued by overexpression of Gm40097 (Figure 4i). Taken together, these observations indicate that Gm40097 is essential for the upregulation of Golph3l expression and the activation of TNF‐α signaling.

Because the above MeRIP‐seq analysis showed a significantly increased m6A methylation of Gm40097 in AngII‐treated versus AngII‐untreated VSMCs (Figures 2b and 3c), we wondered whether Gm40097 regulation of Golph3l expression depends on Gm40097 m6A modification. Indeed, the results of methylated RNA immunoprecipitation (MeRIP) with m6A antibody followed by RT‐qPCR revealed an increased level of Gm40097 m6A methylation caused by AngII (Figures 3c and 4j). Considering that RNA m6A modification is mediated by writers (methyltransferases), erasers (demethylases), and reader proteins,^[^ ^35^ ^]^ we investigated the effects of treating VSMCs with AngII on the expression of these m6A‐modifying enzymes, including methyltransferases (Mettl3, Mettl14, and Wtap), demethylases (Alkbh5 and Fto), and reader proteins (Ythdc1‐2, Ythdf1‐3). As shown in Figure 4k, the expression of reader protein Ythdc1 was greatly increased in AngII‐treated VSMCs compared to control cells. Similarly, we obtained the RNA‐seq data of m6A regulators Ythdc1 and Mettl3 from RNA‐seq public database (GEO number: GSE140947), and found that mRNA expression of Ythdc1 and Mettl3 is also upregulated in VSMCs of human aortic aneurysm sample (Figure S9, Supporting Information). We further performed RNA pull‐down assays with synthetic biotinylated Gm40097 with or without m6A methylation to examine whether Gm40097 interacts with Ythdc1. Pulled‐down proteins were separated by SDS‐PAGE and visualized by silver staining, and an ≈130‐kDa protein was detected in the precipitates pulled‐down by m6A‐methylated Gm40097, which was identified to be m6A reader Ythdc1 by mass spectrometry analysis of this band (Figure 4l), suggesting that Ythdc1 could probably participate in Gm40097 regulation of Golph3l expression induced by AngII through recognizing m6A‐methylated Gm40097. In further experiments, the interaction between m6A methylated Gm40097 and Ythdc1, but not Ythdc2, was validated by RNA pull‐down followed by immunoblotting (Figure 4m) as well as by RIP followed by qRT‐PCR (Figure 4n).

Furthermore, we found that there existed some canonical m6A motifs‐GGACU in Gm40097 sequence by analysis of MeRIP‐seq data (*p* = 5.2 × 10^−3^, Figure 4o). To determine whether the binding of Ythdc1 to m6A motif GGACU depends on its m6A methylation, we conducted RNA pull‐down followed by immunoblotting (Figure 4p) and electrophoretic mobility shift assay (EMSA) (Figure 4q) using a pair of biotin‐conjugated RNA probes (conserved 30‐base Gm40097 sequence) flanking the GGACU motif with or without m6A modification. The results showed that, compared with the unmethylated RNA probe, the methylated probe exhibited much stronger capacity to bind Ythdc1 protein (Figure 4p,q). In addition, we employed a single‐base elongation‐and ligation‐based qPCR amplification method (SELECT) as reported previously^[^ ^36^ ^]^ to robustly identify the specific m6A‐methylated site (213) in Gm40097 sequence. qRT‐PCR results showed a significant m6A modification on 213 site in VSMCs treated with AngII compared to control cells (Figure 4r), suggesting the m6A modification of Gm40097 is site‐specific. Moreover, the interaction between Gm40097 and Ythdc1 in AngII‐stimulated VSMCs was weakened by pretreating VSMCs with m6A methylation inhibitor 3‐DZA (Figure 4s) or by knockdown of Mettl3 expression with Mettl3‐specific siRNA (Figure S10, Supporting Information), as verified by biotin‐Gm40097 pull‐down assays. In line with these results, immunofluorescence in situ hybridization (imFISH) detection also revealed that Gm40097 and Ythdc1 were distributed across the entire cell in AngII‐untreated cells, but they were largely translocated to the nuclear area after Ang II treatment for 1 h, and were colocalized in the nucleus (Figure 4t). Taken together, these data demonstrate that the interaction of Gm40097 with Ythdc1 depends on m6A methylation of Gm40097 and is essential for Golph3l expression.

### 2.5 Ythdc1 Facilitates Chromatin Accessibility of the Golph3l Promoter Region by Interacting with p300 and Klf5 in an m6A Modified Gm40097‐Dependent Manner

**Figure 5:**
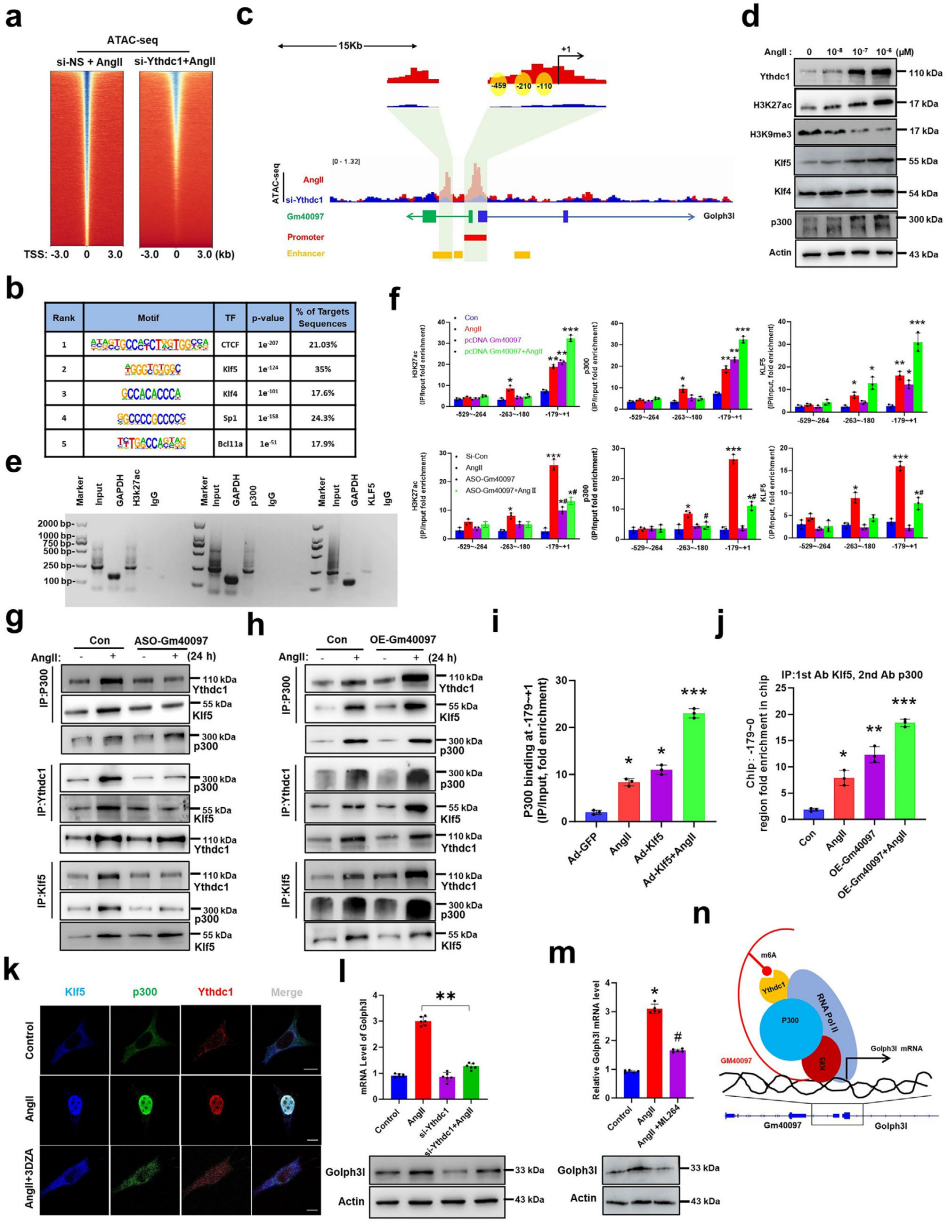
Ythdc1 facilitates chromatin accessibility of the Golph3l promoter region by interacting with p300 and Klf5. a) ATAC‐seq signaling in mouse VSMCs transfected with siRNA targeting Ythdc1 (si‐Ythdc1) or si‐NS and then stimulated with AngII for 24 h. b) Results of motif enrichment analysis with HOMER on Ythdc1 signaling in ATAC‐seq peaks. c) Genome browser snapshot displaying ATAC‐seq, H3K27ac‐ChIP‐seq, and Klf5‐ChIP‐seq tracks in the intergenic region between Gm40097 and Golph3l locus in AngII‐treated VSMCs transfected with si‐NS or si‐Ythdc1. Red and blue rectangles, respectively, represent promoter and enhancer regulatory elements predicted by UCSC. d) Mouse VSMCs were stimulated with AngII (0, 10^−8^, 10^−7^, 10^−6^ mol L^−1^) for 24 h. The expression of Ythdc1, H3K27ac, H3K9me3, Klf5, Klf4, and p300 was analyzed by Western blotting. e) ChIP‐PCR detected the binding of H3K27Ac, Klf5, and p300 to the cotranscriptional initiation region of Gm40097 and Golph3l in AngII‐treated VSMCs. f) ChIP analysis of H3K27ac, p300, and Klf5 occupancy at yellow areas of the Golph3l promoter in cells transfected with pcDNA‐Gm40097 (Top) or ASO‐Gm40097 (Down) for 24 h, and then treated with AngII for an additional 12 h (*n* = 3). *p < 0.05, **p < 0.01, and ***p < 0.001 versus Control. ^#^ *p* < 0.05 versus AngII. g,h) Mouse VSMCs were transfected with ASO‐Gm40097 g) and pcDNA‐Gm40097 (OE‐Gm40097) h) or their corresponding controls for 24 h, and treated with AngII for 24 h. Cell lysates were immunoprecipitated with anti‐Ythdc1, anti‐p300, or anti‐Klf5 antibodies and then immunoblotted with an antibody against Ythdc1, p300, and Klf5. i) ChIP‐PCR detected Klf5 occupancy within the co‐transcriptional initiation region of Gm40097 and Golph3l in AngII‐treated VSMCs infected with Ad‐GFP or Ad‐Klf5 for 24 h. j) Cross‐linked chromatin of control and AngII‐treated VSMCs infected with empty vector or OE‐Gm40097 was immunoprecipitated with anti‐Klf5 antibody, followed by brief treatment with 0.5% SDS‐containing buffer. The released material was subjected to a second immunoprecipitation with anti‐p300 antibody. Immunoprecipitated DNA containing the ‐179/+1 bp of the Golph3l and Gm40097 promoter regions was amplified by PCR (*n* = 3). k) VSMCs grown on cell culture inserts were treated with AngII or AngII+3‐DZA for 24 h, and then immunofluorescence staining for Klf5 (blue), p300 (green), and Ythdc1 (red) was performed and observed by confocal microscopy. l) The mRNA (top) and protein (down) levels of Golph3l were detected by qRT‐PCR and Western blotting, respectively, in mouse VSMCs transfected with si‐Ythdc1 and then treated with AngII for 24 h. **p < 0.01 versus AngII. *n* = 3 for each group. m) The mRNA (top) and protein (down) levels of Golph3l were detected by qRT‐PCR and Western blotting, respectively, in AngII‐stimulated mouse VSMCs treated with Klf5 inhibitor ML264 for 24 h. *p < 0.05 versus Control, ^#^ *p* < 0.05 versus AngII. n)The proposed model of AngII‐induced m6A‐modified Gm40097 mediates Ythdc1/p300/Klf5 transcriptional complex formation at intergenic region between Gm40097 and Golph3l.

The above‐described results suggest that the increased interaction of m6A‐modified Gm40097 with Ythdc1 is implicated in AngII‐induced AIP in VSMCs. Considering that Ythdc1 can increase chromatin accessibility and activates transcription in an m6A‐dependent manner,^[^ ^37^, ^38^ ^]^ we wondered whether Ythdc1 is recruited to the promoter of Golph3l by interacting with m6A‐modified Gm40097 to modulate chromatin accessibility, subsequently leading to the activation of Golph3l transcription by AngII in VSMCs. To verify this, we performed the nuclei extraction from Ythdc1‐depleted mouse VSMCs followed by the assay for transposase accessible chromatin sequencing (ATAC‐seq). Under the dual criteria of |log2 fold‐change| ≥ 1 and *p*‐value < 0.05, we identified 12702 chromatin accessible regions (peaks) related to nearby genes and 3067 differentially accessible regions (DARs) between Ythdc1‐depleted and control cells. More importantly, Ythdc1‐depleted VSMCs exhibited a widespread decrease of chromatin accessibility after AngII stimulation compared to control cells (**Figure** **5**a; and Figure S11a, Supporting Information). Moreover, the decreased ATAC‐seq signals were abundantly enriched proximal to the transcription start site (≤1 kb) (Figure S11b, Supporting Information). Using published Klf5 and H3K27ac ChIP‐seq datasets (GSE80812 and GSE145964), we found that the enhancer or promoter region of downstream target genes regulated by Klf5 showed a particularly strong H3K27ac signal, and that Klf5 ChIP‐seq peaks and H3K27ac ChIP‐seq peaks showed a similar trend to that of ATAC‐seq (Figure S11c,d, Supporting Information). Because the interplay between chromatin accessibility and transcription factor (TF) binding is fundamental to activate transcription, we then performed the TF motif enrichment analysis to identify the potential TFs bound to these motifs, and found that the CTCF, Klf, and Sp1 motifs were highly enriched in DARs in context of AngII treatment, particularly Klf family (Klf5, Klf4) motifs been identified in 35% and 17.6% of these DARs, respectively (Figure 5b). Furthermore, the opening of the chromatin induced by AngII at the Golph3l promoter region was significantly reduced by Ythdc1 knockdown, as indicated by Integrative Genomics Viewer (IGV) analysis (Figure 5c). Interestingly, we observed a consistent change between ChIP‐seq signals of Klf5 and H3K27ac in a region adjacent to the copromoter shared between Gm40097 and Golph3l genes, and this region was predicted by UCSC database to be able to act as a proximal‐enhancer (yellow region). Simultaneously, using the JASPAR database and TFBSTools, we found that there exist the three potential binding sites for Klf5 in the copromoter (including –459, –210, and –110 sites) (Figure 5c). These results imply that Gm40097, as a potential enhancer activated by Klf5, mediates Ythdc1 regulation of chromatin accessibility of the Golph3l promoter. In line with the ATAC‐seq, ChIP‐seq, and bioinformatics assay, the expression of Ythdc1, H3K27ac, Klf4, Klf5, and p300 was continuously increased, whereas the trimethylation of histone H3 lysine 9 (H3K9me3), a known transcriptional repression marker, was decreased in AngII‐stimulated VSMCs in an AngII concentration‐dependent manner (Figure 5d). To elucidate the mechanism underlying the activation of the copromoter of Golph3l and Gm40097 by Klf5, the ChIP‐PCR assay was used to detect the enrichment of H3K27ac, p300, and Klf5 at the copromoter region. As shown in Figure 5e, H3K27ac, p300, and Klf5 were highly recruited to the copromoter region in AngII‐stimulated VSMCs. We further mapped the binding sites for H3K27ac and demonstrated that H3K27ac was highly concentrated in the region between –179 and +1 bp of the Golph3l promoter in AngII‐treated VSMCs. Gm40097 overexpression significantly increased the enrichment of H3K27ac in this region, whereas Gm40097 depletion decreased its enrichment under AngII treatment. Combination of Gm40097 overexpression and AngII treatment further enhanced the enrichment of H3K27ac. Also, p300 and Klf5 were highly concentrated in the same region as H3K27ac, accompanied by increased or decreased enrichment in the context of GM40097 overexpression or knockdown, respectively (Figure 5f).

Based on the above results, we speculated that Angll‐induced Ythdc1‐Klf5‐p300 complex formation and its binding to the copromoter of Gm40097 and Golph3l might lead to the activation of Golph3l transcription. To test our hypothesis and investigate the functional significance of Gm40097 in the complex formation, we performed a coimmunoprecipitation assay and found that treating VSMCs with AngII substantially increased the levels of Ythdc1 and Klf5 present in anti‐p300 immunoprecipitates, which was largely neutralized by depleting Gm40097 in VSMCs with ASO‐Gm40097. Consistently, the similar results were obtained by immunoprecipitating with anti‐Ythdc1 or anti‐Klf5, followed by immunoblotting with anti‐Klf5 and anti‐p300 or anti‐Ythdc1 and anti‐p300 (Figure 5g). Conversely, overexpression of Gm40097 in VSMCs further increased the AngII‐induced interactions among Ythdc1, Klf5, and p300 (Figure 5h). Moreover, overexpression of Klf5 mediated by adenovirus vector in VSMCs enhanced the binding of p300 to ‐179 and +1 bp of the copromoter region (Figure 5i). Furthermore, we performed a sequential ChIP analysis in which VSMC chromatin was immunoprecipitated with anti‐Klf5 first and then with anti‐p300. As shown in Figure 5j, AngII significantly increased the recruitment of the Klf5 and p300 to ‐179 and +1 bp of the copromoter compared with untreated cells, and Gm40097 overexpression further enhanced their interaction. Additionally, the results of multiplex immunofluorescence staining revealed that Ythdc1, Klf5, and p300 were distributed across the entire cell before AngII treatment, but translocated to the nuclear area in AngII‐treated VSMCs for 24 h, and were colocalized in the nucleus (Figure 5k), consistent with the immunoprecipitation results. As expected, 3‐DZA treatment largely abrogated the AngII‐induced their nuclear translocation (Figure 5k). To provide further evidence supporting the above findings, we knocked down Ythdc1 by si‐Ythdc1 or repressed Klf5 by its inhibitor ML264 and then determined the effects of destroying the Ythdc1‐Klf5‐p300 complex on Golph3l expression. The results showed that depletion of Ythdc1 in VSMCs significantly reduced Golph3l expression induced by AngII at both mRNA and protein levels (Figure 5l). In line with this result, the expression of Golph3l was also significantly downregulated by Klf5 inhibitor in AngII‐treated VSMCs (Figure 5m). Collectively, these results suggest that Gm40097‐mediated formation of Ythdc1‐Klf5‐p300 complex on the Golph3l promoter is essential for the activation of Golph3l transcription, providing a novel lncRNA m6A modification‐dependent regulatory mechanism of chromatin remodeling and transcriptional activation (Figure 5n).

### 2.6 Klf5 Activates the Promoter and Enhancer of the Gm40097 and Golph3l by Recruiting p300‐Catalyzed H3K27ac

**Figure 6:**
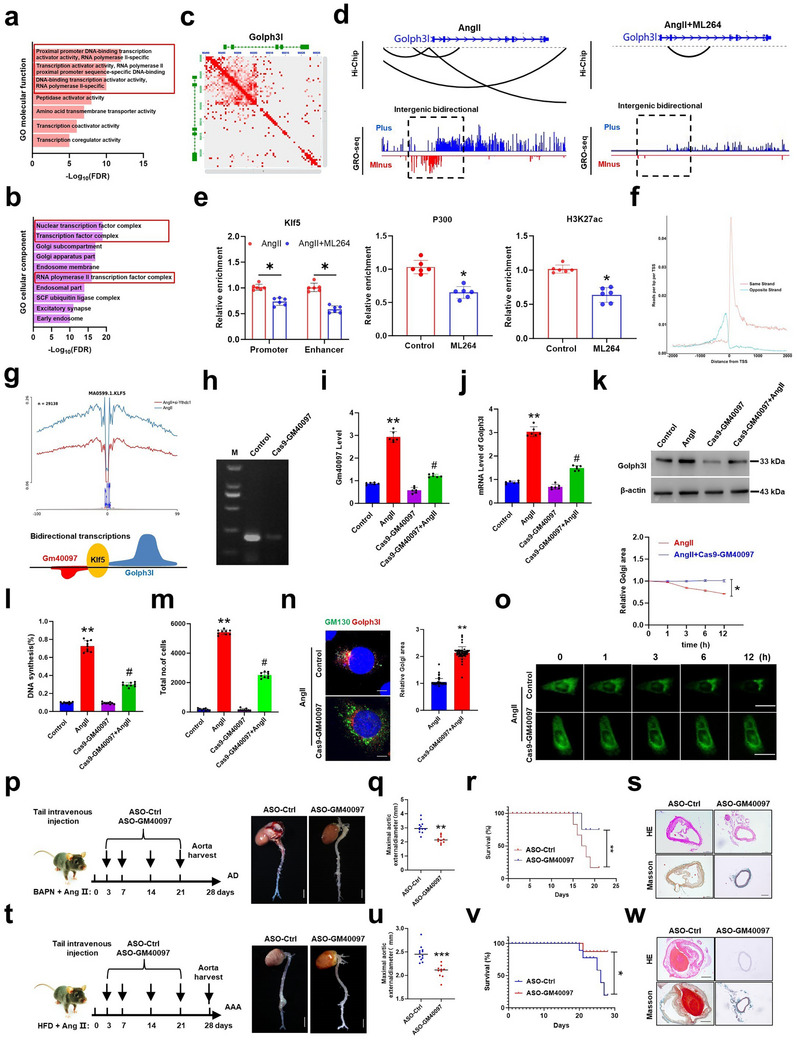
Klf5 activates the promoter and enhancer of the Gm40097 and Golph3l by recruiting H3K27ac. a,b) The GO analysis for classification of the significant enhancer/promoter (E/P) loops using H3K27ac HiChIP assays a) and the corresponding nascent transcripts detected by GRO‐seq b) based on biological process. c) HiChIP contact map depicting chromatin looping between Golph3l promoter and adjacent Gm40097. d) Genome browser tracks of HiChIP analysis of AngII‐treated VSMCs showing corresponding H3K27ac signals (up) and nascent transcripts (peaks) (down). Dashed box and black curve denote the chromatin loop. GRO‐seq signal on +/− strand is indicated by blue and red, respectively. e) Mouse VSMCs were treated or not with Klf5 inhibitor ML264 and then stimulated with AngII. CUT&RUN‐qPCR analysis detected the bindings Klf5 to the promoter and enhancer region of Golph3l as well as p300 and H3K27ac enrichment at the Golph3l promoter. *N* = 6 for each group. *p < 0.05 versus Control. f) Average GRO‐seq signal mapping a 2 kb window around the transcription start site for charactering bidirectional nascent RNAs. g) Aggregated footprint signal of transcription factor Klf5 with a significant decreased footprint signal in AngII‐treated versus AngII+si‐Ythdc1‐treated VSMCs. Down: Schematic models of Klf5‐mediated transcription of Gm40097 and Golph3l pre‐mRNA. h) The Gm40097 binding region inside the upstream of the Golph3l promoter was deleted using CRISPR interference, the PCR detected the knockout efficiency. i,j) qRT‐PCR detected Gm40097 RNA i) and Golph3l mRNA j) expression in the Cas9‐Gm4009‐transfected VSMCs treated or not with AngII. (*n* = 3–5 independent experiments). Data are expressed as mean ± SEM; **p < 0.01 versus control, #*p* < 0.05 versus AngII. k) Western blotting detected Golph3l expression in the Cas9‐Gm40097‐transfected VSMCs treated or not with AngII. l,m) An in vitro coculture model was used where untreated mouse VSMCs were seeded in the top compartment, and Cas9‐Gm40097‐transfected VSMCs treated or not with AngII in the bottom compartment. Percentage of EdU‐positive cells l) and total number of cells m) in top compartment are shown as histograms. The data represent mean ± SEM of 3 independent experiments. **p < 0.01 versus control, ^#^ *p* < 0.05 versus AngII. n) Immunofluorescence staining of GM130, Golph3l and nuclei (DAPI) was performed to visualize Golgi morphology in Cas9‐Gm40097‐transfected VSMCs treated with AngII. Right: Statistical analysis of Golgi area relative to AngII treatment. Data represent mean ± SEM of 3 independent experiments in which 50 cells were analyzed. *p < 0.05 versus AngII. o) Time‐lapse images showing the Golgi dynamics in live VSMCs transfected or not with Cas9‐Gm40097 and then treated with AngII. Up: Quantitative analysis of Golgi‐Tracker green fluorescence area. *p < 0.05 versus AngII. *n* = 50 for each group. p,t) Experimental procedures for establishing mouse models of AD p) and AAA t) and intervening with ASO‐Gm40097. Representative photographs of mouse aortas ex vivo (Scale bars = 2.5 mm). q,u) Statistical analysis of the maximal aortic external diameter in AD (up) and AAA (down) models treated or not with ASO‐Gm40097. Data represent the mean ± SD. **p < 0.01 and ***p < 0.001 versus ASO‐Ctrl. r,v) The survival rate analyzed by log‐rank test in AD (up) and AAA (down) models treated or not with ASO‐Gm40097. Data represent the mean ± SD. *p < 0.05, and **p < 0.01 versus ASO‐Ctrl. *n* = 12 for ASO‐Ctrl group, *n* = 10 for ASO‐Gm40097 group. s,w) HE‐stained (top row) and Masson‐stained (bottom row) sections of the aortas from mouse AD (up) and AAA (down) models treated or not with ASO‐Gm40097. Scale bars = 50 µm.

Klf5 has been shown to functionally interact with chromatin modifying cofactors, such as p300^[^ ^39^ ^]^ and HDAC1,^[^ ^40^ ^]^ and our previous studies demonstrated that Klf5 is involved in the vascular remodeling response to stress,^[^ ^12^, ^13^, ^41^ ^]^ so we reasoned that Klf5 is responsible for the binding of Gm40097 to the Golph3l promoter by recruiting the p300‐acetylated H3K27ac to this region. Through integrated analysis of H3K27ac HiChIP‐seq and global run‐on sequencing (GRO‐seq) data in VSMCs treated with Klf5 inhibitor ML264 and then stimulated with AngII, we found that ML264 efficiently reduced AngII‐induced high‐order chromatin organization (AngII: 169174 vs AngII+ML264: 54440) (Figure S12a,S12b, Supporting Information; 500 kb resolution, balanced normalization). GO analysis revealed that the significantly decreased enhancer‐promoter interaction‐related GO terms were enriched after Klf5 was inhibited in VSMCs by ML264, such as proximal promoter DNA‐binding transcription activator activity, RNA polymerase II‐specific transcription activator activity, RNA polymerase II proximal promoter sequence‐specific DNA‐binding activity, etc. (**Figure** **6**a). In parallel, GO analysis of nascent transcripts of both pre‐mRNAs and enhancer RNAs (eRNAs) detected by GRO‐seq at a genome‐wide level also revealed that the decreased nuclear transcription factor complex and RNA polymerase II transcription factor complex, which play a vital role in chromatin accessibility, were included in the enriched pathways (Figure 6b). Interestingly, obviously reduced GO terms related to Golgi subcompartment and Golgi apparatus parts were presented in Klf5‐inhibited VSMCs treated with AngII. In line with GO analysis of HiChiP‐seq and GRO‐seq data, peak annotation of the Integrative Genomics Viewer (IGV) also confirmed an AngII‐induced interaction between Gm40097 enhancer and the Golph3l promoter, and AngII treatment increased nascent transcription of both Golph3l pre‐mRNA and enhancer RNA Gm40097, showing a characteristic of bidirectional transcription. Remarkably, pharmacological inhibition of Klf5 by ML264 impaired their interaction (Figure 6c–e). Further, we performed CUT&RUN qPCR to examine the change in Klf5 binding to its cis‐binding sites within Golph3l regulatory region after Klf5 inhibition. The results showed that ML264 treatment significantly weakened the binding of Klf5 to the promoter and enhancer regions of Golph3l, which was concurrently accompanied by decreased binding of H3K27ac and p300 to this region (Figure 6e,f), Consistently, ATAC‐seq based footprinting analysis of differentially accessible regions indicated that Klf5 binding motif showed a clear increase in footprint depth in AngII‐treated VSMCs, but Ythdc1 knockdown reduced this strong signal (Figure 6g). These results suggest that transcription activation of the Golph3l promoter by AngII‐induced Klf5 occupancy at intergenic region depends on the interaction of Gm40097 enhancer with the Golph3l promoter.

To provide further evidence that Gm40097 enhancer plays a key role in regulating Golph3l expression, Golgi morphology, TNF‐α signaling, and AIP in VSMCs, we designed a single‐chain guide RNA (sgRNA) complementary to the Gm40097 enhancer to conduct CRISPR interference (CRISPRi), which represses transcription by blocking transcriptional initiation or elongation with a catalytically dead Cas9.^[^ ^42^ ^]^ The results of qRT‐PCR showed a dramatic downregulation of Gm40097 expression after CRISPRi‐based knockdown of the Gm40097 enhancer in VSMCs (Figure 6h), which leads to a significantly decreased AngII‐induced upregulation of Gm40097 expression (Figure 6i), accompanied by a marked decrease in Golph3l mRNA (Figure 6j) and protein (Figure 6k) levels. In the further experiment, we utilized a cell coculture system, as shown in Figure 1j, to explore the effect of Gm40097 on AngII‐induced AIP in VSMCs, in which AngII‐untreated VSMCs were seeded in the upper chamber and AngII‐stimulated or CRISPRi‐treated VSMCs in the lower chamber. EdU incorporation assays and cell counting demonstrated that Cas9‐Gm40097 significantly reduced the proliferation of VSMCs in the top compartment compared to control VSMCs (Figure 6l,m). Meanwhile, the compacted Golgi structure in AngII‐stimulated VSMCs was transformed into the dispersed structure after CRISPRi silencing of Gm40097 enhancer, as visualized by confocal microscope (Figure 6n) and time‐lapse imaging analysis (Figure 6o), respectively. Collectively, the above results suggest that Klf5 mediates the interaction of Gm40097 enhancer with the Golph3l promoter by recruiting H3K27ac, contributing to chromatin remodeling at the copromoter region of Gm40097 and Golph3l, which facilitates the transcriptional activation of Golph3l in VSMCs.

To evaluate the functional significance of targeting Gm40097 in the treatment of vascular remodeling diseases, we exploited the mouse models of AD and AAA to investigate the therapeutic efficacy of intravenous administration of ASO‐Gm40097 in vivo. As shown in Figure 6p,t, compared with ASO‐Ctrl, administration of ASO‐Gm40097 was able to effectively alleviate AngII‐induced AD and AAA formation. This intervention also significantly reduced the aortic external diameter (Figure 6q,u) and prolonged the overall survival time in AngII‐induced AD and AAA mice (Figure 6r,v). Moreover, AngII‐induced aortic damage was obviously mitigated by ASO‐Gm40097 treatment, as shown by HE and masson staining (Figure 6s,w). In addition, Golph3l expression and AngII‐induced AIP of VSMCs were consistently attenuated by ASO‐Gm40097 treatment in AngII‐induced AD (Figure S12c,e, Supporting Information) and AAA (Figure S12d,f, Supporting Information) mice.

### 2.7 Single Nucleotide Polymorphism (rs6700022 A‐to‐C Transition) in the Upstream Regulatory Region of Golph3l Gene Leads to Increased Expression of Golph3l and is Associated with the Development of AD and AAA

**Figure 7:**
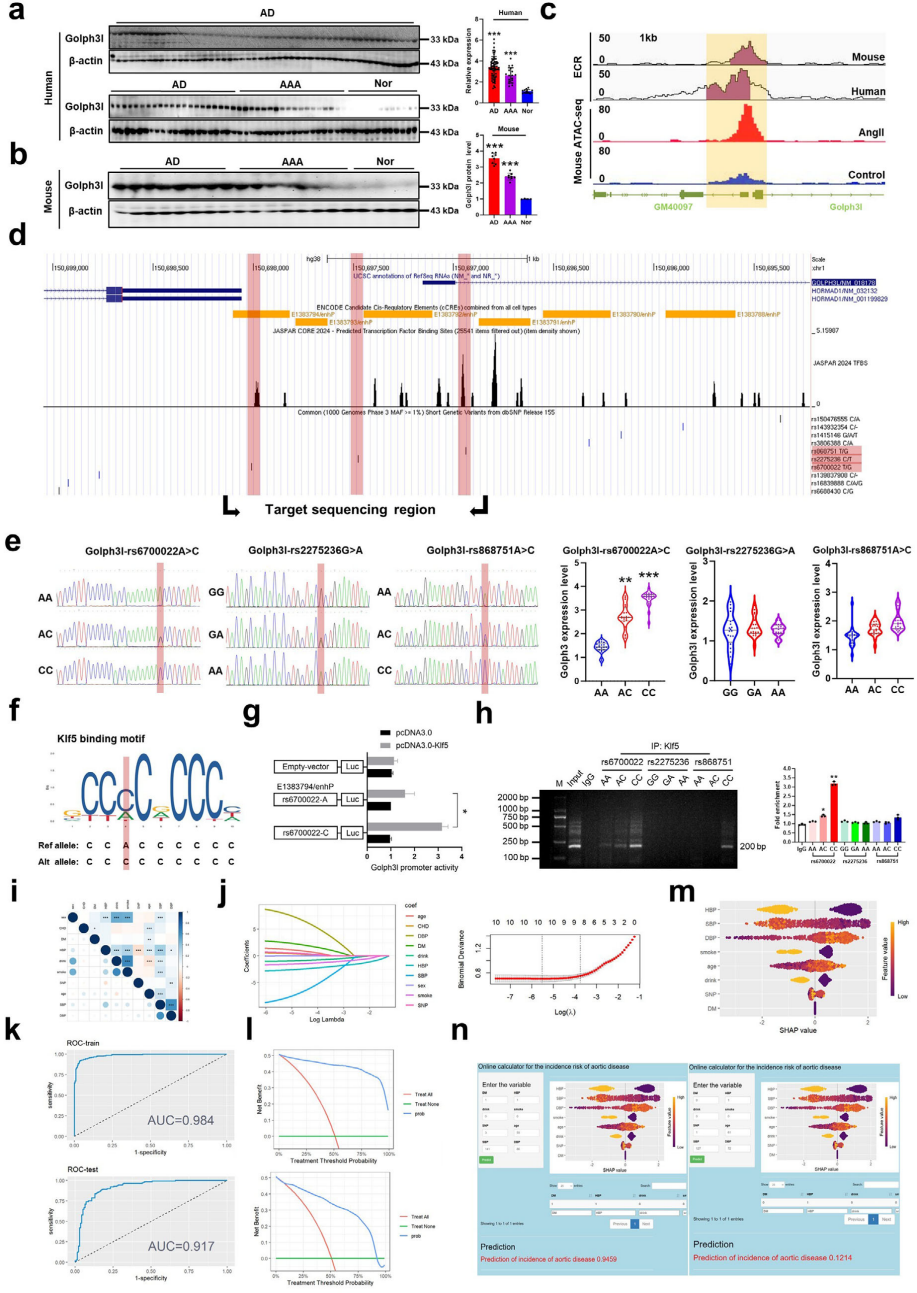
SNP (rs6700022 A‐to‐C transition)‐mediated upregulation of Golph3l expression is associated with the development of AD and AAA. a,b) Western blot analysis detected Golph3l expression (normalized to β‐actin expression) in human AD (*n* = 71) and AAA (*n* = 18) tissues and normal aortas (*n* = 12) as well as in mouse AD (*n* = 8) and AAA (*n* = 8) tissues and normal aortas (*n* = 4). Right: Band intensities were measured and normalized to β‐actin. ***p < 0.001 versus Nor. c) Conservation between mouse and human Gm40097 as identified by the ECR Browser (Above, areas in red indicate greater than 77% conservation, scale from 50% to 100%). Below: ATAC‐seq data of AngII‐stimulated mouse VSMCs are shown. d) Proximal enhancer, single nucleotide polymorphism (SNP), and Klf5‐ChIP‐seq peaks (JASPAR 2024 TFBS) in the upstream regulatory region of Golph3l gene. The data were downloaded from UCSC. e) Genomic DNA was isolated from peripheral blood nucleated cells of patients with AD, AAA, and healthy subjects. DNA sequences within the upstream regulatory region of Golph3l gene were sequenced. Existence of three SNPs with different genotypes was proved in these subjects, and then the total number of subjects with different genotypes for each SNP was quantified. Data represent mean ± SEM, **p < 0.01 and ***p < 0.001 versus AA genotype. f) The altered allele C in rs6700022 sequence created a Klf5 binding motif. g) Relative reporter gene activity of the constructs containing the WT or Mut allele of rs6700022 in human VSMCs. *p < 0.05 versus rs6700022‐A. h) ChIP was performed with anti‐Klf5 or rabbit IgG antibody in human aortic tissues carrying different genotypes of the three SNPs, and then immunoprecipitated DNA was amplified by PCR using primers spanning the upstream regulatory region of Golph3l gene. Values are represented as mean ± SEM of three independent experiments. *p < 0.05 and **p < 0.01 versus IgG (Student's *t*‐test). i) Heat map showing the correlation among variables by Spearman correlation analysis. j) Lasso regression‐based variable screening including variation characteristics of variable coefficients and the process of selecting the optimal value of the parameter λ by the cross‐validation method. k) Receiver operating characteristic curves: patients with AD for SNP in two cohorts. l) Decision curve analysis (DCA) assessed the clinical benefit of XGBoost model in two cohorts. m) SHapley Additive exPlanations (SHAP) summary plots order the features based on their contribution to predictive ability of model. Each plot is made up of individual points from the training dataset with a higher value being darker purple and a lower value being more yellow. If the dots on one side of the middle line are more purple or yellow, this suggests that the values are increasing or decreasing, respectively, moving the prediction in that direction. n) The established the AD postoperative short‐term death prediction website for testing the results of two patients (https://golph3lpredicted.shinyapps.io/document/).

To evaluate the potential clinical implication of our findings, we examined the expression of Golph3l gene in human and mouse AD and AAA tissues and found that Golph3l expression was substantially upregulated in these diseased aortic tissues of humans (**Figure** **7**a) and mice (Figure 7b) compared to their corresponding controls. Considering that single nucleotide polymorphisms (SNPs) in the upstream regulatory region of Golph3l gene may affect its promoter activity and expression level, and that multiple noncoding variants, including promoter/enhancer variants, were associated with thoracic aortic aneurysms and dissections,^[^ ^43^ ^]^ we ought to know whether there exist several functional SNPs in the region upstream of Golph3l transcription start site. To do this, we first used the ECR Browser^[^ ^44^ ^]^ and ATAC‐seq signals to analyze the base sequence homology between mouse Gm40097 and human Gm40097. The results showed that Gm40097 enhancer was highly conserved (>75%) between humans and mice, implying that the regulatory network upstream of Golph3l gene may be deeply conserved (Figure 7c). Next, using the data from ENCODE (Encyclopedia of DNA Elements) project in UCSC genome browser, we screened the functional SNPs within the intergenic region between Gm40097 and Golph3l (E1383794/enhP region shown in Figure 7d, this region comprises the Klf5 binding motif and is highly conserved between humans and mice). Based on the JASPAR database, three SNPs (rs868751, rs6700022, and rs2275236) were predicted in this conserved region, and they overlap with the binding motif of Klf5. Remarkably, rs6700022 located at –757 bp upstream of Golph3l transcription start site was syntenic to the mouse Gm40097 enhancer locus, implying that rs6700022 might function as an enhancer regulating Golph3l expression by recruiting Klf5 (Figure 7d). Further, we isolated genomic DNA from peripheral blood nucleated cells and then conducted target sequencing for these 3 SNPs in the conserved region. As shown in Figure 7e, genotypes CC or AC at rs6700022 displayed significantly higher Golph3l expression than that of genotype AA, suggesting that the rs6700022 A‐to‐C variant creates a novel binding motif for Klf5, which facilitates enhancer/promoter activity of Golph3l gene. However, rs868751 and rs2275236 SNPs did not significantly affect Golph3l expression (Figure 7e).

In order to further confirm the implication of rs6700022 A‐to‐C transition in the regulation of Golph3l expression, we cloned a 281 bp intergenic region surrounding rs6700022 (with either “A” or “C” allele) into pGL3 luciferase vectors and transfected them into VSMCs to check the effect of allele‐specific variation on the reporter activity. The results showed that luciferase activity had no significant change in cells transfected with the constructs containing the rs6700022 A allele when compared with cells transfected with empty vector; but rs6700022 A‐to‐C transition significantly increased the luciferase activity, indicating a promoting effect of this newly‐formed Klf5 binding site on Golph3l expression (Figure 7f,g). Moreover, ChIP followed by qRT‐PCR in human aortic tissues with different genotypes at rs6700022 also showed that genomic region surrounding rs6700022 C allele was enriched by immunoprecipitation with anti‐Klf5 antibody, but not by rabbit IgG (negative control) (Figure 7h). Together, these findings provide clear evidence that genetic variant of Golph3l rs6700022 A‐to‐C, which creates a novel binding motif for Klf5, is associated with high expression of Golph3l in patients with AD and AAA, and this variant may be useful in predicting the development and progression of vascular remodeling‐related diseases.

To further assess the clinical implications of SNP (rs6700022A>C), 782 individuals (including 637 AD patients with short‐term postoperative survival and 145 AD patients with short‐term postoperative death), whose data were available from Forth Hospital of Hebei Medical University, were randomly divided in a 7:3 ratio into training (*n* = 548) and validation (*n* = 234) cohorts. The baseline characteristics and clinical parameters of AD patients are shown in Table S3 (Supporting Information). We performed a correlation analysis to identify the variables associated with rs6700022A>C. As shown in Figure 7i, heat map exhibited a significant association between rs6700022A>C and postoperative death (*R* = 0.39, *p* < 0.001). Moreover, 14 nonzero coefficient features associated with AD were selected by LASSO regression including: multiple organ injuries, acute kidney injury, bedside blood purification, brain injury, gastrointestinal bleeding, creatine kinase isoenzyme, α‐hydroxybutyric acid, sex, delirium, shock, and rs6700022A>C (Figure 7j). Furthermore, we integrated these features into eXtreme Gradient Boosting (XGBoost) to predict the postoperative short‐term death risk of AD patients, which presented a pretty predictive performance in discovery (AUC = 0.886) and validation (AUC = 0.891) cohorts (Figure 7k), providing a favorable net benefits for clinical decision‐making across most threshold probabilities (Figure 7l). Consequently, SHAP‐based summary plot interpreted the intrinsic information of the XGBoost model including SNP (rs6700022A>C) in predicting the postoperative short‐term death risk (Figure 7m). Finally, we have deployed this model on a dedicated website (https://golph3lpredicted.shinyapps.io/document/), its accessibility and utility were verified by streamlining the input of 14 features from two patients with and without the postoperative short‐term death, respectively (Figure 7n). This user‐friendly platform using genetic and clinical data by machine learning enables clinicians to input a patient's first‐day metrics and promptly drive AD risk stratification, contributing to low mortality effectively.

## 3 Discussion

Novel and important findings of the current study include the following: 1) Golph3l upregulation‐mediated alteration of Golgi morphology and function plays a crucial role in the secretion of TNFα from apoptotic VSMCs, thus identifying a previously unknown function for Golgi apparatus in aortic remodeling and aneurysm development. 2) Klf5 activates transcription of Golph3l gene by recruiting Ythdc1 and p300 to the Golph3l promoter. 3) m6A‐modified Gm40097 mediates Klf5 interaction with Ythdc1 and p300. 4) Rs6700022 A‐to‐C transition in the upstream regulatory region of Golph3l gene, which creates a novel binding motif for Klf5, is associated with high expression of Golph3l in patients with AD and AAA. 5) An XGBoost‐SHAP‐based interpretable predictive website for AD risk stratification was built based on AD patient's features, including this SNP. These findings provide new insights into the critical roles of the Golgi apparatus in aortic dissection and aneurysm development.

AIP is a compensatory mechanism to maintain tissue homeostasis following unexpected cell loss during normal development or under different pathological conditions. This is essential for tissue repair and regeneration and may also be a contributing factor to cancer and drug resistance.^[^ ^45^ ^]^ During AIP, apoptotic cells release mitogenic proteins such as TNF‐α, and the later interacts with receptors on neighboring cells to activate various kinases which promote cell proliferation and survival.^[^ ^46^ ^]^ Actually, AIP pathways involve multiple processes, including apoptosis, the induction of proliferation mediated by caspases, and changes in the mechanisms that involve the JNK and Wnt3a signaling pathways.^[^ ^46^ ^]^ A recent study showed that VSMC apoptosis could also induce both apoptosis or cell proliferation in adjacent live VSMCs by activating multiple caspase‐independent intracellular signaling cascades, leading to release of soluble cytokines involved in the regulation of both cell proliferation and apoptosis. Moreover, induced VSMC apoptosis augmented cell proliferation after vessel injury induced by carotid artery ligation, a model of neointimal hyperplasia.^[^ ^47^ ^]^ Despite these significant progresses, it is unclear whether AIP in VSMCs occurs in the development of aortic aneurysm and dissection, both of which are characterized by VSMC loss resulted from apoptosis. Here, we demonstrated that the percentages of both apoptotic and adjacent proliferating VSMCs were significantly increased in AD and AAA tissues of mice and humans compared with their respective control aortas. Using a Transwell coculture system for AngII‐treated/untreated VSMCs, we found that AngII‐induced apoptotic VSMCs could significantly stimulate the proliferation of VSMCs grown in the different compartments. These observations suggest that the apoptotic VSMCs induced by AngII stimulation are able to stimulate adjacent VSMC proliferation. However, this AIP in VSMCs during development of aortic aneurysms was not sufficient to compensate the loss of VSMCs elicited by apoptosis, as evidenced by fact that the number of apoptotic VSMCs was much higher than that of proliferating cells in AD and AAA tissues, thus contributing to aortic wall thinning and the development of aortic aneurysms. Notably, we observed that AIP in VSMCs during development of AD and AAA was dependent on Golph3l‐mediated Golgi morphology alteration and the release of TNF‐α from apoptotic VSMCs.

The precise morphology and structure of the Golgi are relevant to the regulation of a number of cell processes, such as autophagy, cell migration, signaling, metabolism, and DNA repair,^[^ ^48^, ^49^ ^]^ all of which are involved in cell survival and apoptosis. The changes in Golgi morphology include either fragmentation of the Golgi (loss of Golgi ribbon) or the formation of a very compact and condensed Golgi in the perinuclear location.^[^ ^18^ ^]^ Altered Golgi morphology has been proved to be associated with neurodegenerative diseases, cancer, and some genetic disorders.^[^ ^18^ ^]^ However, the potential involvement of Golgi morphology alteration in aortic aneurysm has not been reported. In the present study, we observed a significantly compacted Golgi structure in VSMCs of AD and AAA tissues of mice and humans. This suggests that alteration of Golgi morphology might be implicated in AIP of VSMCs and aortic aneurysm development. To further explore how Golgi morphology is regulated in AngII‐stimulated VSMCs, we performed RNA‐sequencing on cultured VSMCs treated with or without AngII to search for the proteins that regulate Golgi morphology. As a result, Golph3l, a Golgi membrane protein, was identified as a potential candidate since it is known to be able to regulate Golgi morphology.^[^ ^22^ ^]^ Golph3l and Golph3 are two related proteins that play crucial roles in maintaining the structure of the Golgi apparatus and regulating its functions. Previous studies have shown that Golph3 enhances protein secretion, while Golph3l functions as an antagonist of Golph3.^[^ ^22^ ^]^ However, biological reality is more complex. More recent research suggests that under specific contexts (like cancer), Golph3l and Golph3 can function cooperatively or in parallel to promote similar outcomes.^[^ ^50^, ^51^ ^]^ Therefore, the relationship between these two related proteins appears to be context‐dependent, with evidence for both antagonistic and cooperative functions. In this study, Golph3l gain‐ and loss‐of‐function studies verified that Golph3l depletion in AngII‐treated VSMCs resulted in Golgi dispersal, whereas its overexpression led to Golgi compaction. These changes of Golgi morphology were accompanied by a significant decrease or increase in secretion of TNF‐α in AngII‐stimulated VSMCs. These findings suggest that in the context of AngII‐induced AIP and aneurysm formation, Golph3l and Golph3 function cooperatively or in parallel to enhance Golgi compaction and the protein secretion in AngII‐stimulated VSMCs.

We further investigated the upstream molecular mechanism leading to the activation of the Golph3l promoter in AngII‐stimulated VSMCs. Interestingly, we found that lncRNA Gm40097, a transcript of adjacent gene of Golph3l, was highly increased in AngII‐treated VSMCs and was required for Golph3l expression, as evidenced by loss‐ and gain‐of‐function experiments showing that knockdown of Gm40097 downregulated, whereas its overexpression enhanced, Golph3l expression level. These data are consistent with the notion that neighboring genes are often coordinately coexpressed within *cis*‐regulatory modules and can regulate each other's expression,^[^ ^33^, ^52^ ^]^ suggesting a potential regulation of Gm40097 on its adjacent gene Golph3l. Additionally, we found that treating VSMCs with AngII obviously enhanced Gm40097 m6A modification, as shown by MeRIP‐qPCR, and that the interaction of m6A‐Gm40097 with m6A reader Ythdc1 was essential for Golph3l expression. These findings reveal an important role for m6A‐Gm40097 and Ythdc1 in transcription activation of Golph3l gene. Considering that m6A RNA modification and Ythdc1 exert an essential regulatory effect on chromatin modification and accessibility,^[^ ^34^, ^35^ ^]^ we examined the effect of Ythdc1 depletion in VSMCs on the level of H3K27ac, a major epigenetic mark of active chromatin. Our results showed that Ythdc1 depletion led to a widespread decrease of chromatin accessibility in AngII‐stimulated VSMCs. Because both chromatin accessibility and transcription factor binding are fundamental to activate transcription, we then explored transcription factor(s) that is (are) involved in the activation of Golph3l transcription. The results from ChIP‐PCR assay, CoIP, and gain‐ and loss‐of‐function studies indicate that m6A‐Gm40097‐mediated formation of Ythdc1‐Klf5‐p300 transcription complex on the Golph3l promoter is responsible for the activation of Golph3l transcription. In this transcription complex, Ythdc1 and p300 mediated chromatin remodeling, whereas Klf5 directly bound to the Golph3l promoter to activate its transcription. These findings unveil a novel lncRNA m6A modification‐dependent regulatory mechanism of chromatin remodeling and transcription activation.

In recent years, emerging evidence indicates that super‐enhancer RNAs (seRNAs) are an important layer in transcriptional regulation. Super‐enhancers refer to large clusters of enhancers featured by high levels of transcription factor binding and the extensive intensity of epigenetic markers, such as H3K27ac, H3K4me1, and H3K4me3. The activated super‐enhancers recruit transcription factors and RNA polymerase II (RNAPII) to generate seRNAs.^[^ ^53^, ^54^, ^55^ ^]^ These transcripts from enhancer regions are referred to seRNAs or eRNAs, which are generally nonpolyadenylated and unstable. As an integral component of active enhancers, seRNA transcription generally correlates with enhancer activation and can serve as an independent marker of active enhancers. m6A‐modified seRNAs may affect the chromatin state and the downstream gene transcription through reader protein Ythdc1 recruitment.^[^ ^38^, ^56^ ^]^ In this study, we demonstrated that Gm40097 gene, whose transcript acts as a seRNA, is located upstream of Golph3l gene, and these two genes share the same promoter and were coinduced by AngII. More importantly, MeRIP‐qPCR revealed that m6A‐Gm40097 level was dramatically enhanced in AngII‐treated VSMCs, and AngII treatment facilitated the interaction of m6A‐Gm40097 with Ythdc1, upregulating the Golph3l expression level. Moreover, manipulating Gm40097 expression could affect the enrichment of H3K27ac to the copromoter region of Golph3l and Gm40097. However, how Gm40097 is m6A‐modified and interacts with macromolecules, including transcription factor, epigenetic regulator, DNA, and RNA to regulate Golgi morphology and function are interesting questions that need to be explored in the future.

Genetic background and single nucleotide polymorphisms (SNPs) have been suggested to affect the efficacy and prognosis in diseases, as indicated by different outcome among different ethnicities^[^ ^57^ ^]^ and various features.^[^ ^58^ ^]^ Although our study highlights the clinical utility of integrating genetic and clinical data using machine learning algorithms to raise the ability to predict postoperative short‐term death risk in AD patients, external validation in independent cohorts is essential to confirm the generalizability of this model. Similarly, Shiota et al. found that the SNP models created by machine learning produced excellent prediction of castration resistance and prognosis in advanced prostate cancer.^[^ ^59^ ^]^ However, the sample size of this cohort is relatively small and homogeneous, which may limit the stability, reliability, and generalizability of the model. Future work will focus on validating this model in larger and more diverse cohorts to further enhance its clinical applicability.

There are several limitations that should be noted. First, although our study shows that VSMCs undergoing apoptosis could induce the proliferation of the adjacent live VSMCs in AngII‐ or AngII+BAPN‐induced AAA or AD mouse models, as well as in vitro transwell coculture experiment of AngII‐treated/untreated VSMCs, not all of the AngII‐treated VSMCs underwent apoptosis. We thus cannot exclude the possibility that surviving VSMCs after AngII treatment could also influence adjacent cell proliferation by releasing some cytokines. A previous study showed that VSMC apoptosis induces a number of cytokines including IL‐6 and GM‐CSF (granulocyte‐macrophage colony stimulating factor), leading to apoptosis‐induced proliferation in adjacent live VSMCs.^[^ ^8^ ^]^ In this study, we used mass spectrometry‐based secretome analysis to determine the differentially expressed proteins in serum‐free medium between the AngII‐treated VSMCs and AngII‐untreated VSMCs. The results revealed that top significantly upregulated proteins were related to the TNF‐α signaling. Moreover, neutralizing TNF‐α with anti‐TNF‐α antibody Golimumab can abrogate AngII‐induced proliferation of adjacent cells. These indicate that Ang II‐stimulated VSMCs release TNF‐α to promote proliferation of neighboring cells. Ideally, truly apoptotic cell‐conditioned medium is utilized to investigate AIP and define the specific mediators driving AIP in VSMCs, which will be important future directions for investigation. Second, although we demonstrate that Golph3l upregulation‐mediated alteration of Golgi morphology from the dispersed Golgi to compacted Golgi enhanced the secretion of TNFα from VSMCs, we did not investigate molecular mechanism linking Golph3l upregulation and Golgi morphology alteration. Future exploration of the regulation of Golgi morphology and function during AIP in VSMCs may provide a new avenue for investigating aortic remodeling and aneurysm development.

## 4 Experimental Section

### Human Aorta Tissue Samples and Plasma Samples

Human aortic dissection (AD) (*n* = 71) and abdominal aortic aneurysm (AAA) (*n* = 18), and normal aortic tissues (*n* = 12) were obtained from Fourth Hospital of Hebei Medical University. The inclusion criteria were as follows: 1) patients with AD, or AAA >18 years of age; 2) obvious clinical manifestations of AD, such as sudden onset of severe chest, back, or abdominal pain; and 3) false lumen or free intima detected using computed tomography angiography. Patients with aortic trauma, pseudoaneurysm, or infectious diseases were excluded. Sex‐ and smoking‐matched healthy controls who had no substantial systemic diseases, including ischemic heart disease, cancer, or infectious disease. This study was conducted in accordance with both the Declaration of Helsinki and the International Conference on Harmonization Guidelines for Good Clinical Practice. The study protocols were approved by the ethics committees of Fourth Hospital of Hebei Medical University (2021k7359) and Hebei Medical University (P2022041). The experiments were carried out with the informed consent of the subjects. Aortic tissues and plasma separated by centrifugation immediately were stored at −80 °C before analysis. A brief description of the participants is found in Table S1 (Supporting Information).

### Genotyping of Human Subjects

Genomic DNA of peripheral blood nucleated cells from patients and health control was extracted using the DNeasy blood and tissue kit (Qiagen) by following the manufacturer's instructions. DNA fragments containing three SNPs (rs868751, rs2275236, and rs6700022) were amplified via PCR with 2 pairs of primer, P1‐F: 5′‐TCTAGTCAAGAGTCAGTGCCA‐3′ and P1‐R: 5′‐ACTGACAAACCCATGGATGT‐3′; P2‐F: 5′‐ ACGTAGGCCCTTTTATCGCT‐3′ and P1‐R: 5′‐ CCAAGAAACTTTAAACCTTCGCG‐3′. PCR products were isolated by using gel electrophoresis and purified by using the QIAquick Gel Extraction kit (Qiagen). Obtained PCR amplicons were sequenced via Sanger DNA sequencing.

A larger cohort was established and the included 782 patients with AD were randomly divided into training and testing cohorts at a 7:3 ratio. The clinical information was collected from the patients, including multiple organ injuries (MODS), acute kidney injury (AKI), bedside blood purification (CBP), brain injury (BD), gastrointestinal bleeding (UGIB), creatine kinase isoenzyme (CKMB), α‐hydroxybutyric acid (GHB), sex, delirium, shock, and the rs‐6700022 variant SNP genotyping. All patients’ information is shown in Table S3 (Supporting Information).

### LASSO Regression for Feature Selection

The important feature variables were screened by least absolute shrinkage and selection operator LASSO regression. Among the included variables, in order to avoid the influence of collinearity problems on the model, Lasso regression was used for feature selection during the optimization of the machine learning model. By choosing a lambda value equal to one standard deviation of the minimum lambda, where the error is within one range of the minimum standard error, these variables are most strongly associated with the outcome variable, ensuring model parsimony and mitigating overfitting problems. Continuous variables in these metrics were normalized by min‐max, and variables were selected by LASSO regression (nfold = 20) using the “glmnet” package in R.

### XGBoost Machine Learning (ML) and SHapley Additive exPlanations (SHAP)

The prediction model was based on the important feature variables of XGBoost. The model was built using the “XGBoost” package, using the train function to optimize the parameters in the caret package and output the best parameter configuration. The XGBoost model was built by setting the learning rate eta to 0.1, the maximum depth (max_depth) to 3, and the number of iteration rounds (that is, the number of enhancement rounds) to 100.

“Shapviz” is an R package for interpreting machine learning model predictions that provides a visual interpretation based on SHAP values and explains how much each feature, either positive or negative, contributes to the model's predictions. The feature importance map was used to show the features that had the most significant impact on the model prediction, and the feature importance was ranked according to the average absolute value of SHAP values. Two samples were selected to create a force plot of SHAP values that were used to predict and interpret the one‐sample model.

### Animals

All animal experiments were performed in accordance with the regulations approved by the Laboratory Animal Ethical and Welfare Committee of Hebei Medical University and followed the National Institute of Health guidelines on the care and use of animals (IACUC‐Hebmu‐P2022009). ApoE^−/−^ mice, Tgln‐cre mice, and Golph3l‐flox mice containing loxP sites flanking exons 2 and 3 were purchased from Suzhou Cyagen Biotechnology Co., Ltd. Smooth muscle cell–specific Golph3l knockout (smcGolph3l^−/−^) mice were generated by crossing Golph3l‐flox mice and Tgln‐cre mice (Golph3l‐flox mice served as controls). Mice were kept under a 12‐h light/dark cycle at 23 °C with ad libitum access to food and water.

### Mouse AD and AAA Model^[^ ^60^ ^]^

AD mouse model: 3‐week‐old male C57BL/6J mice were fed water containing 0.25% β‐aminopropionitrile fumarate (BAPN) (Sigma‐Aldrich, USA) for 21 days, and intraperitoneal injection of Ang II (1.0 mg kg^−1^) at 14 d, once a day for 3 consecutive days. Administration of AngII+BAPN leads to a tear in the inner layer of the aortic wall, thus splitting (dissecting) the inner and middle layers of the aortic wall apart and then creating a new, false channel for blood to flow through, which is referred to as AD.

### AAA Mouse Model

Ang II (Abcam) was infused subcutaneously in 8‐week‐old male ApoE^−/−^ mice via osmotic pumps (Alzet) releasing a constant concentration of 1000 ng kg^−1^ min^−1^ for 28 days. Aortic lumen diameters were assessed by ultrasound Doppler imaging at the end of the experiment. AngII infusion results in a localized dilation of the aorta with a diameter at least 50% greater than the normal size of the aorta, which is referred to as AA.

To determine the effect of ASO‐Gm40097 or ASO‐Ctrl on AD formation and progression, mice were intraperitoneally injected with ASO‐Gm40097 solution (5 mg kg^−1^ day^−1^) once daily from the time of Ang II infusion for 28 days; ASO‐Ctr solution was used as the vehicle as previously described.^[^ ^61^ ^]^ Digital photographs of the abdomens were taken to measure the maximum external diameters. The abdominal arteries were harvested for analysis of RNA, morphology, and histology. The hematoxylin and eosin (H&E) as well as masson staining of aortic tissue have been described by Dong et al.^[^ ^62^ ^]^

### Blood Pressure Measurements

Systolic blood pressure was obtained using a MC 4000 blood pressure analysis system for mice (Hatteras Instruments; Cary, NC) using noninvasive tail cuff plethysmography as reported previously.^[^ ^62^ ^]^ Briefly, conscious mice were placed in a restrainer in a prewarmed chamber before blood pressure examination. A pneumatic pulse transducer was placed on the tail of the mice, and the signals from it were collected and analyzed automatically. Each mouse underwent five successive rounds of measurements, and the mean of these values was recorded.

### Echocardiography

The cardiac function of all the mice was assessed by echocardiography using Vevo2100 system (VisualSonics, CA) with a 15‐MHz linear transducer, as the reported previously.^[^ ^62^ ^]^ Mice were trained for 2–3 days before assessment of cardiac function to eliminate the variability of the measurement. All the echocardiography assessment was performed on conscious mice to eliminate the depressant effect of anesthesia on respiration and cardiac function. Diastolic measurements were made at the maximum left ventricle cavity dimension, whereas systolic parameters were measured during maximum anterior motion of the posterior wall.

### Immunofluorescence and TUNEL Staining

Immunofluorescence staining was performed as previously described.^[^ ^63^ ^]^ Primary antibodies include rabbit anti‐Ki‐67 (1:50, 27309‐1‐AP, Proteintech) or anti‐m6A (1:200, 202003, SYSY) and mouse anti‐α‐actin (1:100, GB13044‐50, Servicebio) incubated at 4 °C overnight. Secondary antibodies (1:200; 021516, and 031806, KPL) were incubated at room temperature for 45 min. Terminal deoxynucleotidyl‐transferase‐mediated dUTP nick end labeling (TUNEL) assay kit (Brdu‐red, ab66110) was used to identify apoptosis cells in aorta tissues following the manufacturer's instructions. Image J software was used for analysis of the fluorescence intensity of images from laser confocal microscope (magnification 630).

### Transmission Electron Microscopy

Samples were processed following standard protocol, including dehydration, embedding, and sectioning and then examined and captured under a Hitachi JEM‐1400 transmission electron microscope (Hitachi, Tokyo, Japan) as previously described.^[^ ^62^ ^]^

### Cell Culture, Coculture, Treatments, and Transfection

The primary VSMCs and mouse aortic smooth muscle cell lines (ATCC, No.CRL2797) used in this study were derived from mouse and human's descending thoracic aortic region. Cells were routinely cultured in low‐glucose Dulbecco's modified Eagle's medium (DMEM, Gibco Life Technologies, Rockville, MD) as described previously.^[^ ^62^ ^]^

Plasmid transfections and gene knockdown with antisense oligonucleotide (ASO) or siRNA were carried out with Lipofectamine 2000 (Thermo Fisher Scientific) according to manufacturer's instructions. The expression plasmids of Golph3l and Gm40097 were created by the placement of mouse Golph3l and Gm40097 cDNA into the pcDNA3.1 vector. SiRNAs and ASO targeting mouse Golph3l (si‐Golph3l) and Gm40097 (ASO‐Gm40097), si‐Mettl3 as well as negative controls (si‐Ctr and ASO‐Ctr) were designed and synthesized by GenePharma (Shanghai, China). Twenty hours following transfection, mouse VSMCs were treated with different concentrations of AngII (0, 10^−8^, 10^−7^, 10^−6^ mol L^−1^). Cells were then harvested and lysed for qRT‐PCR and western blotting.

### CRISPR Deletion

To delete enhancer fragment containing Gm40097 (251 nt) in mouse VSMCs, CRISPR‐Cas9 plasmids were used in this study with Cas9‐Gm40097 and Cas9‐Ctr (each), respectively. Vectors were cotransfected into target cells by using Lipofectamine3000 transfection reagent (Invitrogen). After selection with puromycin (10 µg mL^−1^) for 3 days, the remaining cells were cultured for another 3 days. Then RNA was extracted with TRIZOL, and qPCR was performed to detect the expression of Golph3l genes. Guide RNAs (gRNAs) were designed with tools on Zhang Feng's website (http://CRISPR.mit.edu), and construction procedures were followed by Zhang's study.^[^ ^64^ ^]^ sgRNA oligos used as follows: forward: 5´‐AGCCACCGAGGTTCCTTCAT‐3´; reverse:5´‐ ACAGCTGGTCTGTCCAATGA‐3´.

### Quantitative Real‐Time PCR

Total RNA was isolated from VSMCs (6 × 10^5^ cells per well in a 6‐well plate) and mouse aortas using RNeasy Mini kit (Qiagen, Hilden, Germany) according to the manufacturer's instructions. RNA was reverse transcribed into cDNA using RevertAid reverse transcriptase (Thermo Fisher Scientific). Real‐time PCR was performed in an Applied Biosystems (ABI) 7500 FAST Real‐Time PCR System using SYBR Green PCR mixture. Data were normalized to that of GAPDH expression in each sample. Relative fold changes were calculated using the 2^−ΔΔCt^ algorithm.^[^ ^65^ ^]^ The following primers were used for PCR amplification of target genes (forward and reverse): Golph3l, 5′‐CCAAAACGAGAGCGCGAACTGC‐3′ and 5′‐TTCGGTTGGCTCAGTTGCTTT‐3′; Gm40097, 5′‐GAGGCAGGTTTGGGTTCA‐3′ and 5′‐CTTGGGTTTTCATCTTCAGCAG‐3′; β‐actin, 5′‐AAATCGTGCGTGACATCAAAGA‐3′ and 5′‐GGCCATCTCCTGCTCGAA‐3′.

### Western Blot

Lysates from VSMCs or human and mouse aortas were prepared and separated by SDS‐PAGE, transferred to Immobilon P membranes (Millipore), and incubated with primary antibodies against β‐actin (Beyotime, AF0003, 1:2000), Golph3l (Proteintech, 23153‐1‐AP, 1:4000), Ythdc1 (Proteintech, 67911‐1‐AP, 1:5000), Ythdc2 (Proteintech, 27779‐1‐AP, 1:4000), H3K27ac (Invitrogen, PA5‐85524, 1:2000), H3K9me3 (Proteintech, 39766, 1:1000), Klf4 (Abcam, ab151733, 1:1000), p300 (Novus, NB100‐616, 1:500) according to previous report.^[^ ^65^ ^]^ The densitometry of proteins was calculated by ImageJ software.

### 5‐Ethynyl‐2′‐Deoxyuridine (EdU) Incorporation

The proliferation of VSMCs with different treatments was determined using Cell‐Light EdU Apollo488 In Vitro Kit (RiboBio, C10310‐3) according to the manufacturer's instructions. Briefly, 10 nm EdU was added for 12 h staining of VSMCs, followed the cells were washed with PBS, and the cells were fixed in 4% paraformaldehyde and stained according to the manufacturer's instructions. Cell proliferation was observed under a fluorescence microscope (Leica DMI6000B).

### Secretome Assay

Mouse VSMCs reached ≈80% confluence, culture media was replaced with DMEM containing no FBS with or without AngII treatment (100 ng mL^−1^; Abcam) and incubated at 37 °C for 24 h. Culture supernatants were then centrifuged at 800 ×g for 5 min, pooled, and dried using a lyophilizer. Dried samples were homogenized in Tris buffer solution with a protease inhibitor cocktail and phosphatase inhibitor (Sigma‐Aldrich). Homogenates were centrifuged at 13 000 rpm for 10 min at 4 °C. Supernatants were collected, and protein concentrations were determined using a BCA assay (23225; Thermo Fisher Scientific). Proteins were precipitated using 10% trichloroacetic acid at 4 °C overnight. The pellet was washed twice with cold acetone. The protein pellet was solubilized in 100 mm triethylammonium bicarbonate (TEAB).

### Protein Digestion, Peptide Fractionation, and LC‐MS/MS Analysis

Proteins were digested using a filter‐aided sample preparation method.^[^ ^66^ ^]^ Briefly, 50 µg of each sample was reduced by addition of tris (2‐carboxyethyl) phosphine (TCEP) to a final concentration of 10 mm for 30 min at room temperature followed by alkylation with 10 mm iodoacetamide in the dark. Trypsin (Promega) was added to proteins at an enzyme‐to‐protein ratio of 1: 50 w/w. The filter device was placed in a thermomixer and incubated at 37 °C overnight. Second digestion was performed using additional trypsin at 37 °C for 6 h. After collecting the tryptic peptides, the filter was rinsed with 50 mm NH4HCO_3_. The eluent was dried via vacuum centrifugation. Peptide concentration was determined using a BCA assay. The peptide sample was aliquoted into Eppendorf tubes (10 µg per tube), dried using vacuum centrifugation, and stored at ‐80 °C.

To increase proteome coverage, mid‐pH reverse phase liquid chromatography was performed at a flow rate of 0.5 mL min^−1^ using a 130‐min gradient defined by two solvents (10 mm TEAB in water or 90% acetonitrile [pH 7.4]). Through a gradient elution condition, 24 fractions were collected and dried in a vacuum centrifuge concentrator and stored at ‐80 °C.

LC‐MS/MS analysis of peptides was conducted at iProteome Biotechnology Co., Ltd. (Shanghai). For each MS/MS dataset, postexperiment monoisotopic mass refinement was applied to accurately assign precursor masses to MS/MS spectra. Resulting MS/MS spectra were subjected to a database search using the UniProt Mouse reference database. Peptide spectrum matches were obtained using an FDR of 1%.

### RNA‐Seq

RNA‐seq analyses were conducted by Shanghai aksomics Biotechnology Co., LTD, Shanghai, China. RNA extraction of AngII‐treated and ‐untreated VSMCs was performed and sequenced on an Illumina HiSeq 2000 as previously described.^[^ ^62^ ^]^ Illumina sequencing libraries were prepared according to the TruSeq RNA Sample Preparation Guide following the manufacturer's instructions. Libraries were sequenced using 1 × 58 bp single‐end reads, with two indexed samples per lane, yielding about 32.5 million reads per sample. Genes in samples have values greater than or equal to lower cutoff: 100.0 (“All Targets Value”) were chosen for data analysis. GO, GSEA, KEGG Analysis was applied to determine the roles of these differentially expressed genes.

### RNA‐seq Analysis (Public Database)

RNA‐seq data from GEO dataset GSE140947 were normalized and analyzed in human samples (normal *n* = 12 and AAA *n* = 12). Raw data underwent log2 transformation, and the microarray data were normalized by the normalize quantiles function of the preprocessCore R package (v3.4.1). Differential expression, identified using the limma R package, required an adjusted *p*‐value < 0.05 and an absolute log2 (Fold Change)>1, visualized using violin plot from the R package. Compared to Con group, *p < 0.05 was considered statistically significant.

### Methylated RNA Immunoprecipitation Sequencing (MeRIP‐seq)

MeRIP‐seq analyses were conducted by Shu Pu Biotechnologies LLC (Aksomics, Shanghai, China). Total RNA was isolated as before, and purified RNA fragments were isolated using the Arraystar Seq‐Star TM poly(A) mRNA Isolation Kit. The fragmented RNA was sorted into two portions. First, immunomagnetic beads with premixed m6A antibody were added to enrich the mRNA fragments containing m6A methylation. The other was used as a control to construct a conventional transcriptome sequencing library. Library preparation and high‐throughput sequencing were performed by the Illumina Nova seq 6000 platform of LCBIO Biotech (Hangzhou, China). Then, the analysis results of enrichment of m6A peaks and location results were converted into bw files and imported into the Integrative Genomics Viewer (IGV) software (Los Angeles, CA) for visual display. According to the position of RNA modification sites of m6A and total reads number in the bw file, the number of reads obtained from the immunoprecipitation (IP) library and Input library was used to quantify the degree of m6A modification. This data was reflected in the coordinate interval of each modification to form a visual representation in IGV software.

### M6A‐RNA Immunoprecipitation (MeRIP) Assay and qRT‐PCR

MeRIP‐qPCR analysis was performed as previously described.^[^ ^62^ ^]^ Briefly, the total RNAs were extracted from treating VSMCs (ThermoFisher). The RNA concentration was adjusted to 1 µg µL^−1^. RNA was fragmented into ≈100 nt size and these RNAs were immunoprecipitated with the anti‐m6A antibody according to the standard protocol of the Magna MeRIP m6A Kit (Merck Millipore). M6A enrichment was determined by qPCR analysis. Primers targeting m6A enriched regions of Golph3l (six sites) are available in following. Site 1‐F: 5′‐gtggctgcgaggaaaaggct‐3′; Site 1‐R: 5′‐ catcttcagcagtagttgt‐3′; Site 2‐F:5′‐tctctgcaacgaaggatca‐3′; Site 2‐R: 5′‐gctgcagtgggacgtagg‐3′; Site 3‐F:5′‐atttttcacatctcttta‐3′; Site 3‐R: 5′‐ cttttagccccaggagcaata‐3′; Site 4‐F:5′‐ggctacacatctttctgg‐3′; Site 4‐R: 5′‐ctttctgtctaggagtc‐3′; Site 5‐F:5′‐gacagcccaacaggtgat‐3′; Site 5‐R: 5′‐gtagctcgatccacgtctg‐3′; Site 6‐F:5′‐aaccctttcaaattacagta‐3′; Site 6‐R: 5′‐ tcatttacccaccgttctag‐3′. All data were analyzed by adopting 2^−ΔΔCt^ methods.

### Enzyme‐Linked Immunosorbent Assay (ELISA)

TNF‐α and TNFSF12 concentrations in cell culture supernatants were measured using ELISA kits (R&D systems) according to the manufacturer's instructions.

### ChIP‐PCR/qPCR

Treated mouse VSMCs were cross‐linked with 1% formaldehyde for 15 min, lysed as previously described, and then sonicated to an average size of 400–600 bp.^[^ ^66^ ^]^ BeyoChIP Enzymatic Chromatin Immunoprecision (ChIP) Assay Kit with Protein A/G Magnetic Beads (Beyotime, P2083S), Klf5 Polyclonal Antibody (GTX103289, GeneTex), H3K27ac (Invitrogen, PA5‐85524), p300 (Novus, NB100‐616), and Rabbit IgG (Beyotime, A7016) were used for ChIP. DNA purification was performed using PCR/DNA purification kit (Beyotime, D0033). The obtained ChIP‐DNA was subjected to PCR and qPCR detection using Golph3l promoter region primers. PCR products were separated by 1.5% w/v agarose gel electrophoresis. Experiments were repeated three times.

### Chromosome Conformation Capture qPCR (3C‐qPCR) Analysis

3C‐qPCR was performed following standard protocols.^[^ ^67^ ^]^ Briefly, 1x10^6^ AngII‐stimulated VSMCs with and without ML264 inhibitor for 24 h were cross‐linked with equal volume of 4% formaldehyde and quenched with 1 m glycine (cold). Next, the samples were lysed and nuclei were digested with EcoRI at 37 °C overnight, followed by ligation and subsequent DNA purification and DNA precipitate. The purpose of 3C analysis was to assess the physical interactions between Klf5/p300/H3K27ac and target regions. To examine the interactions between Klf5 or p300 and assessed region, two primers were designed (reverse and forward) according to sequence between Golph3l and Gm40097 (GeneID: 132409621). The following primers were used for quantitative PCR (qPCR). 3C primers including

3C‐1(Mus musculus) Forward Primer: 5′‐tgccaacatttcttaac‐3′;
3C‐2(Mus musculus) Reverse Primer: 5′‐tgctgccaatcacgaaagga‐3′;
3C‐3(Mus musculus) Forward Primer: 5′‐tgggtaatttcatcggggtg‐3′;
3C‐4(Mus musculus) Reverse Primer: 5′‐ggagcttacgaggggaggtc‐3′.

### Luciferase Reporter Assays

Mouse VSMCs (3 × 10^4^ VSMCs were seeded into each well) were cotransfected with reporter plasmids and the control pTK‐RL plasmid using Lipofectamine 2000 reagent (Invitrogen) as previously described.^[^ ^62^ ^]^ Luciferase assays were performed after 24 h using a dual luciferase assay kit (Promega). Specific promoter activity was expressed as the relative ratio of firefly luciferase activity to Renilla luciferase activity. All promoter constructs were evaluated in a minimum of three separate wells per experiment.

### N6‐Methyladenosine (m6A) Dot Blot Assay

Total RNA was isolated as described above. RNA samples dissolved in 3 times volume of RNA incubation buffer were denatured at 65 °C within 5 min. Then the samples, divided into subgroups of 200 ng, were loaded to an Amersham Hybond‐N+ membrane (GE Healthcare, USA) installed in a Bio‐Dot Apparatus (Bio‐Rad, USA) with the mixture of ice‐cold 20*SSC buffer (Sigma‐Aldrich, Germany). The membrane was UV crosslinked for 5 min and washed with PBST. Whereafter, 0.02% Methylene blue (Sangon Biotech, China) staining followed by the scanning to indicate the total content of input RNA. After being blocked with 5% nonfat milk, the membrane was incubated with specific m6A antibody (1:1000, Millipore) overnight at 4 °C. Dot blots were hatched with HRP‐conjugated antimouse immunoglobulin G (IgG) for 1 h before visualized by an imaging system (Bio‐Rad, USA).

### Cell Immunofluorescence

Immunofluorescence staining was performed as described previously.^[^ ^62^ ^]^ Cells were permeabilized with 0.1% Triton X‐100 in PBS and then blocked with 0.1% Triton X‐100 and 5% BSA in PBS for 1 h, washed, and incubated overnight with the primary antibodies anti‐Golph3l (1:100, 23153‐1‐AP, Proteintech), anti‐GM130 (1:100, 66662‐1‐Ig, Proteintech) at 4 °C. Antibodies conjugated to Alexa Fluor 405, 488, and 568 were used as secondary antibodies. Nuclei were stained with DAPI. All images of primary cells were taken with 63 × NA 0.75 objectives by confocal microscopy (DM6000 CFS, Leica). Fifty cells were counted for each assay.

### Immunofluorescence In Situ Hybridization (ImFISH)

The treated VSMCs were subjected to imFISH analysis using Gm40097 RNA probe (red) (Abbott Molecular Diagnostics, Des Plaines, IL). The immunofluorescence assay was carried out with primary antibodies anti‐Ythdc1 (green) or anti‐Klf5 (blue) and anti‐p300 (green). The glass slides were washed three times with Tris‐buffered saline before mounting. Cell nuclei were stained with 4′,6‐diamidino‐2‐phenylindole (DAPI) dry, and then visualized under a fluorescence microscope.

### RNA‐Pulldown Assays

Biotin‐labeled oligonucleotide probe of Gm40097 was commercially synthesized (RiboBio, China). Briefly, biotin‐labeled oligonucleotide probes were incubated with BeyoMag streptavidin magnetic beads (Beyotime; P2151) for 60 min at room temperature. After being bound to streptavidin magnetic beads, the probe‐beads were incubated with whole cell lysates from AngII‐treated mouse VSMCs overnight at 4 °C. After washing with ice‐cold PBS three times, proteins pulled down by the probed‐coated beads. Subsequently, the eluted proteins were analyzed by Western blotting assay using anti‐Ythdc1 antibody or LC‐MS/MS (Shanghai Bioprofle). For mass spectrometry‐based proteomic analysis, gel pieces were dehydrated with acetonitrile and digested with trypsin. A validated liquid chromatography tandem‐mass spectrometry (LC‐MS/MS) analysis of peptides was conducted at iProteome Biotechnology Co., Ltd. (Shanghai).

### RNA Immunoprecipitation (RIP) Assays

The RIP experiment was performed using the Magna RIP RNA binding protein immunoprecipitation kit (CAT.17‐701, Millipore, Billerica, MA), and all operations were performed following the manufacturer's instructions. The antibody against Ythdc1 for the RIP assay was purchased from Proteintech (67911‐1‐AP). Experiments were repeated three times.

### Single Cell RNA‐seq

Single cell RNA‐seq analyses were conducted by YuceBio Technology Co., Ltd, Shenzhen China. Sc‐RNA seq was performed using the 10× Genomics’ single cell solution. The harvested AD (*n* = 3) and control (*n* = 3) mouse aortas was washed three times with cold PBS. Single‐cell suspensions from 3 aortas in control group were pooled together as one sample and three aortas in experimental group were pooled together as another sample respectively. The emulsion and the library were prepared according to the user guide of 10× Genomics Chromium Single Cell 3′v3 Reagent Kit. GEMs (Gel Bead‐inEmulsions) are generated by combining specimens, a Master Mix, gel beads and partitioning oil, onto the chip in the chromium single cell controller kit. After the cell is lysed and RNA reverse transcription occurs, the cDNA was purified from the mixture. The cDNA was amplified, fragmented, and spliced to generate sufficient mass for library construction. The cDNA was subsequently sequenced by Illumina sequencer.

### Analysis of Single‐Cell RNA Sequencing Data

A criterion to filter out cells with UMI/gene numbers out of the limit of mean ± 2 fold of standard deviations was applied to remove cells of low quality and likely multiple captures, which is a major concern in microdroplet‐based experiments. Low‐quality cells where >10% of the counts belonged to mitochondrial genes were further discarded following visual inspection of the distribution of cells by the fraction of mitochondrial genes expressed. All primary data of scRNA sequencing are submitted to NCBI, and the GEO accession number is GSE303634.

### Assay for Transposase‐Accessible Chromatin (ATAC)‐Sequencing and Data Processing

ATAC‐seq analyses were conducted in duplicate by Guangzhou Epibiotek Co., Guangzhou, China. The ATAC‐seq protocol was performed in the si‐NS (*n* = 3) and si‐Ythdc1 (*n* = 3) pretransfected mouse VSMCs under AngII treatment (10^−7^ mol L^−1^). 50 000 cells were pretreated with DNase for 30 min at 37 °C to remove free‐floating DNA by centrifugation and to digest DNA from dead cells. Resuspend the cell pellet in 50 µL cold lysis buffer and incubated on ice for 3 min, followed by addition of 1 mL of ATAC‐seq RSB. Nuclei were then centrifuged and resuspended in 50 µL of transposition mix (10 µL 5×TD buffer, 5 µL Tn5 transposase, 35 µL water). Transposition reactions were incubated at 37 °C for 30 min in a thermomixer with shaking. Reactions were cleaned up with Zymo DNA Clean and Concentrator 5 columns. After PCR and size selection, DNA were resuspended in 25 µL of 2 × HiFi PCR mix, 1 µL of each Nextera i5 primer and Nextera i7 primer. The following PCR program was performed: 72 °C for 5 min, 98 °C for 1 min, 14 cycles; and then 98 °C for 15 s, 60 °C for 30 s, and 72 °C for 1 min. After PCR, Size selection with the DNA Clean Beads, 35 µL of DNA Clean Beads were added, Supernatant was transferred to a new tube, and 10 µL of fresh beads was added to capture fragments range from 250 to 350 bp. Libraries were quantified with Bioptic Qsep100 Analyzer (Bioptic lnc.) and paired‐end sequenced with read lengths of 150 bp.

The ENCODE ATAC‐seq pipeline was used for quality control and statistical signal processing of short‐read sequencing data, producing alignments and measures of enrichment. 150 bp paired‐end reads were mapped to the reference genome build (human, hg38; mouse, mm10). Differentially sites were detected from ATAC‐seq experiments using DiffBind R package. All primary data of ATAC sequencing are submitted to NCBI, and the GEO accession number is GSE309196.

### HiChIP‐seq and Data Analysis

HiChIP‐seq analyses were conducted in duplicate by Guangzhou Epibiotek Co., Guangzhou, China. The HiChIP‐seq protocol was conducted and H3K27ac (Invitrogen, PA5‐85524, 9 µL per ChIP) antibody was used in vehicle control and Klf5 inhibitor ML264‐pretreated mouse VSMCs under AngII treatment (10^−7^ mol L^−1^) as described previously.^[^ ^68^ ^]^ Briefly, cells were cross‐linked in 1.5% formaldehyde and were pelleted, washed and centrifugated. Capture HiChIP processing FASTQ files (paired‐end Illumina data) were processed with the HiC‐Pro v2.11.1 using Bowtie2 v2.3.5.1 for mapping short reads to the reference genome mm10/hg19. After the mapping, filtering and deduplication steps of the HiC‐Pro pipeline, normalized matrices were generated at 500 or 10 kb resolutions. A large number of loop structures can be identified by Fit HiChIP analysis software (https://www.nature.com/articles/s41467‐019‐11950‐y). Jointly models the nonuniform coverage and genomic distance scaling of contact counts to compute statistical significance estimates. In order to show the specific loop structure, Enhance/Superenhancer of the database was needed to combine to peaks quantification of loop region. HiC Plotter can draw maps of all chromosomes with different resolutions. The “all.validpair” files generated after HiC‐Pro processing were converted into “hic” file, which can be visualized by juicebox. All data of HiChIP sequencing are submitted to NCBI, and the GEO accession number is GSE309513.

### Global Run‐On Sequencing (GRO‐seq) and Data Analysis

GRO‐seq analyses were conducted in duplicate by Guangzhou Epibiotek Co., Guangzhou, China. Mouse VSMCs were subjected to above ML264 and AngII treatment. The GRO‐seq protocol was adapted in nuclei extraction, workflow process of GRO‐seq single‐end library, and UMI‐like randomers that are part of the custom GRO‐seq protocol.^[^ ^69^ ^]^ The Adapter trimming was performed using Cutadapt with the parameter “‐a AACTGTAGGCACCATCAAT”. The trimmed reads were mapped to the reference genome build (mm10/hg19) by using BWA software. HOMER software suite was used to create a tag directory from the GRO‐seq experiment. These results include GC content, the nucleotide frequencies, nucleotide preferences, and correlation data. Subsequently, the annotate Peaks.pl program was used to annotate peaks and output a histogram text file of GRO‐seq reads near the TSS. A histogram chart was created by graphing the 3rd and 4th columns (that separate reads by strand) with ggplot2 R‐package. NRSA (Nascent RNA Sequencing Analysis) tool was employed to quantify nascent transcription for known genes, and detect, annotate, and quantify active enhancers.

### ChIP Sequencing Data Acquisition

In this study, the high‐throughput data were obtained from GEO datasets: Klf5 ChIP‐seq data were from GSE80812,^[^ ^70^ ^]^ Histone H3 Lysine 27 acetylation (H3K27Ac) data was from GSE145964.^[^ ^71^ ^]^

### Statistical Analysis

Statistical analysis was performed with GraphPad Prism 10.1.2 (GraphPad Inc., USA). For all experiments, power analyses based on preliminary data were conducted, and selected cohort sizes were sufficient to give a power of 0.8 at an alpha of 0.05. All analyses were performed in a blinded manner. The mice were randomized before assignment to different groups. No outliers were excluded. The data were tested for normality before parametric statistics with the Shapiro–Wilk normality test. For comparisons between 2 groups, the unpaired Student *t‐*test was applied for normally distributed data, and the Mann–Whitney test was applied for non‐normally distributed data. For comparisons among ≥3 groups, 1‐way or 2‐way ANOVA was followed by the Tukey multiple‐comparisons test. Correlation analysis between 2 numeric variables was performed with the nonparametric Spearman rank correlation test. Data are presented as mean ± standard deviation (SD) or mean ± standard error (SE).

With the occurrence of postoperative death as the outcome variable, the important feature variables were screened by LASSO regression, and the prediction model was based on the extreme gradient boosting algorithm (XGBoost). The area under the receiver operating characteristic curve (AUC), accuracy and F1 score were used to compare the prediction performance of different models. Decision curve analysis (DCA) was used to evaluate the clinical effectiveness of the model. The xgb.plot.importance function in RStudio was used to show the variable importance ranking to further SHAP interpretation of the XGBoost model. In this study, “tidyverse,” “pROC,” “CBCgrps,” “rms,” and “rmda” packages were used for data collation and visualization, and *p* < 0.05 was considered statistically significant. The Shiny package was used to deploy the XGBoost model into shinyapps.io to develop an online prediction calculator for postoperative mortality risk of aortic dissection.

## Conflict of Interest

The authors declare no conflict of interest.

## Author Contributions

W.‐l.W., Z.‐x.S., and S.‐m.B. contributed equally to this work. W.W. and D.M. conceived, designed, and supervised the project; W.W., Z.S., Y.L., Q.L., X.M., X.Z., L.Z., B.Z., X.Z., H.Z., and S.B. conducted experiments and analyzed data; W.W., Z.S., D.M., J.W., L.Z., and S.B. provided reagents; Y.Z., X.M., D.B. W.Z., X.S., and Y.X. collected human AD and AAA samples; D.M. provided the schematic; W.W., D.M., and J.W. wrote the manuscript; W.W., D.M., Z.S., S.B., and J.W. conducted review & editing; D.M. and J.W. conducted funding acquisition. All authors have read and approved the article.

## Supporting information



Supporting Information

Supporting Information

## Data Availability

The data that support the findings of this study are available from the corresponding author upon reasonable request.

## References

[1] J. Raffort , F. Lareyre , M. Clément , R. Hassen‐Khodja , G. Chinetti , Z. Mallat , Nat. Rev. Cardiol. 2017, 14, 457. 28406184 10.1038/nrcardio.2017.52

[2] K. B. Rombouts , T. A. R. van Merrienboer , J. C. F. Ket , N. Bogunovic , J. van der Velden , K. K. Yeung , Eur. J. Clin. Invest. 2022, 52, 4. 10.1111/eci.13697 PMC9285394 34698377

[3] J. Gao , H. Cao , G. Hu , Y. Wu , Y. Xu , H. Cui , H. S. Lu , L. Zheng , Signal Transduct. Target Ther. 2023, 8, 155. 36737432 10.1038/s41392-023-01325-7 PMC9898314

[4] R. A. Quintana , W. R. Taylor , Circ. Res. 2019, 124, 607. 30763207 10.1161/CIRCRESAHA.118.313187 PMC6383789

[5] G. Q. Qian , O. Adeyanju , A. Olajuyin , X. Guo , Life 2022, 191, 12. 10.3390/life12020191 PMC8880357 35207478

[6] Y. Fan , S. Wang , J. Hernandez , V. B. Yenigun , G. Hertlein , C. E. Fogarty , J. L. Lindblad , A. Bergmann , PLoS Genet. 2014, 10, 1004131. 10.1371/journal.pgen.1004131 PMC3907308 24497843

[7] N. Diwanji , A. Bergmann , Fly 2017, 11, 46. 27575697 10.1080/19336934.2016.1222997 PMC5354222

[8] D. Aravani , K. Foote , N. Figg , A. Finigan , A. Uryga , M. Clarke , M. Bennett , Apoptosis 2020, 25, 648. 32627119 10.1007/s10495-020-01622-4 PMC7527356

[9] Z. Xie , J. Chen , C. Wang , J. Zhang , Y. Wu , X. Yan , J. Mol. Cell. Biol. 2021, 13, 79. 33493334 10.1093/jmcb/mjaa080 PMC8104942

[10] D. Palioura , A. Lazou , K. Drosatos , J. Mol. Cell. Cardiol. 2022, 163, 56. 34653523 10.1016/j.yjmcc.2021.10.002 PMC8816822

[11] T. Shindo , I. Manabe , Y. Fukushima , K. Tobe , K. Aizawa , S. Miyamoto , K. Kawai‐Kowase , N. Moriyama , Y. Imai , H. Kawakami , H. Nishimatsu , T. Ishikawa , T. Suzuki , H. Morita , K. Maemura , M. Sata , Y. Hirata , M. Komukai , H. Kagechika , T. Kadowaki , M. Kurabayashi , R. Nagai , Nat. Med. 2002, 8, 856. 12101409 10.1038/nm738

[12] D. Ma , B. Zheng , T. Suzuki , R. Zhang , C. Jiang , D. Bai , W. Yin , Z. Yang , X. Zhang , L. Hou , H. Zhan , J.‐K. Wen , Circ. Res. 2017, 120, 799. 28115390 10.1161/CIRCRESAHA.116.310367

[13] D. Ma , B. Zheng , H.‐L. Liu , Y.‐B. Zhao , X. Liu , X.‐H. Zhang , Q. Li , W.‐B. Shi , T. Suzuki , J.‐K. Wen , PLoS Biol. 2020, 18, 3000808. 10.1371/journal.pbio.3000808 PMC7462304 32817651

[14] X. Li , Y. He , Y. Xu , X. Huang , J. Liu , M. Xie , X. Liu , Am. J. Physiol. Lung Cell Mol. Physiol. 2016, 310, L299. 26702149 10.1152/ajplung.00189.2015

[15] A. Courboulin , V. L. Tremblay , M. Barrier , J. Meloche , M. H. Jacob , M. Chapolard , M. Bisserier , R. Paulin , C. Lambert , S. Provencher , S. Bonnet , Respir. Res. 2011, 128, 12. 10.1186/1465-9921-12-128 PMC3193170 21951574

[16] L. F. S. Mendes , A. F. Garcia , P. S. Kumagai , F. R. de Morais , F. A. Melo , L. Kmetzsch , M. H. Vainstein , M. L. Rodrigues , A. J. Costa‐Filho , Sci. Rep. 2016, 6, 29976. 27436376 10.1038/srep29976 PMC4951691

[17] J.‐H. Wei , J. Seemann , Curr. Opin. Cell Biol. 2017, 47, 43. 28390244 10.1016/j.ceb.2017.03.008 PMC5537018

[18] C. Makhoul , P. Gosavi , P. A. Gleeson , Front. Cell. Dev. Biol. 2019, 7, 112. 31334231 10.3389/fcell.2019.00112 PMC6616279

[19] C. S. Wijaya , S. Xu , Cell Regen. 2024, 4, 13. 10.1186/s13619-024-00187-w PMC10864233 38349608

[20] Q. He , H. Liu , S. Deng , X. Chen , D. Li , X. Jiang , W. Zeng , W. Lu , Front. Cell Dev. Biol. 2020, 8, 830. 33015040 10.3389/fcell.2020.00830 PMC7493689

[21] M. D. Buschman , M. Xing , S. J. Field , Front. Neurosci. 2015, 9, 362. 26500484 10.3389/fnins.2015.00362 PMC4595774

[22] M. M. Ng , H. C. Dippold , M. D. Buschman , C. J. Noakes , S. J. Field , Mol. Biol. Cell 2013, 6, 796. 10.1091/mbc.E12-07-0525 PMC3596250 23345592

[23] H. D. Ryoo , A. Bergmann , Harb. Perspect. Biol. 2012, 4, a008797. 10.1101/cshperspect.a008797 PMC3405855 22855725

[24] S. Das , E. Zhang , P. Senapati , V. Amaram , M. A. Reddy , K. Stapleton , A. Leung , L. Lanting , M. Wang , Z. Chen , M. Kato , H. J. Oh , Q. Guo , X. Zhang , B. Zhang , H. Zhang , Q. Zhao , W. Wang , Y. Wu , R. Natarajan , Circ. Res. 2018, 123, 1298. 30566058 10.1161/CIRCRESAHA.118.313207 PMC6309807

[25] H. Quelquejay , R. Al‐Rifai , M. Silvestro , M. Vandestienne , I. Ferreira , T. Mirault , D. Henrion , X. Zhong , I. Santos‐Zas , G. Goudot , P. Alayrac , E. Robidel , G. Autret , D. Balvay , S. Taleb , A. Tedgui , C. M. Boulanger , A. Zernecke , A.‐E. Saliba , J. Hadchouel , B. Ramkhelawon , C. Cochain , S. Bergaya , X. Jeunemaitre , H. Ait‐Oufella , Circ. Res. 2024, 135, 488. 38979610 10.1161/CIRCRESAHA.124.324366

[26] K. Park , S. Ju , N. Kim , S.‐Y. Park , BMB Rep. 2021, 54, 246. 33612152 10.5483/BMBRep.2021.54.5.270 PMC8167249

[27] J. Li , E. Ahat , Y. Wang , Res. Probl. Cell Differ. 2019, 67, 441. 10.1007/978-3-030-23173-6_19 PMC7076563 31435807

[28] X. Li , J. Yu , L. Gong , Y. Zhang , S. Dong , J. Shi , C. Li , Y. Li , Y. Zhang , H. Li , Free Radic. Biol. Med. 2021, 165, 243. 33493554 10.1016/j.freeradbiomed.2021.01.028 PMC7825924

[29] P. Liu , A. Zhang , Z. Ding , D. Dai , B. Li , S.‐F. Liu , J. Xu , Z. Cheng , S. Zhao , X. Zhao , J. Dong , Am. J. Respir. Cell Mol. Biol. 2022, 67, 574. 35972996 10.1165/rcmb.2021-0429OC

[30] Y.‐S. Chen , X.‐P. Ouyang , X.‐H. Yu , P. Novák , L. Zhou , P.‐P. He , K. Yin , J. Cardiovasc. Transl. Res. 2021, 14, 857. 33630241 10.1007/s12265-021-10108-w

[31] N. Altan‐Bonnet , Methods Mol. Biol. 2007, 390, 309. 17951697 10.1007/978-1-59745-466-7_21

[32] D. G. Lemay , W. F. Martin , A. S. Hinrichs , M. Rijnkels , J. B. German , I. Korf , K. S. Pollard , BMC Bioinform. 2012, 13, 253. 10.1186/1471-2105-13-253 PMC3575404 23020263

[33] S. Li , R.‐C. He , S.‐G. Wu , Y. Song , K.‐L. Zhang , M.‐L. Tang , Y.‐R. Bei , T. Zhang , J.‐B. Lu , X. Ma , M. Jiang , L.‐J. Qin , Y. Xu , X.‐H. Dong , J. Wu , X. Dai , Y.‐W. Hu , Circ. Res. 2024, 134, 60. 38084631 10.1161/CIRCRESAHA.122.322360

[34] X. Zong , L. Huang , V. Tripathi , R. Peralta , S. M. Freier , S. Guo , K. V. Prasanth , Methods Mol. Biol. 2015, 1262, 321. 25555591 10.1007/978-1-4939-2253-6_20

[35] Y. Yang , P. J. Hsu , Y.‐S. Chen , Y.‐G. Yang , Cell Res. 2018, 28, 616. 29789545 10.1038/s41422-018-0040-8 PMC5993786

[36] Y. Xiao , Y. Wang , Q. Tang , L. Wei , X. Zhang , G. Jia , Angew. Chem., Int. Ed. Eng. 2018, 57, 15995. 10.1002/anie.201807942 30345651

[37] J. Liu , X. Dou , C. Chen , C. Chen , C. Liu , M. M. Xu , S. Zhao , B. Shen , Y. Gao , D. Han , C. He , Science 2020, 367, 580. 31949099 10.1126/science.aay6018 PMC7213019

[38] J. Liu , M. Gao , J. He , K. Wu , S. Lin , L. Jin , Y. Chen , H. Liu , J. Shi , X. Wang , L. Chang , Y. Lin , Y.‐L. Zhao , X. Zhang , M. Zhang , G.‐Z. Luo , G. Wu , D. Pei , J. Wang , X. Bao , J. Chen , Nature 2021, 591, 322. 33658714 10.1038/s41586-021-03313-9

[39] S. Miyamoto , T. Suzuki , S. Muto , K. Aizawa , A. Kimura , Y. Mizuno , T. Nagino , Y. Imai , N. Adachi , M. Horikoshi , R. Nagai , Mol. Cell. Biol. 2003, 23, 8528. 14612398 10.1128/MCB.23.23.8528-8541.2003 PMC262669

[40] T. Matsumura , T. Suzuki , K. Aizawa , Y. Munemasa , S. Muto , M. Horikoshi , R. Nagai , J. Biol. Chem. 2005, 280, 12123. 15668237 10.1074/jbc.M410578200

[41] B. Zheng , C.‐Y. Zheng , Y. Zhang , W.‐N. Yin , Y.‐H. Li , C. Liu , X.‐H. Zhang , C.‐J. Nie , H. Zhang , W. Jiang , S.‐F. Liu , J.‐K. Wen , Biochim. Biophys. Acta Mol. Basis Dis. 2018, 1864, 374. 29074464 10.1016/j.bbadis.2017.10.021

[42] L. S. Qi , M. H. Larson , L. A. Gilbert , J. A. Doudna , J. S. Weissman , A. P. Arkin , W. A. Lim , Cell 2013, 152, 1173. 23452860 10.1016/j.cell.2013.02.022 PMC3664290

[43] Y. Dai , F. Xie , Y. Chen , FEBS Open Bio. 2023, 10, 1348. 10.1002/2211-5463.12869 PMC7327903 32343879

[44] I. Ovcharenko , M. A. Nobrega , G. G. Loots , L. Stubbs , Nucl. Acids Res. 2024, 32, W280. 10.1093/nar/gkh355 PMC441493 15215395

[45] C. E. Fogarty , N. Diwanji , J. L. Lindblad , M. Tare , A. Amcheslavsky , K. Makhijani , K. Brückner , Y. Fan , A. Bergmann , Curr. Biol. 2016, 26, 575. 26898463 10.1016/j.cub.2015.12.064 PMC4765900

[46] U. Moreno‐Celis , T. García‐Gasca , C. Mejía , in Metastasis, Vol. 11, (ed: C. M. Sergi ), National Library of Medicine, Rockville Pike Bethesda, MD 2022, p. 35679456.

[47] K. Rajagopalan , J. D. Selvan Christyraj , K. S. Chelladurai , K. Kalimuthu , P. Das , M. Chandrasekar , N. Balamurugan , K. Murugan , Apoptosis 2024, 29, 1399. 38581530 10.1007/s10495-024-01958-1

[48] C. Makhoul , P. Gosavi , P. A. Gleeson , Biochem. Soc. Trans. 2018, 46, 1063. 30242119 10.1042/BST20180323

[49] C. Makhoul , P. Gosavi , R. Duffield , B. Delbridge , N. A. Williamson , P. A. Gleeson , Mol. Biol. Cell 2019, 30, 370. 30540523 10.1091/mbc.E18-05-0313 PMC6589577

[50] B. Baranowski , K. Pawłowski , PeerJ 2023, 11, 15715. 10.7717/peerj.15715 PMC10364804 37492397

[51] R. Li , H. Zhao , X. Huang , J. Zhang , R. Bai , L. Zhuang , S. Wen , S. Wu , Q. Zhou , M. Li , L. Zeng , S. Zhang , S. Deng , J. Su , Z. Zuo , R. Chen , D. Lin , J. Zheng , Nat. Genet. 2023, 55, 2224. 37957340 10.1038/s41588-023-01568-8

[52] R. Mao , Y. Wu , Y. Ming , Y. Xu , S. Wang , X. Chen , X. Wang , Y. Fan , Sci. China Life Sci. 2019, 62, 905. 30593613 10.1007/s11427-017-9370-9

[53] Y. Zhao , J. Zhou , L. He , Y. Li , J. Yuan , K. Sun , X. Chen , X. Bao , M. A. Esteban , H. Sun , H. Wang , Nat. Commun. 2019, 10, 5787. 31857580 10.1038/s41467-019-13598-0 PMC6923398

[54] Q. Chen , Y. Zeng , J. Kang , M. Hu , N. Li , K. Sun , Y. Zhao , Front. Cell Dev. Biol. 2023, 11, 205540. 10.3389/fcell.2023.1205540 PMC10229774 37266452

[55] T. Fukagai , T. S. Namiki , R. G. Carlile , H. Yoshida , M. Namiki , BJU Int. 2006, 97, 1190. 16686710 10.1111/j.1464-410X.2006.06201.x

[56] V. Tam , N. Patel , M. Turcotte , Y. Bossé , G. Paré , D. Meyre , Nat. Rev. Genet. 2019, 20, 467. 31068683 10.1038/s41576-019-0127-1

[57] M. Shiota , S. Nemoto , R. Ikegami , S. Tatarano , T. Kamoto , K. Kobayashi , H. Sakai , T. Igawa , T. Kamba , N. Fujimoto , A. Yokomizo , S. Naito , M. Eto , BJC Rep. 2024, 2, 69. 39516672 10.1038/s44276-024-00093-3 PMC11523954

[58] B. K. Brauer , Z. Chen , F. Beirow , J. Li , D. Meisinger , E. Capriotti , M. Schweizer , L. Wagner , J. Wienberg , L. Hobohm , L. Blume , W. Qiao , Y. Narimatsu , J. E. Carette , H. Clausen , D. Winter , T. Braulke , S. Jabs , M. Voss , EMBO J. 2024, 43, 6264. 39587297 10.1038/s44318-024-00305-z PMC11649813

[59] L. G. Welch , S.‐Y. Peak‐Chew , F. Begum , T. J. Stevens , S. Munro , J. Cell Biol. 2021, 220, 202106115. 10.1083/jcb.202106115 PMC8421267 34473204

[60] S. Luo , C. Kong , S. Zhao , X. Tang , Y. Wang , X. Zhou , R. Li , X. Liu , X. Tang , S. Sun , W. Xie , Z.‐R. Zhang , Q. Jing , A. Gu , F. Chen , D. Wang , H. Wang , Y. Han , L. Xie , Y. Ji , Circulation 2023, 147, 1382. 36951067 10.1161/CIRCULATIONAHA.122.062743

[61] D. Ye , C. Wu , L. Cai , D. A. Howatt , C.‐L. Liang , Y. Katsumata , A. E. Mullick , R. E. Temel , A. H. J. Danser , A. Daugherty , H. Lu , Glob. Transl. Med. 2023, 2, 288. 37293374 10.36922/gtm.288 PMC10249463

[62] D. Ma , X. Liu , J.‐J. Zhang , J.‐J. Zhao , Y.‐J. Xiong , Q. Chang , H.‐Y. Wang , P. Su , J. Meng , Y.‐B. Zhao , Front. Cardiovasc. Med. 2020, 7, 592550. 33330653 10.3389/fcvm.2020.592550 PMC7715013

[63] L. Cong , F. A. Ran , D. Cox , S. Lin , R. Barretto , N. Habib , P. D. Hsu , X. Wu , W. Jiang , L. A. Marraffini , F. Zhang , Science 2013, 339, 819. 23287718 10.1126/science.1231143 PMC3795411

[64] M. He , B. Zheng , Y. Zhang , X.‐H. Zhang , C. Wang , Z. Yang , Y. Sun , X.‐L. Wu , J.‐K. Wen , FASEB J. 2015, 9, 4059. 10.1096/fj.15-272658 26082460

[65] J. R. Wiśniewski , A. Zougman , N. Nagaraj , M. Mann , Nat. Methods 2009, 6, 359. 19377485 10.1038/nmeth.1322

[66] K. Yu , B. Zheng , M. Han , J.‐K. Wen , Cardiovasc. Res. 2011, 90, 464. 21252119 10.1093/cvr/cvr017

[67] F. Zhang , K. Li , W. Zhang , Z. Zhao , F. Chang , J. Du , X. Zhang , K. Bao , C. Zhang , L. Shi , Z. Liu , X. Dai , C. Chen , D. W. Wang , Z. Xian , H. Jiang , D. Ai , Circulation 2024, 149, 843. 38018467 10.1161/CIRCULATIONAHA.123.066110

[68] L. Chen , W. Cao , R. Aita , D. Aldea , J. Flores , N. Gao , E. M. Bonder , C. E. Ellison , M. P. Verzi , Cell Rep. 2021, 34, 108679. 33503426 10.1016/j.celrep.2020.108679 PMC7899294

[69] J. Wang , Y. Zhao , X. Zhou , S. W. Hiebert , Q. Liu , Y. Shyr , BMC Genomics 2018, 19, 633. 30139328 10.1186/s12864-018-5016-z PMC6107967

[70] S. Hayashi , I. Manabe , Y. Suzuki , F. Relaix , Y. Oishi , eLife 2015, 5, 17462. 10.7554/eLife.17462 PMC5074804 27743478

[71] P. Yan , Z. Liu , M. Song , Z. Wu , W. Xu , K. Li , Q. Ji , S. Wang , X. Liu , K. Yan , C. R. Esteban , W. Ci , J. C. I. Belmonte , W. Xie , J. Ren , W. Zhang , Q. Sun , J. Qu , G.‐H. Liu , Cell Rep. 2020, 32, 107870. 32640235 10.1016/j.celrep.2020.107870

