# Supplementary material for: Ythdc1‐p300‐Klf5 Complex‐Mediated Golgi Dysfunction Promotes Aortic Aneurysm
Source: Adv Sci (Weinh). 2025 Nov 29;13(4):e12116. doi: 10.1002/advs.202512116 (PMC12822387; doi:10.1002/advs.202512116)

Extended Data fig. 1

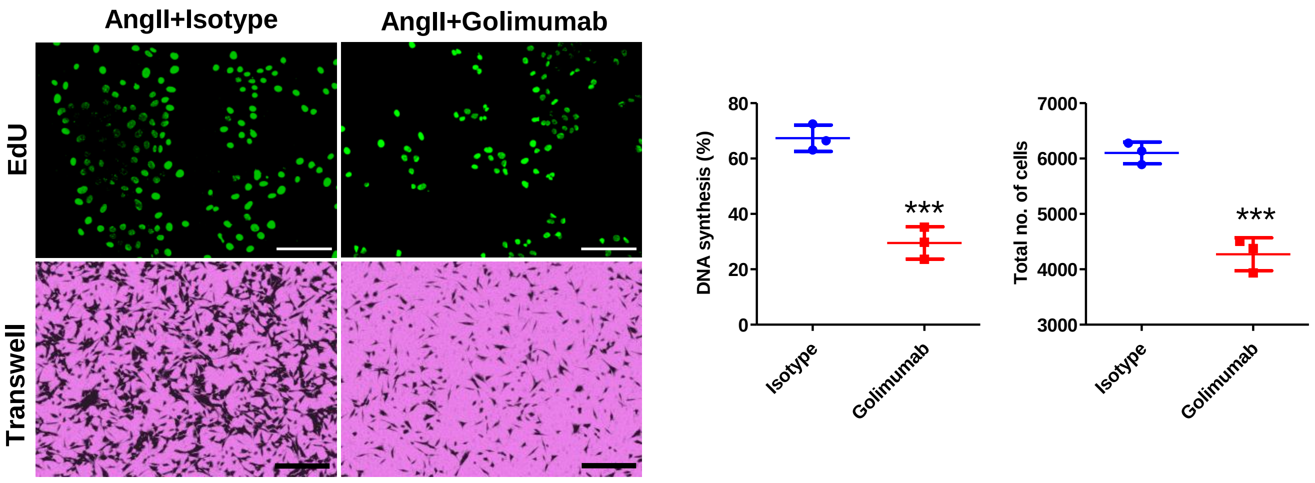

Extended Data fig. 2

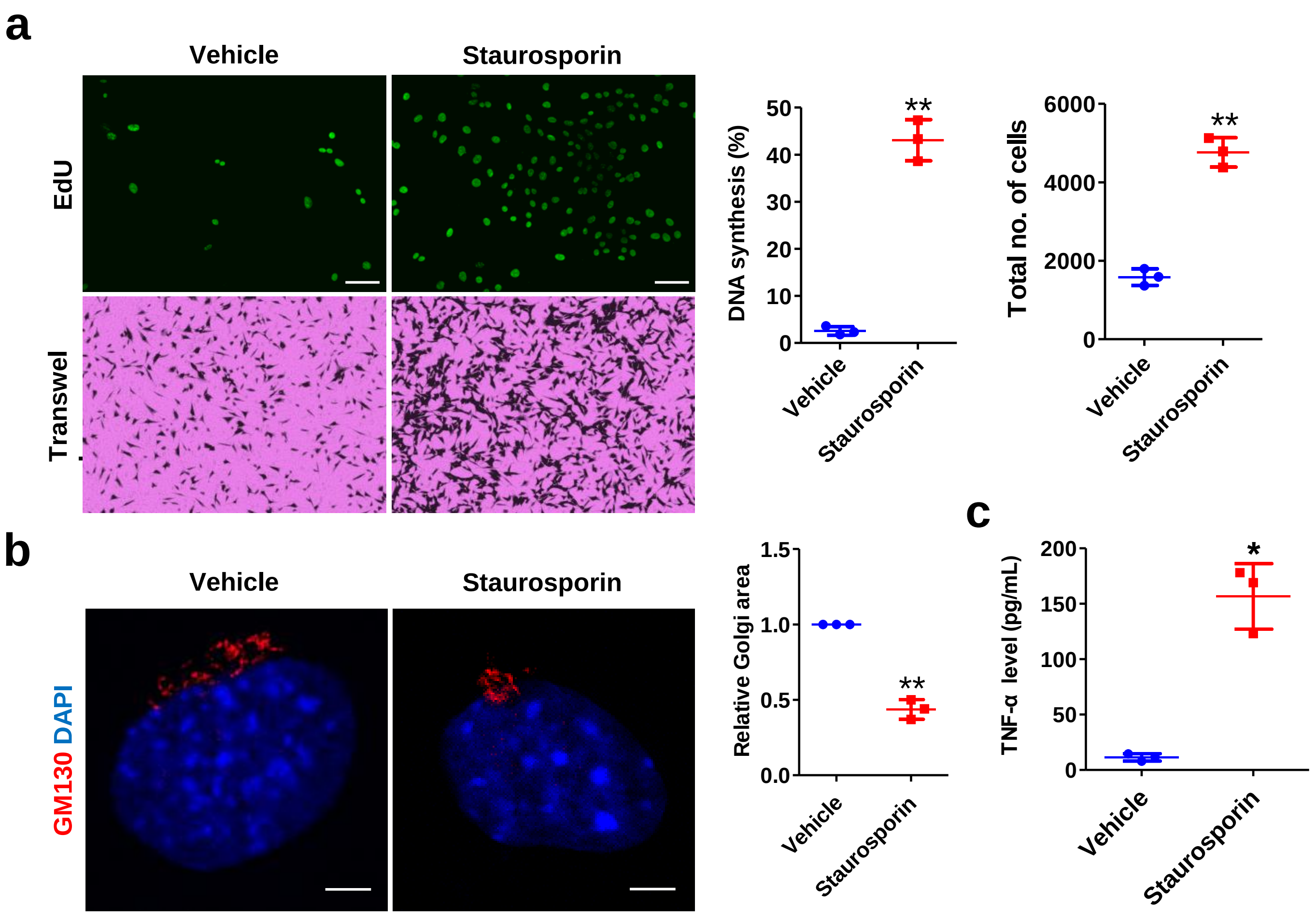

Extended Data fig. 3

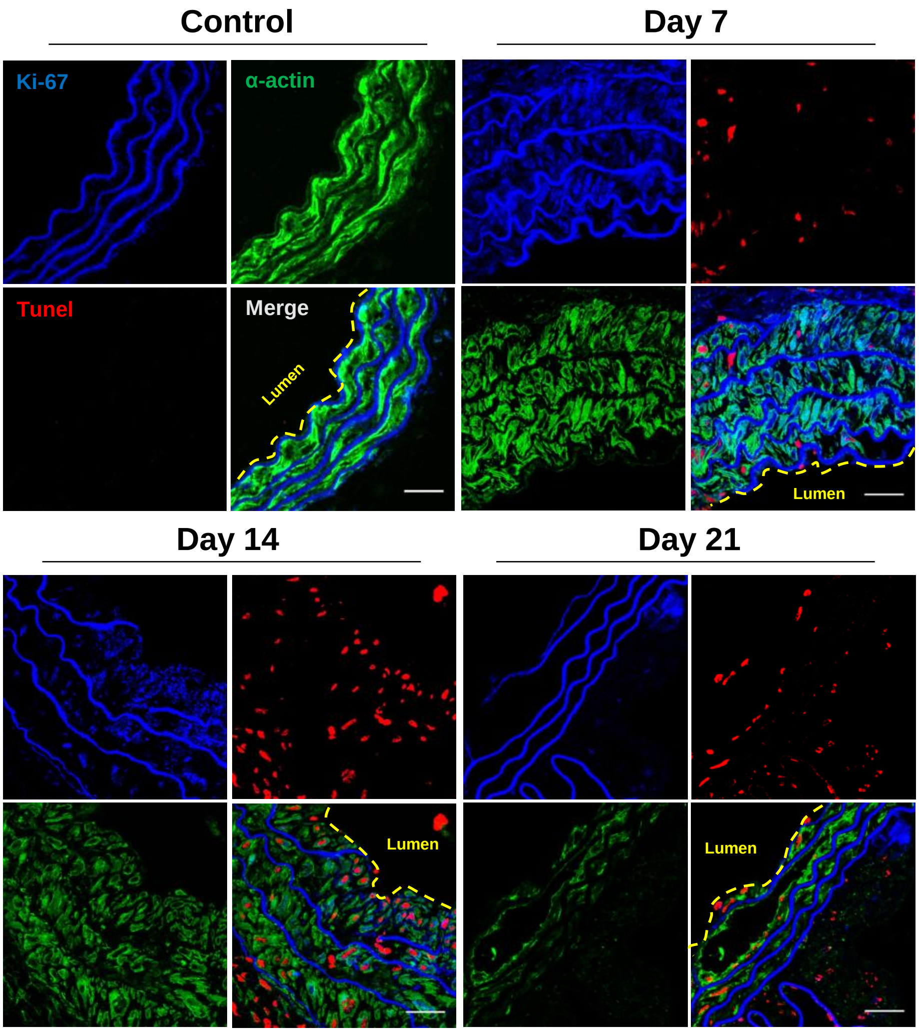

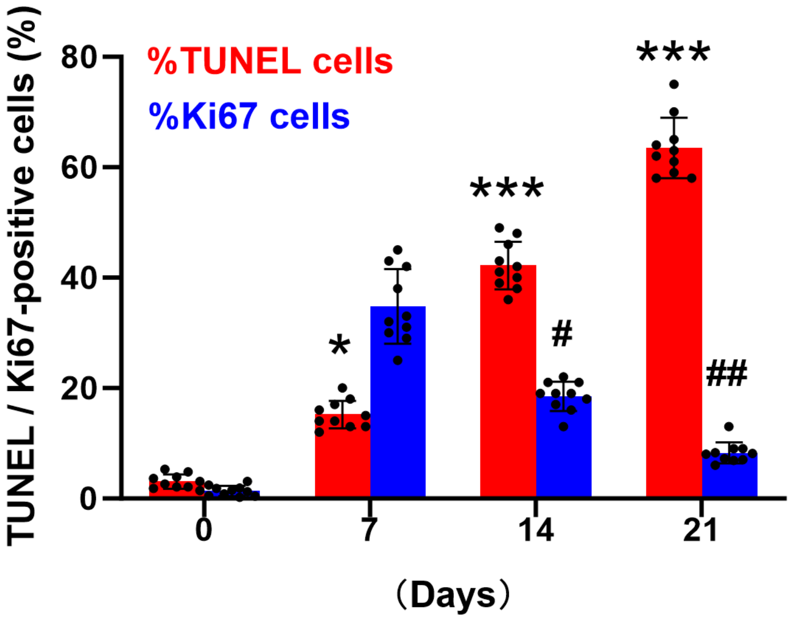

Supplemental figure 4

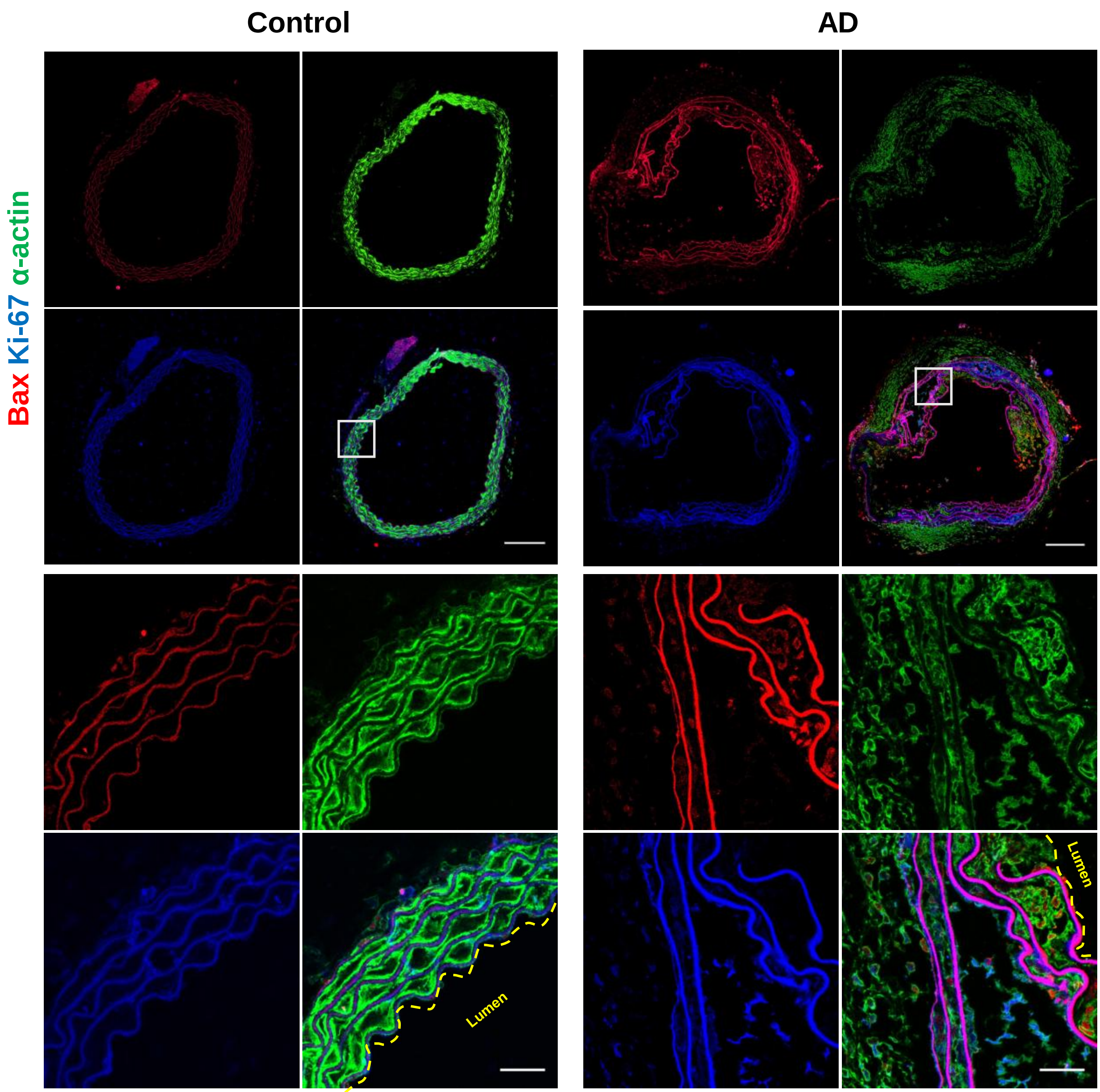

Extended Data fig. 5

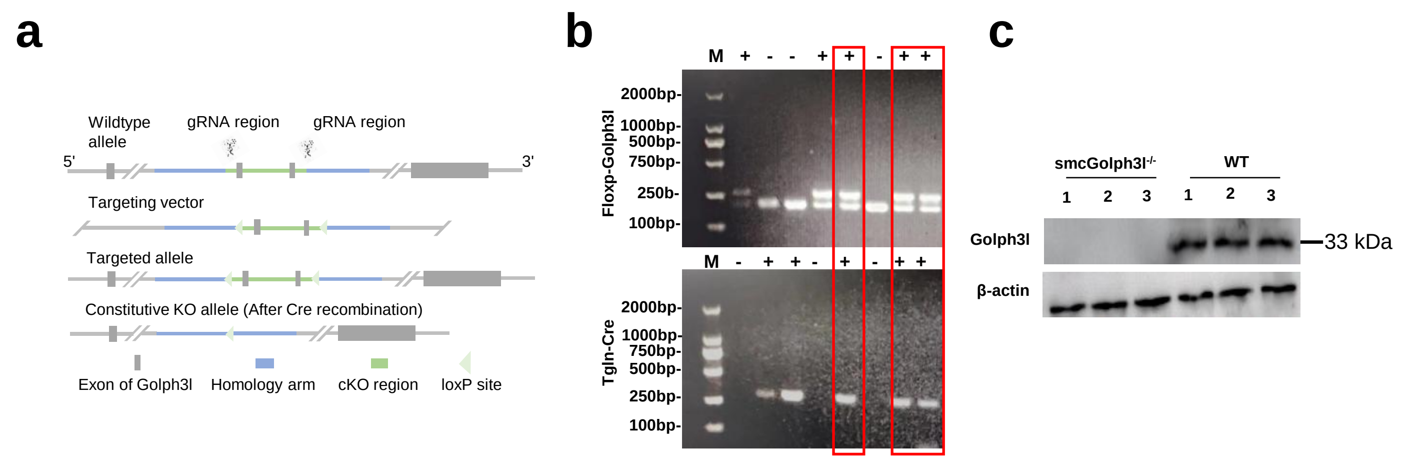

Extended Data fig. 6

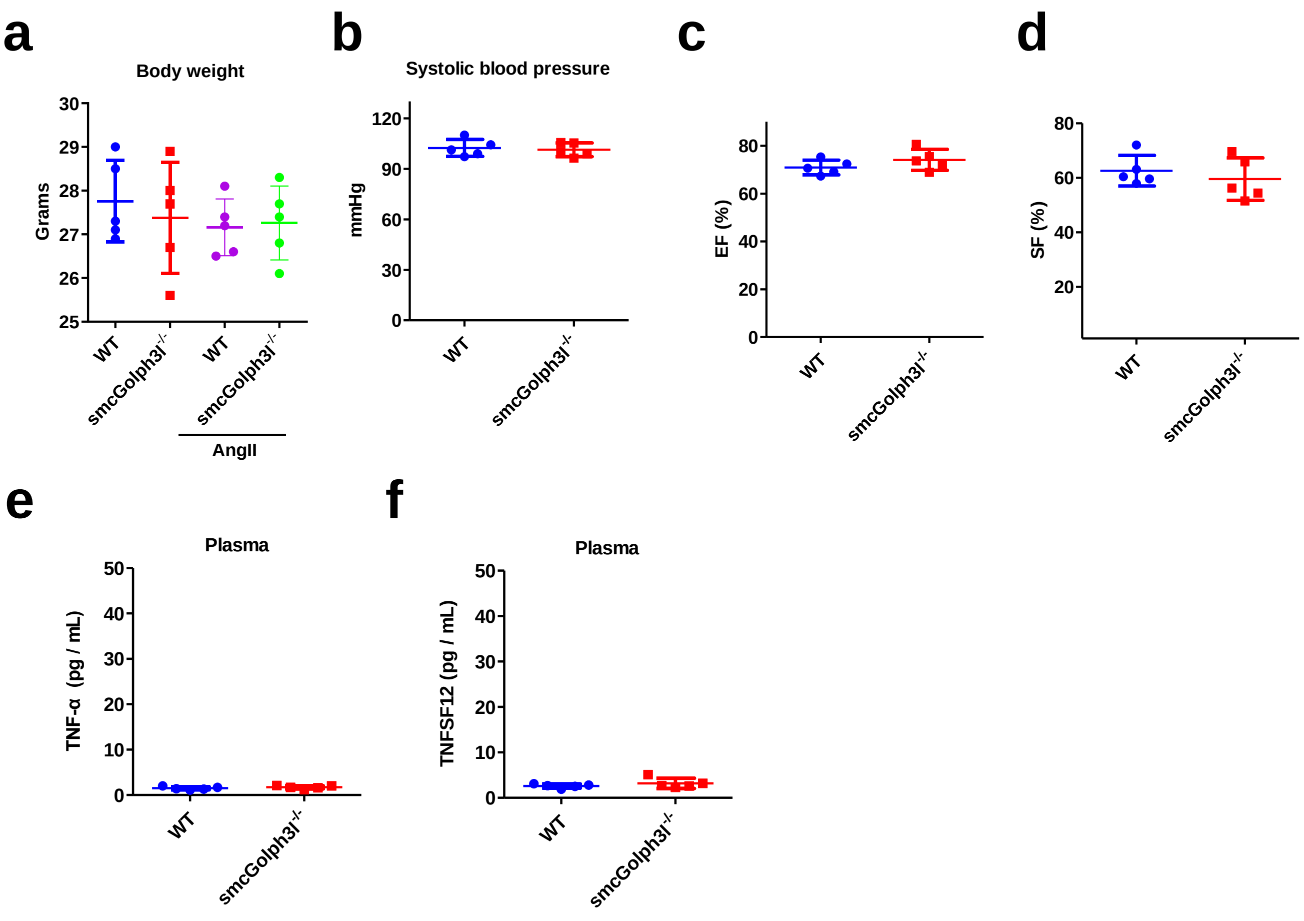

Extended Data fig. 7

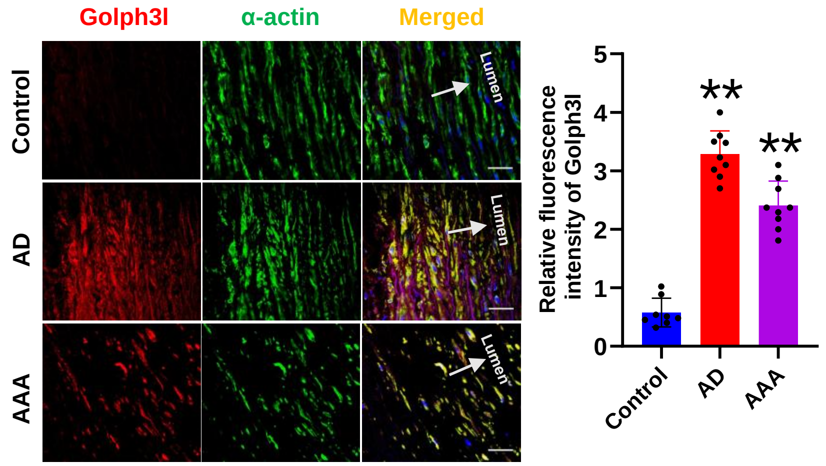

Extended Data fig. 8

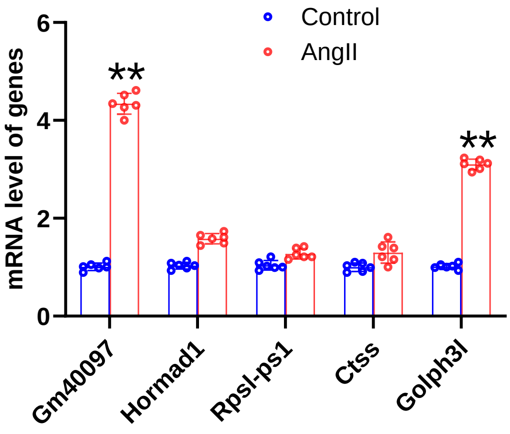

Extended Data fig. 9

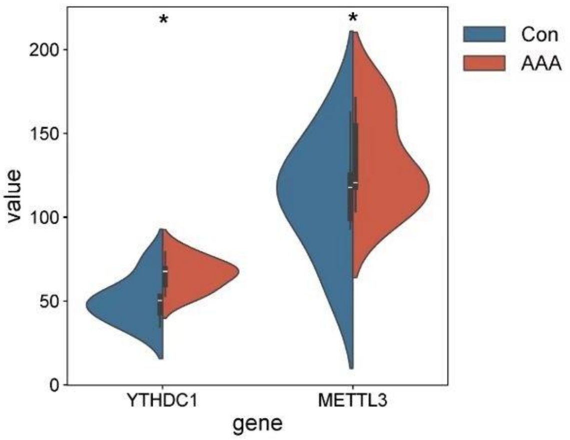

Extended Data fig. 10

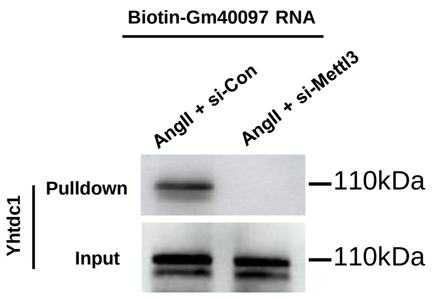

Extended Data fig. 11

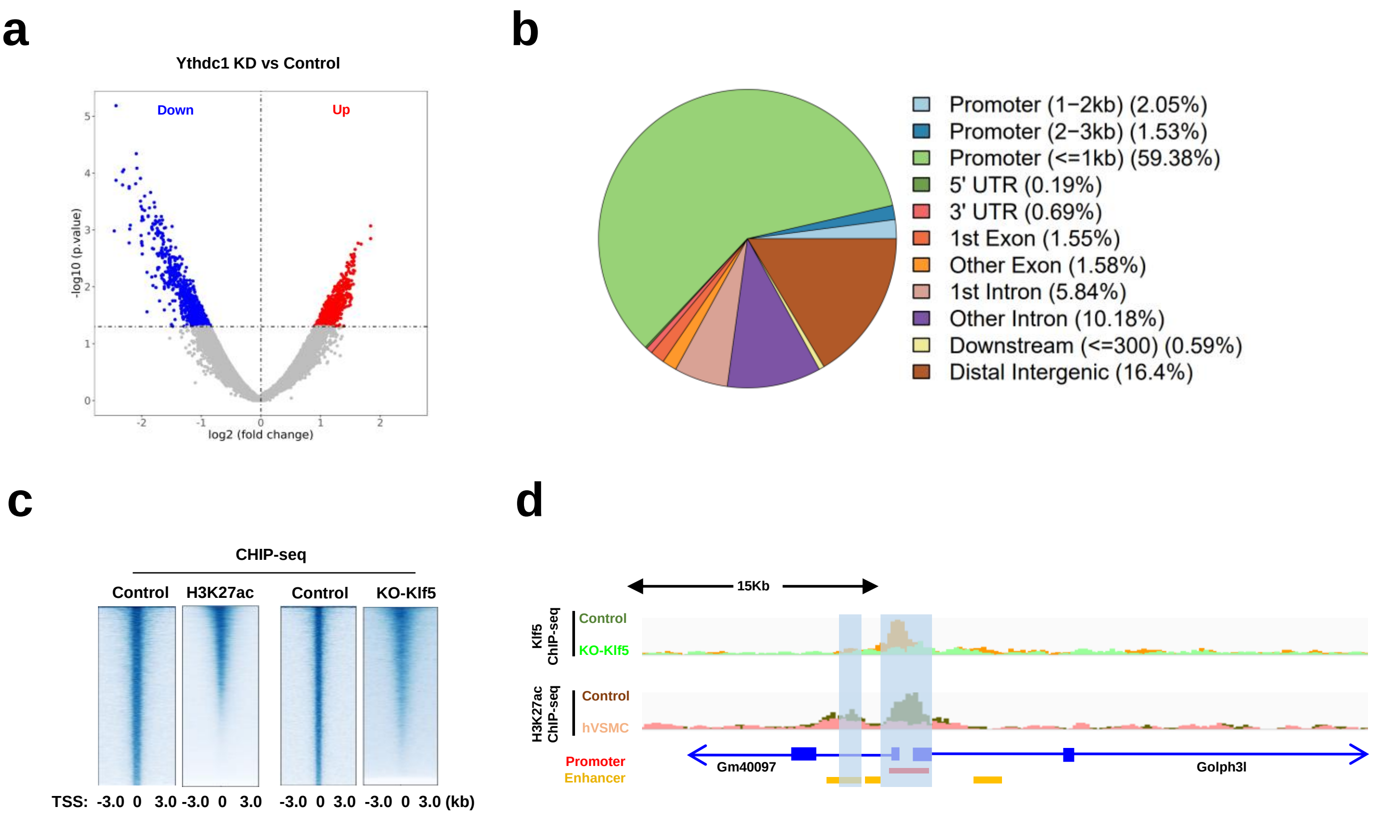

Extended Data fig. 12

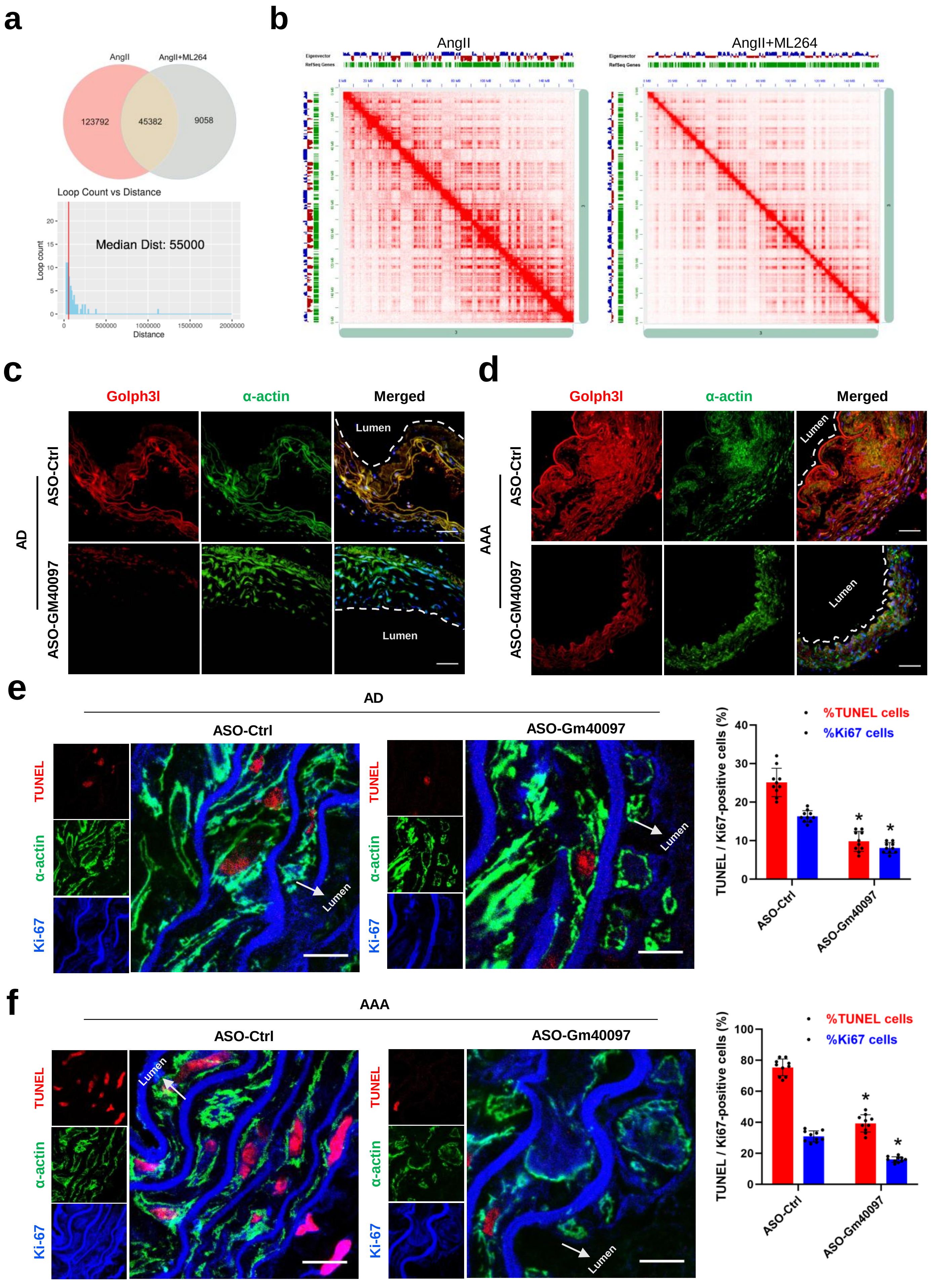

Supplement: Supplementary file 1 — Supporting Information [file ADVS-13-e12116-s001.pdf]
