# Supplementary material for: Ythdc1‐p300‐Klf5 Complex‐Mediated Golgi Dysfunction Promotes Aortic Aneurysm
Source: Adv Sci (Weinh). 2025 Nov 29;13(4):e12116. doi: 10.1002/advs.202512116 (PMC12822387; doi:10.1002/advs.202512116)

Extended Data fig. 13

Fig. 3b

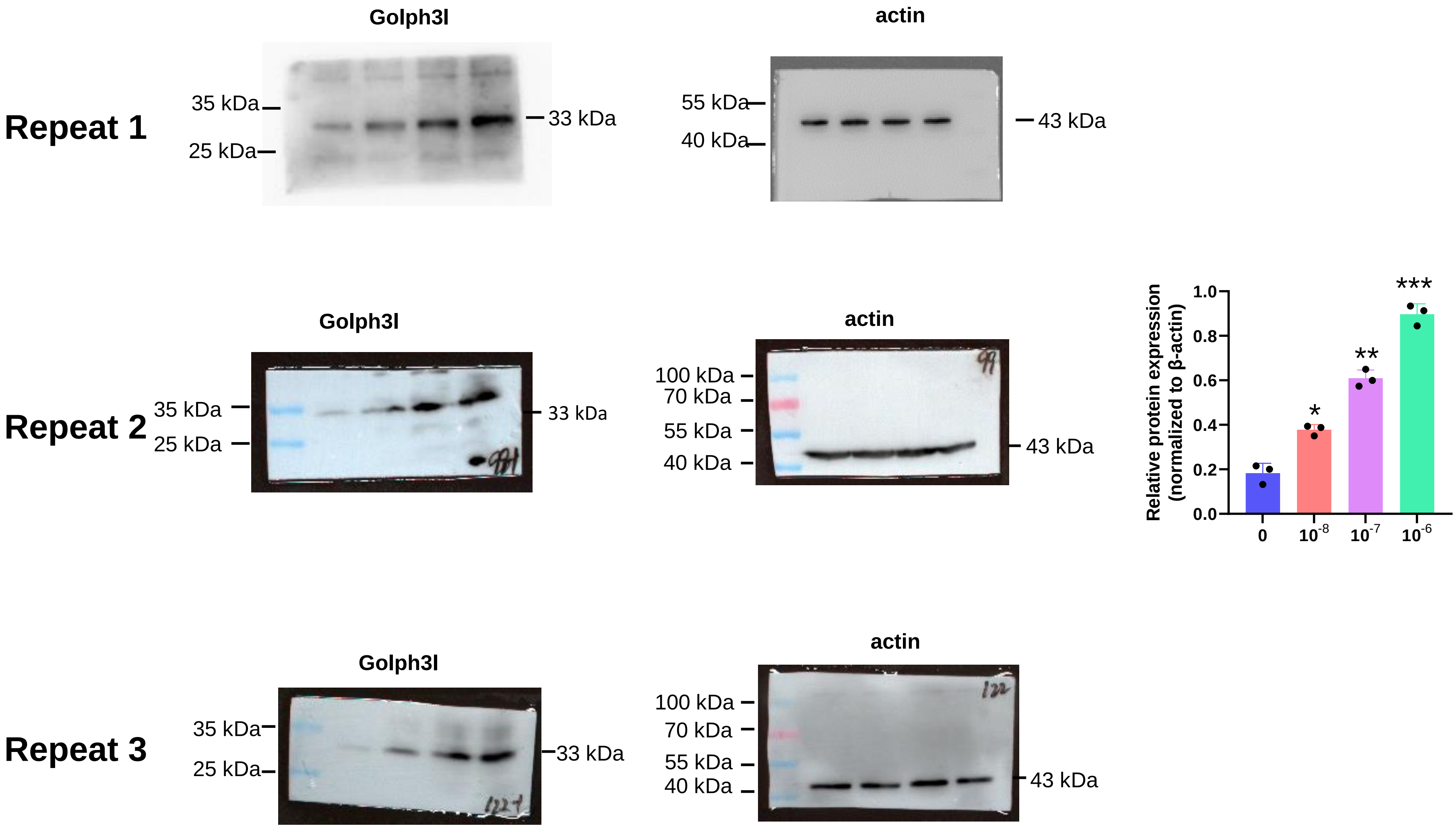

Fig. 3n

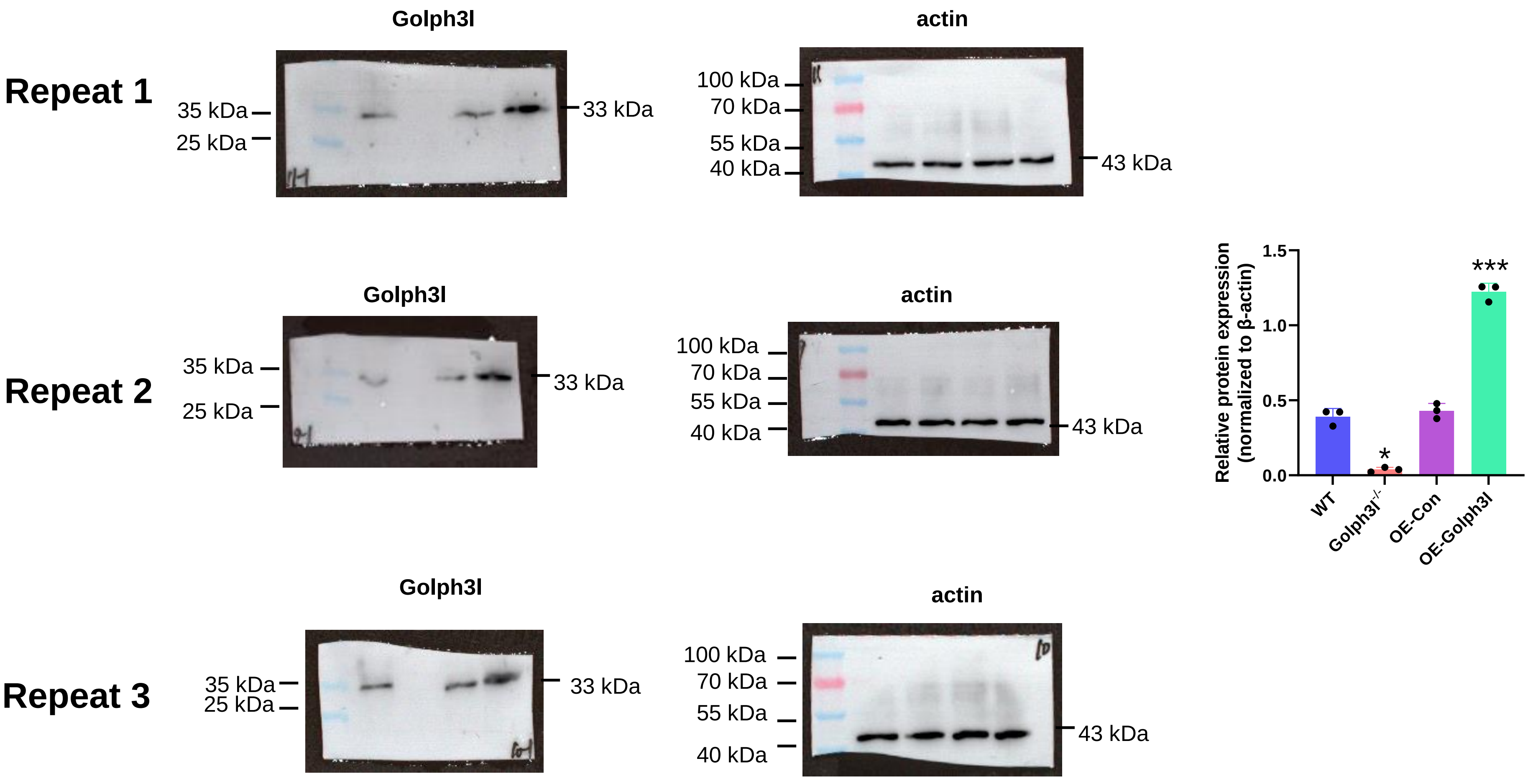

Repeat 1
Golph3l
35 kDa
25 kDa
33 kDa
actin
100 kDa
70 kDa
55 kDa
40 kDa
43 kDa
Repeat 2
Golph3l
35 kDa
25 kDa
33 kDa
actin
100 kDa
70 kDa
55 kDa
40 kDa
43 kDa
Repeat 3
Golph3l
35 kDa
25 kDa
33 kDa
actin
100 kDa
70 kDa
55 kDa
40 kDa
43 kDa
Relative protein expression (normalized to β-actin)
1.5
1.0
0.5
0.0
***
*
WT
Golph3l-/-
OE-Con
OE-Golph3l

Fig. 4c

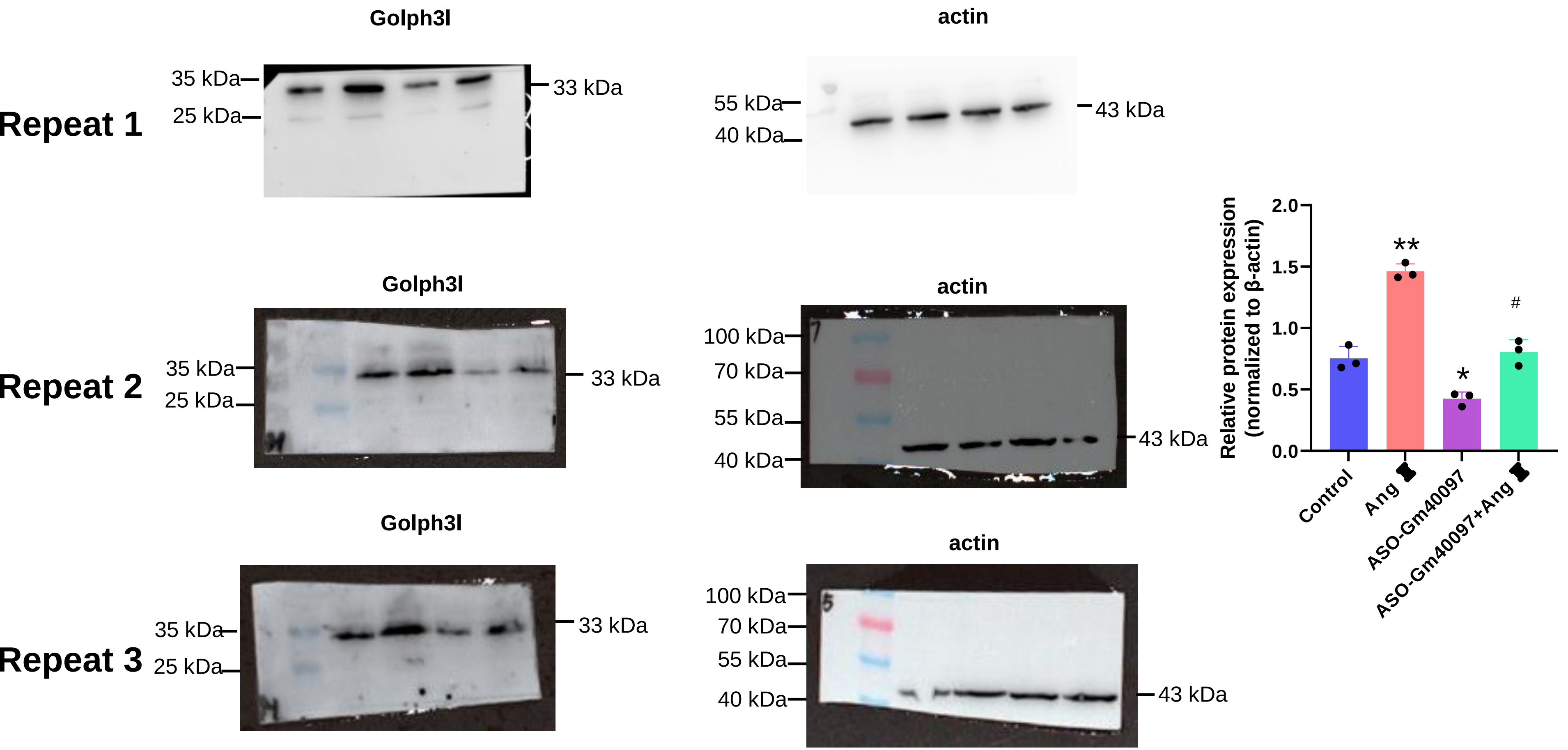

Golph3l
actin
Repeat 1
35 kDa
25 kDa
33 kDa
55 kDa
40 kDa
43 kDa
Repeat 2
35 kDa
25 kDa
33 kDa
100 kDa
70 kDa
55 kDa
40 kDa
43 kDa
Repeat 3
35 kDa
25 kDa
33 kDa
100 kDa
70 kDa
55 kDa
40 kDa
43 kDa
Relative protein expression
(normalized to β-actin)
2.0
1.5
1.0
0.5
0.0
**
*
#
Control
Ang
ASO-Gm40097
ASO-Gm40097+Ang

Fig. 4d

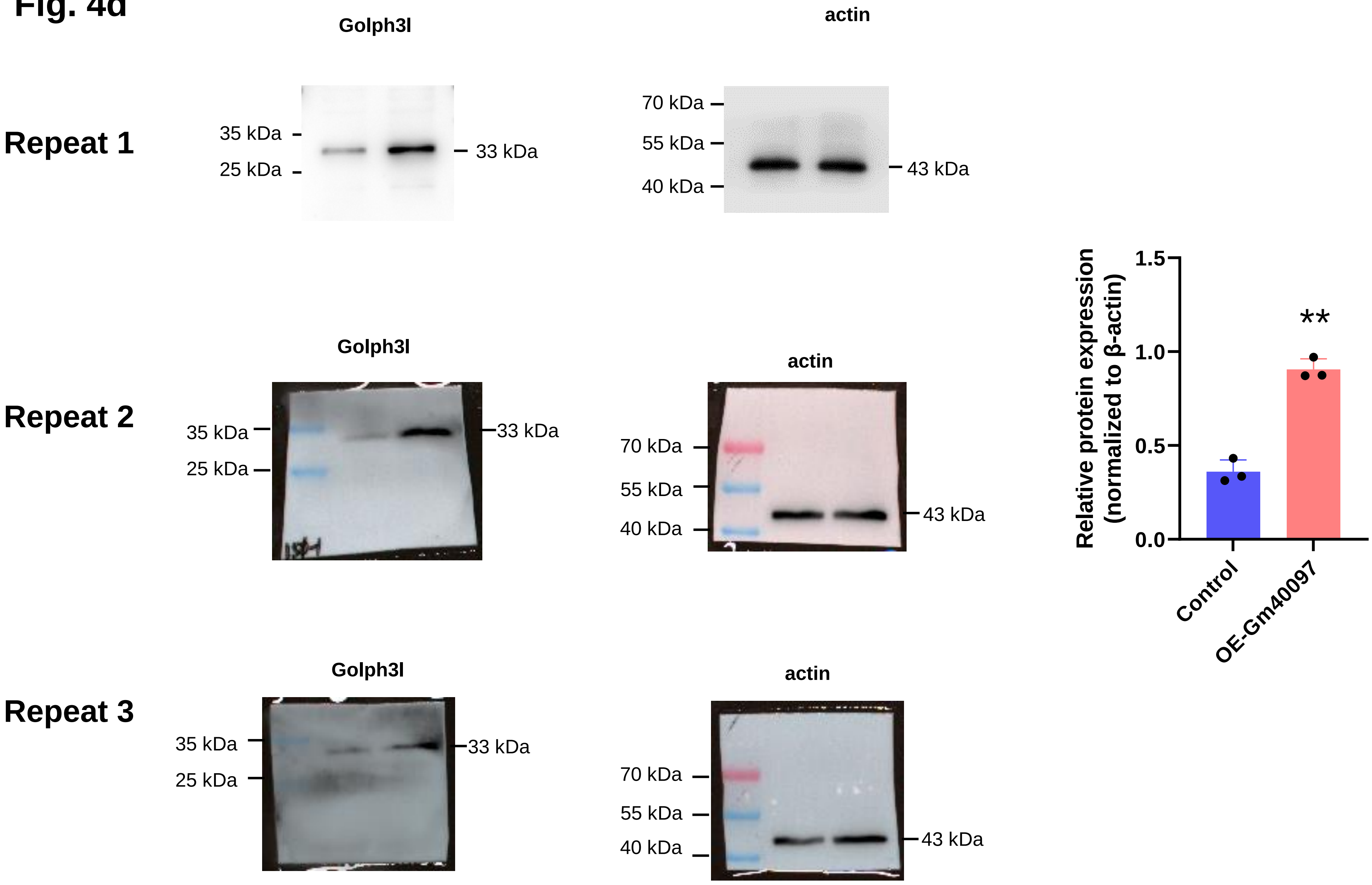
Golph3l
actin
Repeat 1
35 kDa
25 kDa
33 kDa
70 kDa
55 kDa
40 kDa
43 kDa
Repeat 2
Golph3l
35 kDa
25 kDa
33 kDa
actin
70 kDa
55 kDa
40 kDa
43 kDa
Repeat 3
Golph3l
35 kDa
25 kDa
33 kDa
actin
70 kDa
55 kDa
40 kDa
43 kDa
Relative protein expression
(normalized to β-actin)
1.5
1.0
0.5
0.0
**
Control
OE-Gm40097

Fig. 4m

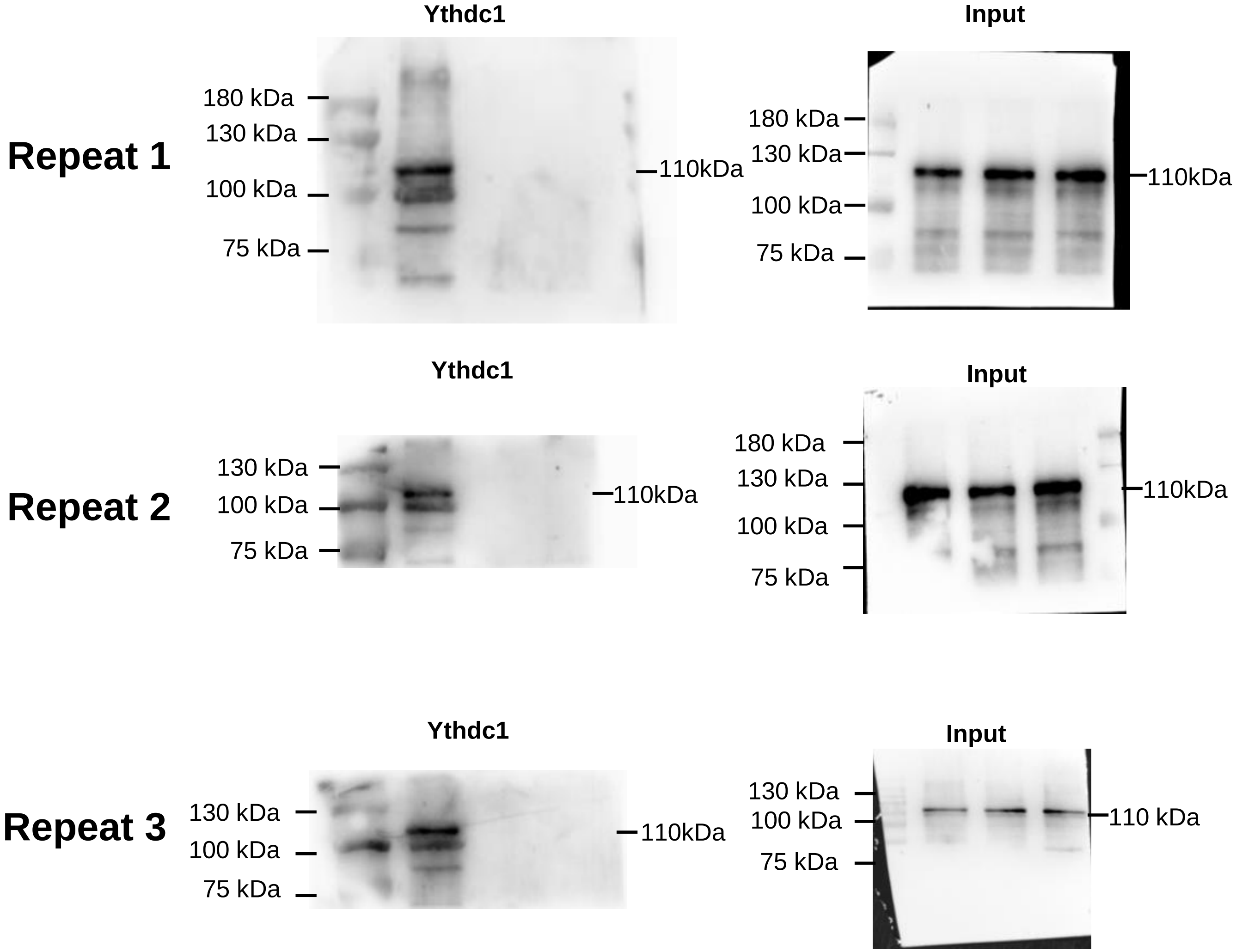

Repeat 1
Ythdc1
180 kDa
130 kDa
100 kDa
75 kDa
110kDa
Input
180 kDa
130 kDa
100 kDa
75 kDa
110kDa
Repeat 2
Ythdc1
130 kDa
100 kDa
75 kDa
110kDa
Input
180 kDa
130 kDa
100 kDa
75 kDa
110kDa
Repeat 3
Ythdc1
130 kDa
100 kDa
75 kDa
110kDa
Input
130 kDa
100 kDa
75 kDa
110 kDa

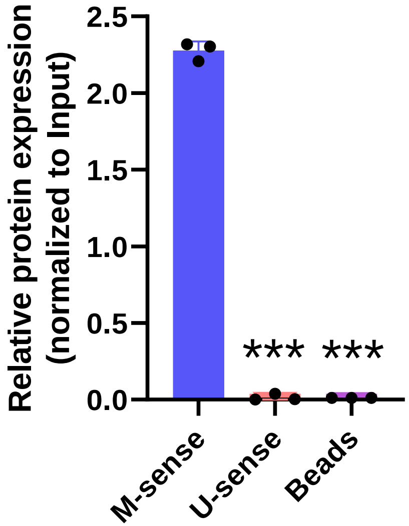

Relative protein expression
(normalized to Input)
2.5
2.0
1.5
1.0
0.5
0.0
***
***
M-sense
U-sense
Beads

Fig. 4m

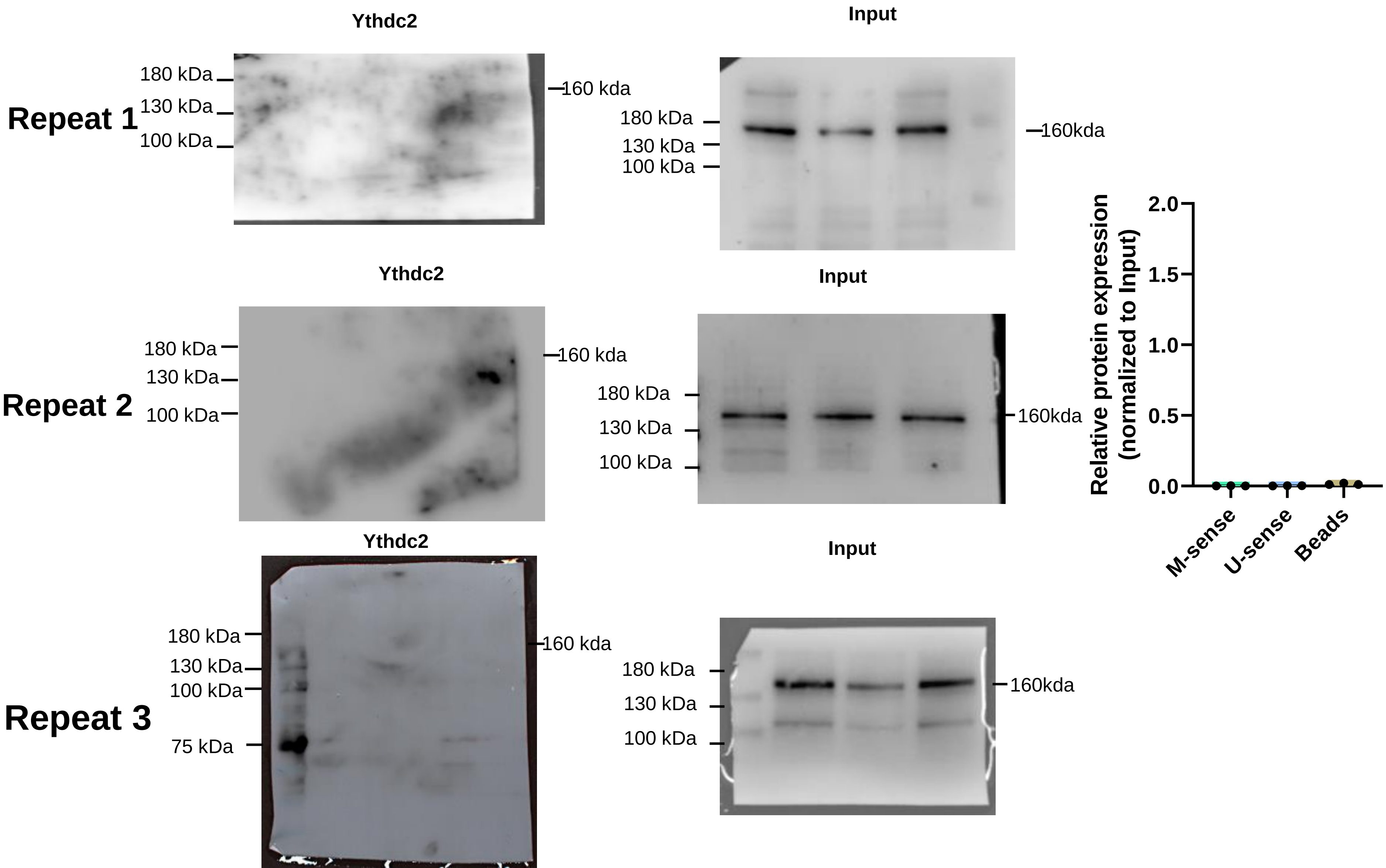
Ythdc2
Input
Repeat 1
180 kDa
130 kDa
100 kDa
160 kda
180 kDa
130 kDa
100 kDa
160kda
Ythdc2
Input
Repeat 2
180 kDa
130 kDa
100 kDa
160 kda
180 kDa
130 kDa
100 kDa
160kda
Ythdc2
Input
Repeat 3
180 kDa
130 kDa
100 kDa
75 kDa
160 kda
180 kDa
130 kDa
100 kDa
160kda
Relative protein expression (normalized to Input)
2.0
1.5
1.0
0.5
0.0
M-sense
U-sense
Beads

Fig. 4p

Ythdc1

Repeat 1

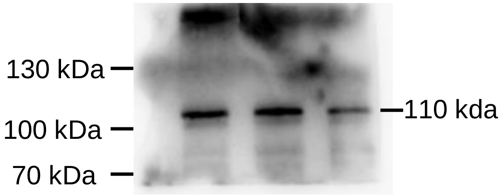

Ythdc1

Repeat 2

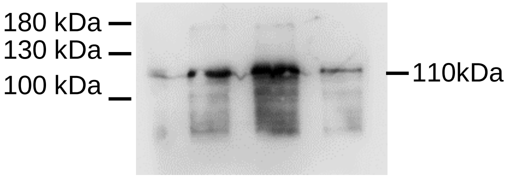

Ythdc1

Repeat 3

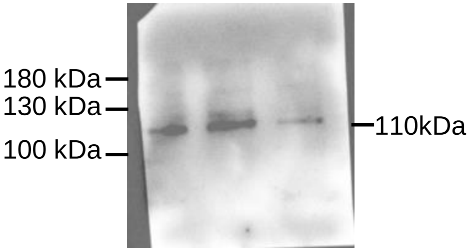

Fig. 4s

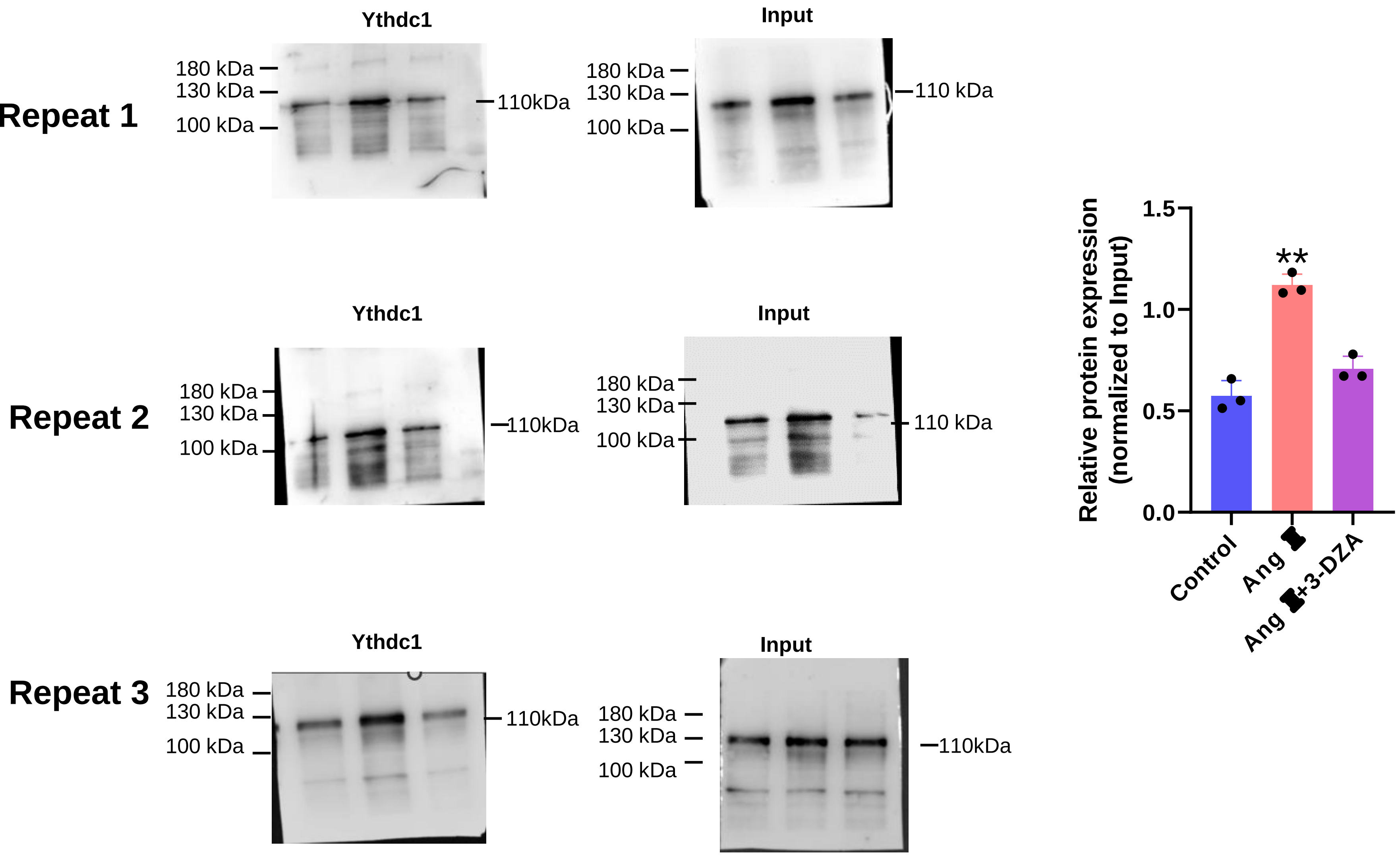
Repeat 1
Repeat 2
Repeat 3
Ythdc1
Input
180 kDa
130 kDa
100 kDa
110kDa
110 kDa
Relative protein expression
(normalized to Input)
1.5
1.0
0.5
0.0
**
Control
Ang [redacted]
Ang [redacted]+3-DZA

Fig. 5d

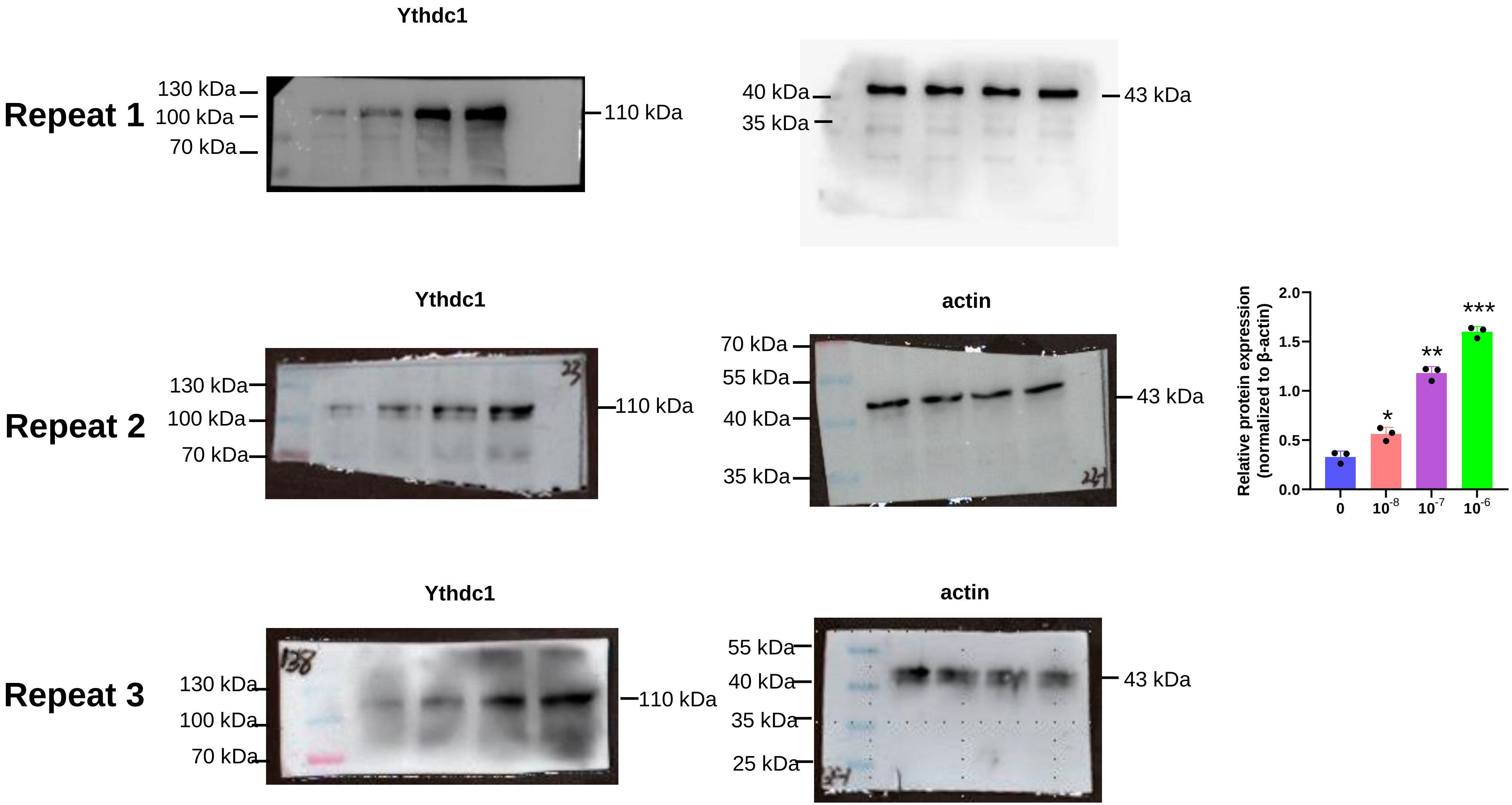
Ythdc1
Repeat 1
130 kDa
100 kDa
70 kDa
110 kDa
40 kDa
35 kDa
43 kDa
Ythdc1
actin
Repeat 2
130 kDa
100 kDa
70 kDa
110 kDa
70 kDa
55 kDa
40 kDa
35 kDa
43 kDa
Relative protein expression (normalized to β-actin)
2.0
1.5
1.0
0.5
0.0
*
**
***
0
10-8
10-7
10-6
Ythdc1
actin
Repeat 3
130 kDa
100 kDa
70 kDa
110 kDa
55 kDa
40 kDa
35 kDa
25 kDa
43 kDa

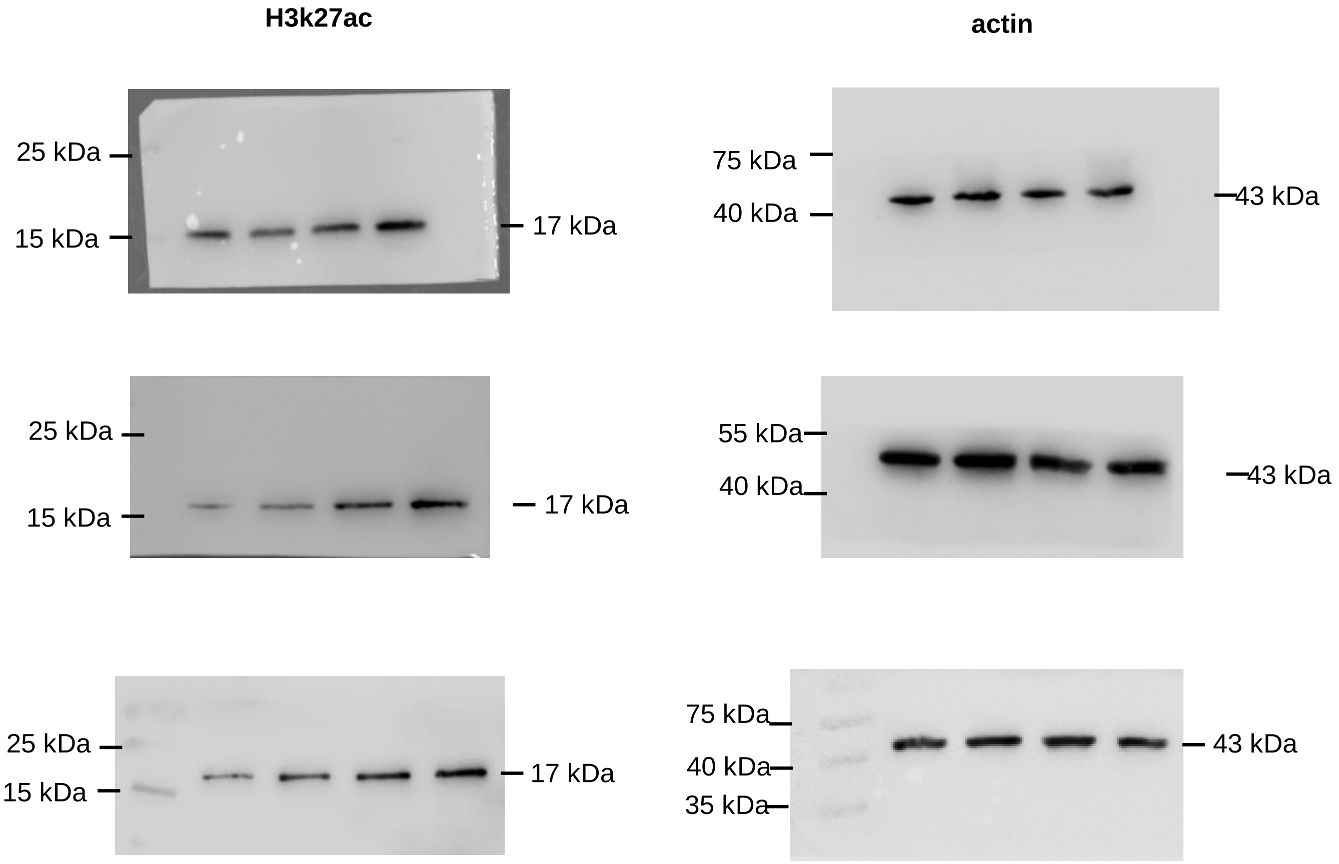
H3k27ac
actin
25 kDa
15 kDa
17 kDa
75 kDa
40 kDa
43 kDa
25 kDa
15 kDa
17 kDa
55 kDa
40 kDa
43 kDa
25 kDa
15 kDa
17 kDa
75 kDa
40 kDa
35 kDa
43 kDa

Fig. 5d

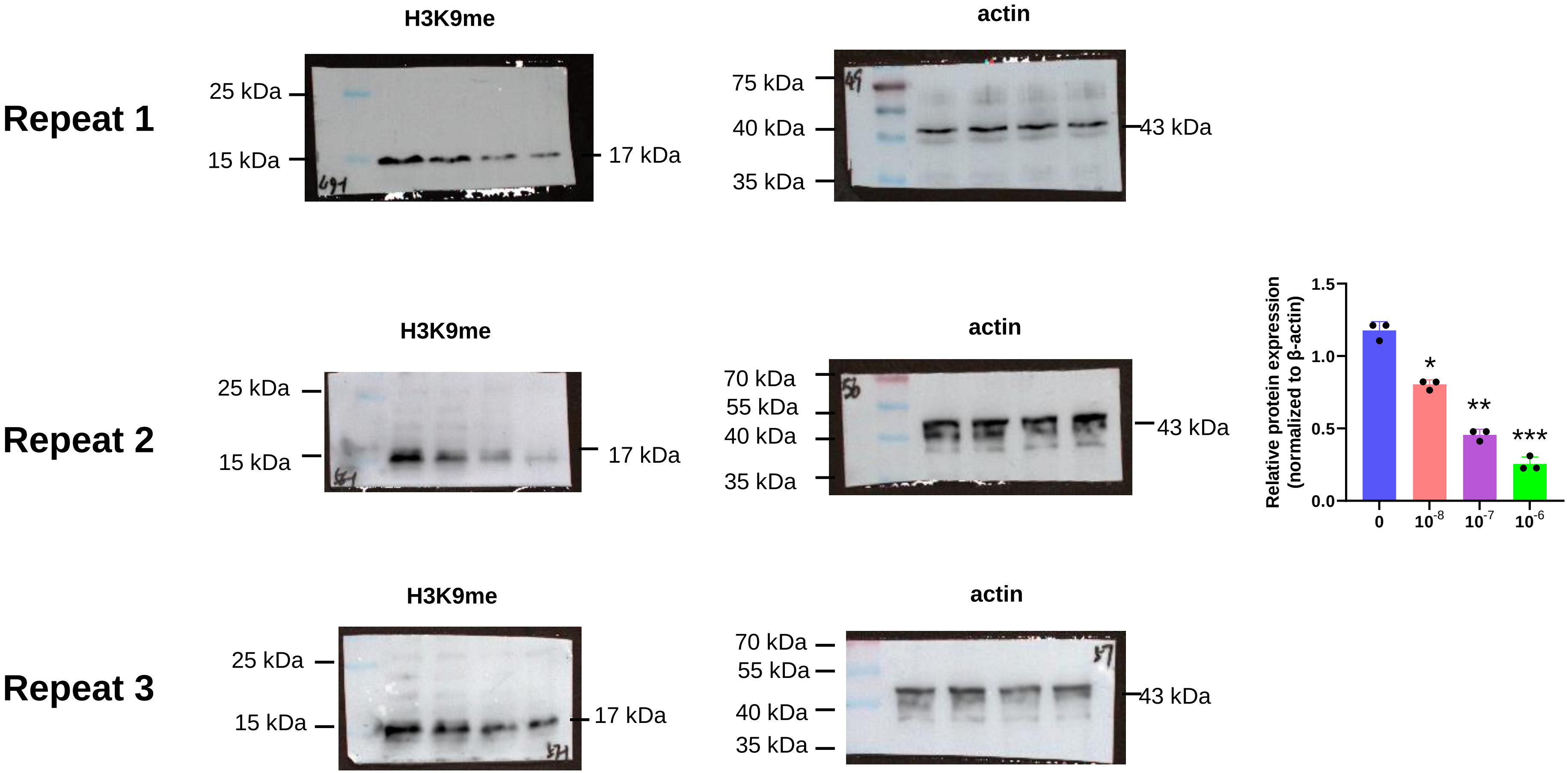
H3K9me
actin
Repeat 1
25 kDa
15 kDa
17 kDa
75 kDa
40 kDa
35 kDa
43 kDa
Repeat 2
25 kDa
15 kDa
17 kDa
70 kDa
55 kDa
40 kDa
35 kDa
43 kDa
Repeat 3
25 kDa
15 kDa
17 kDa
70 kDa
55 kDa
40 kDa
35 kDa
43 kDa
Relative protein expression
(normalized to β-actin)
1.5
1.0
0.5
0.0
0
10-8
10-7
10-6
*
**
***

Fig. 5d

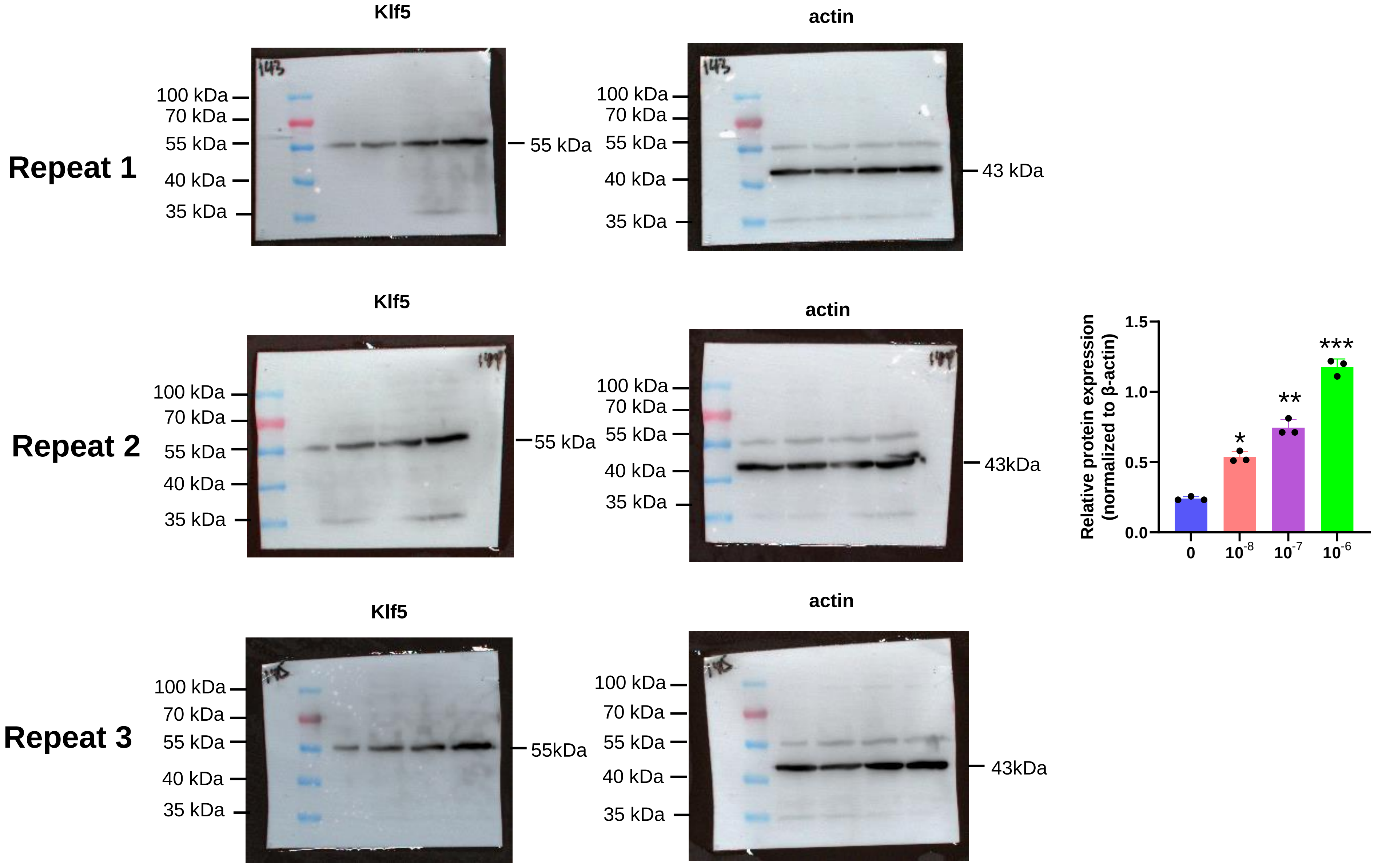

Klf5
actin
Repeat 1
Repeat 2
Repeat 3
100 kDa
70 kDa
55 kDa
40 kDa
35 kDa
55 kDa
43 kDa
55kDa
43kDa
Relative protein expression
(normalized to β-actin)
1.5
1.0
0.5
0.0
0
10-8
10-7
10-6
*
**
***

Fig. 5d

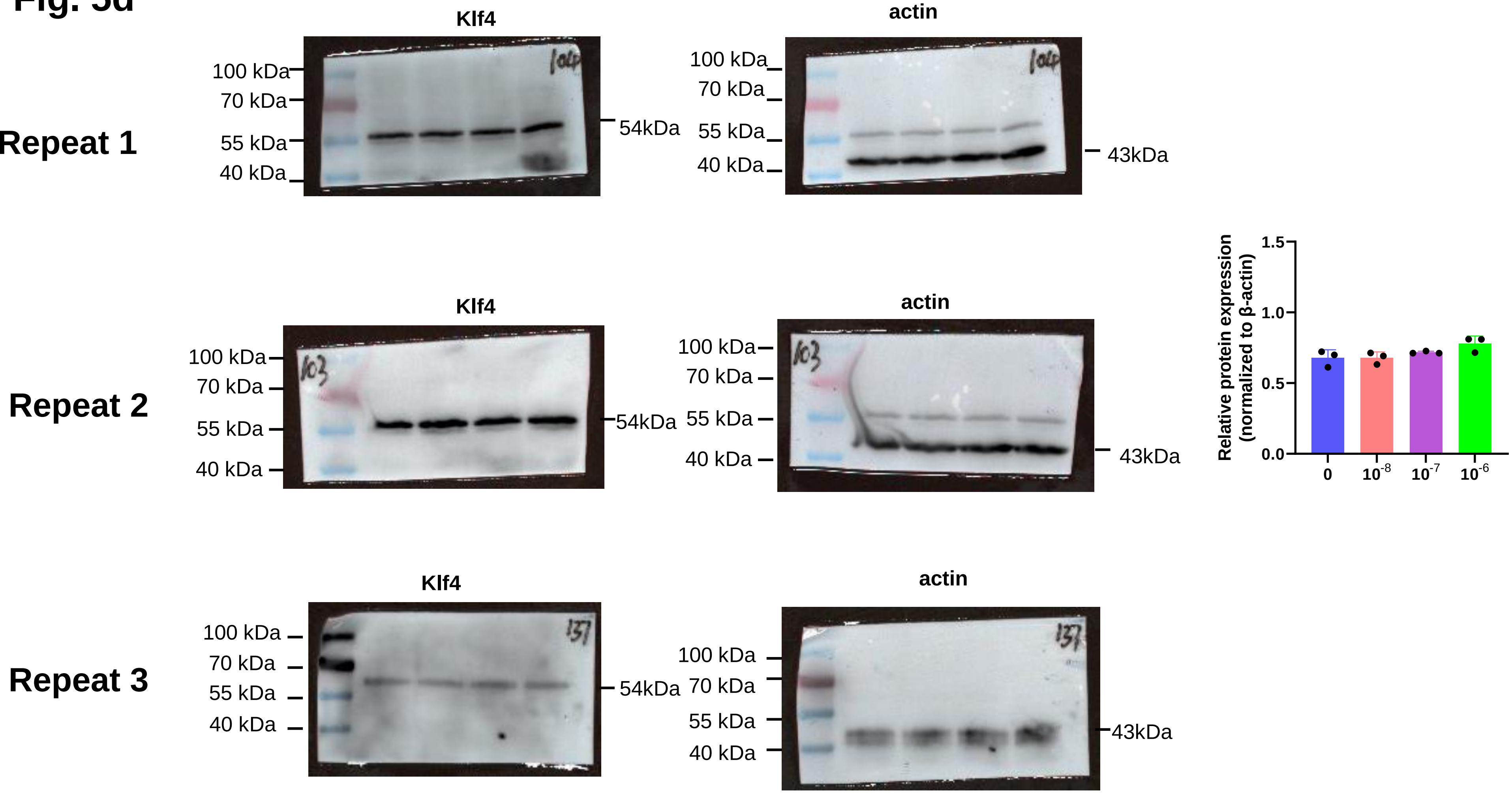

Klf4
actin
Repeat 1
100 kDa
70 kDa
55 kDa
40 kDa
54kDa
43kDa
Repeat 2
Repeat 3
Relative protein expression (normalized to β-actin)
1.5
1.0
0.5
0.0
0
10-8
10-7
10-6

Fig. 5d

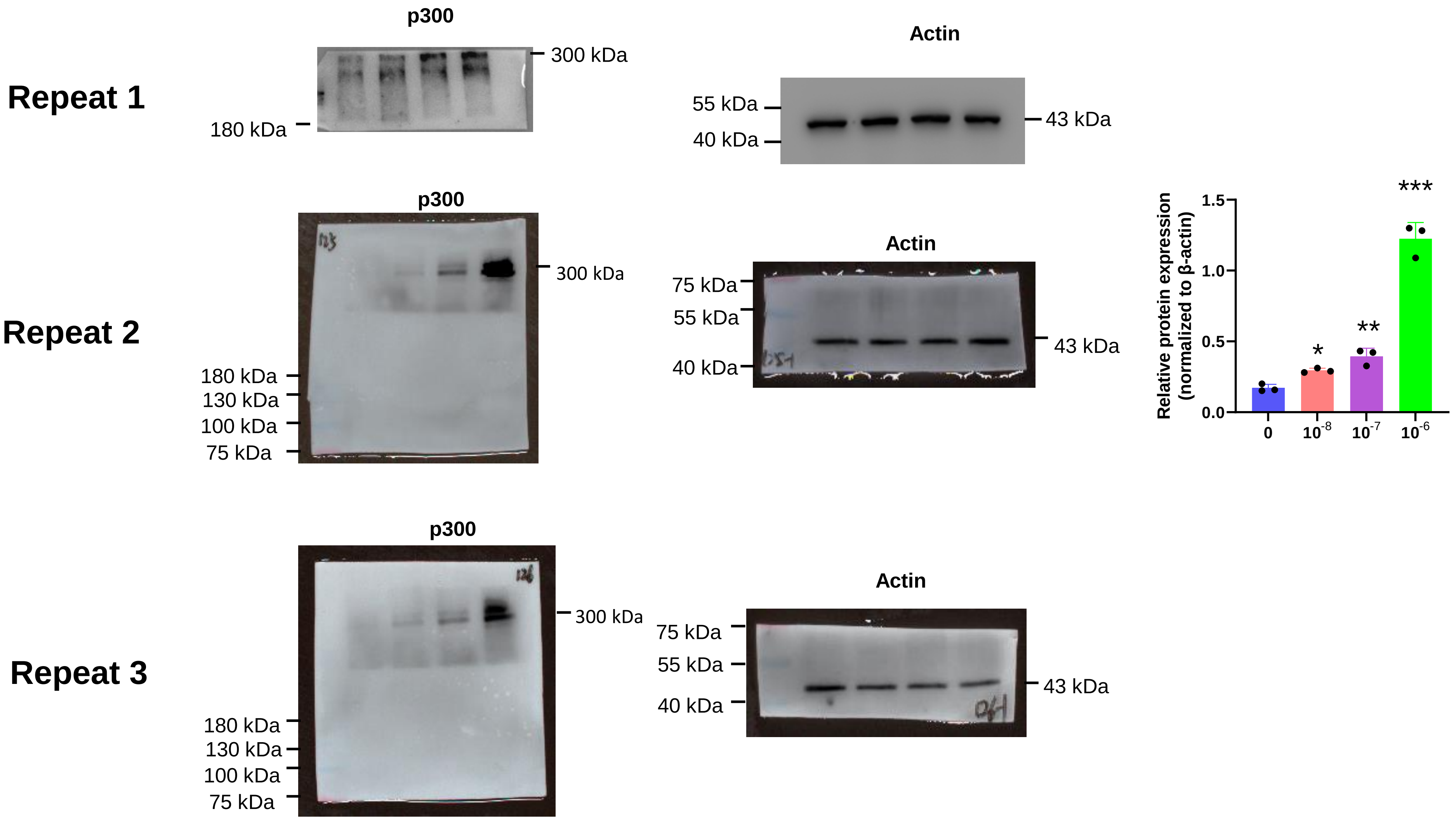

Repeat 1
p300
300 kDa
180 kDa
Actin
55 kDa
40 kDa
43 kDa
Repeat 2
p300
300 kDa
180 kDa
130 kDa
100 kDa
75 kDa
Actin
75 kDa
55 kDa
40 kDa
43 kDa
Repeat 3
p300
300 kDa
180 kDa
130 kDa
100 kDa
75 kDa
Actin
75 kDa
55 kDa
40 kDa
43 kDa
Relative protein expression (normalized to β-actin)
1.5
1.0
0.5
0.0
0
10-8
10-7
10-6
*
**
***

Fig. 5g

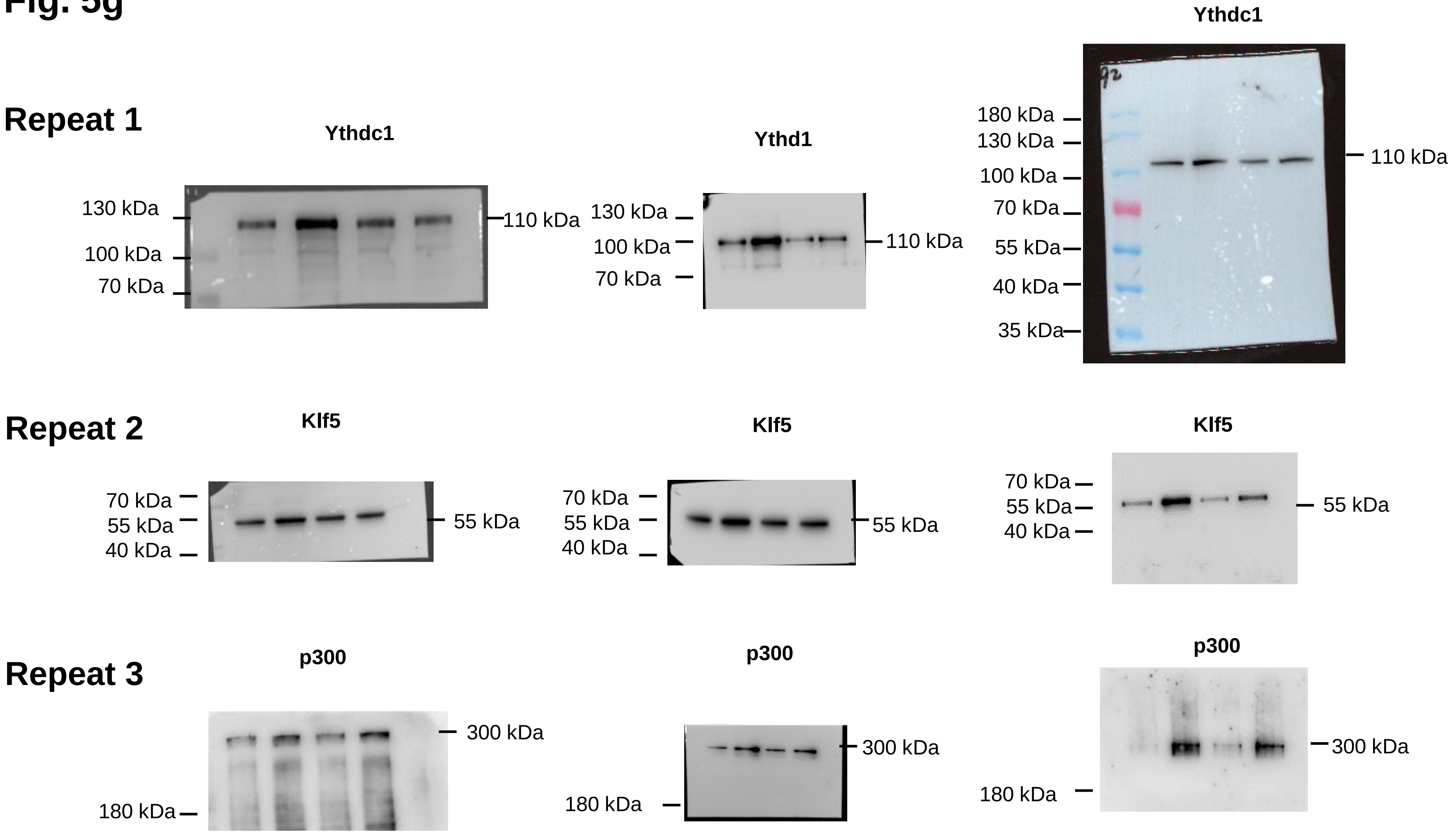

Repeat 1
Ythdc1
130 kDa
100 kDa
70 kDa
110 kDa
Ythd1
130 kDa
100 kDa
70 kDa
110 kDa
Ythdc1
180 kDa
130 kDa
100 kDa
70 kDa
55 kDa
40 kDa
35 kDa
110 kDa
Repeat 2
Klf5
70 kDa
55 kDa
40 kDa
55 kDa
Klf5
70 kDa
55 kDa
40 kDa
55 kDa
Klf5
70 kDa
55 kDa
40 kDa
55 kDa
Repeat 3
p300
300 kDa
180 kDa
p300
300 kDa
180 kDa
p300
300 kDa
180 kDa

Fig. 5g

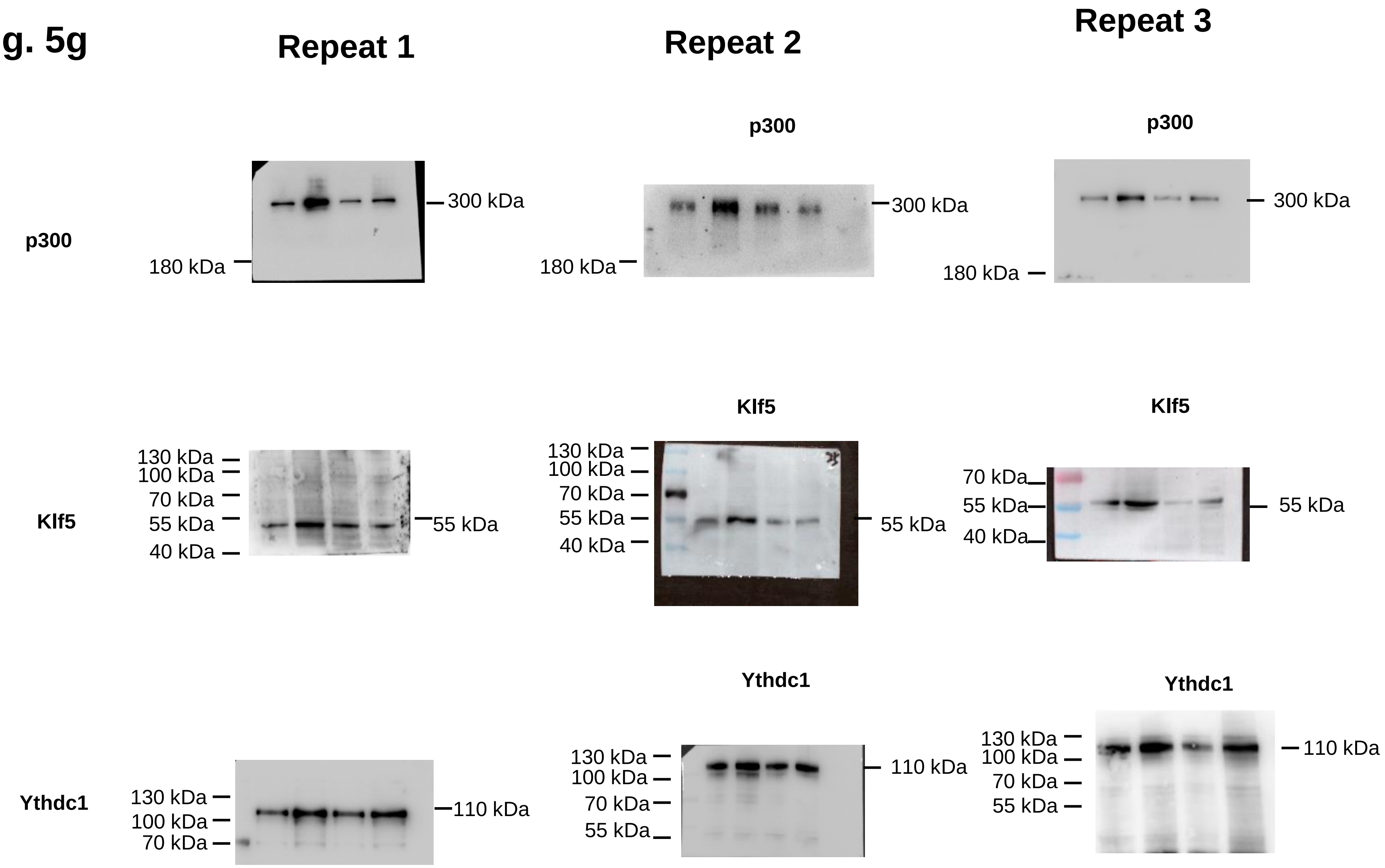
Repeat 1
Repeat 2
Repeat 3
p300
p300
p300
300 kDa
180 kDa
300 kDa
180 kDa
300 kDa
180 kDa
Klf5
Klf5
Klf5
130 kDa
100 kDa
70 kDa
55 kDa
40 kDa
55 kDa
130 kDa
100 kDa
70 kDa
55 kDa
40 kDa
55 kDa
70 kDa
55 kDa
40 kDa
55 kDa
Ythdc1
Ythdc1
Ythdc1
130 kDa
100 kDa
70 kDa
110 kDa
130 kDa
100 kDa
70 kDa
55 kDa
110 kDa
130 kDa
100 kDa
70 kDa
55 kDa
110 kDa

Fig. 5g

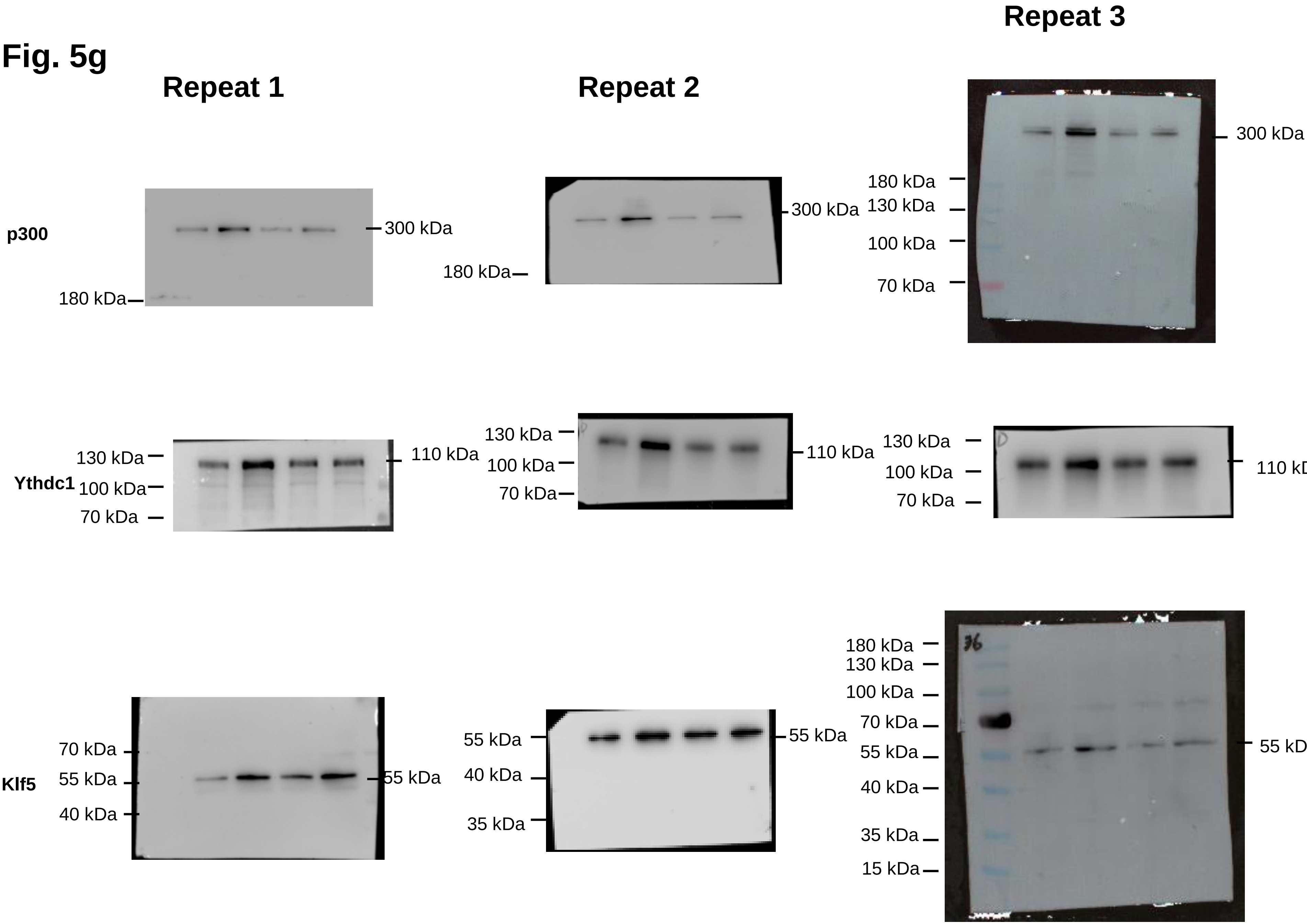
Repeat 1
Repeat 2
Repeat 3
p300
300 kDa
180 kDa
300 kDa
180 kDa
300 kDa
180 kDa
130 kDa
100 kDa
70 kDa
Ythdc1
130 kDa
100 kDa
70 kDa
110 kDa
130 kDa
100 kDa
70 kDa
110 kDa
130 kDa
100 kDa
70 kDa
110 kD
Klf5
70 kDa
55 kDa
40 kDa
55 kDa
55 kDa
40 kDa
35 kDa
55 kDa
180 kDa
130 kDa
100 kDa
70 kDa
55 kDa
40 kDa
35 kDa
15 kDa
55 kD

Fig. 5h

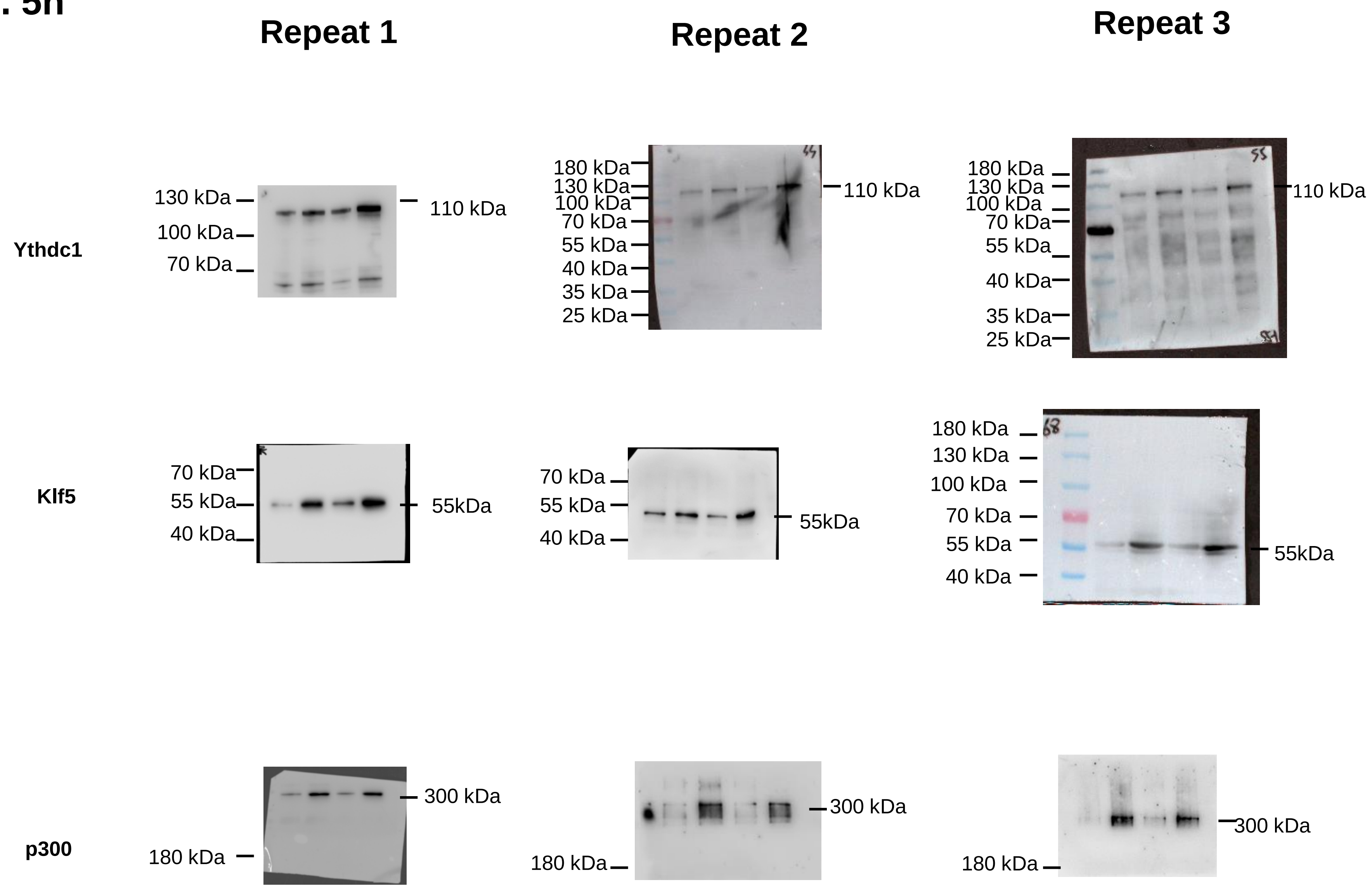

Repeat 1
Repeat 2
Repeat 3
Ythdc1
130 kDa
100 kDa
70 kDa
110 kDa
180 kDa
130 kDa
100 kDa
70 kDa
55 kDa
40 kDa
35 kDa
25 kDa
110 kDa
180 kDa
130 kDa
100 kDa
70 kDa
55 kDa
40 kDa
35 kDa
25 kDa
110 kDa
Klf5
70 kDa
55 kDa
40 kDa
55kDa
70 kDa
55 kDa
40 kDa
55kDa
180 kDa
130 kDa
100 kDa
70 kDa
55 kDa
40 kDa
55kDa
p300
300 kDa
180 kDa
300 kDa
180 kDa
300 kDa
180 kDa

Fig. 5h

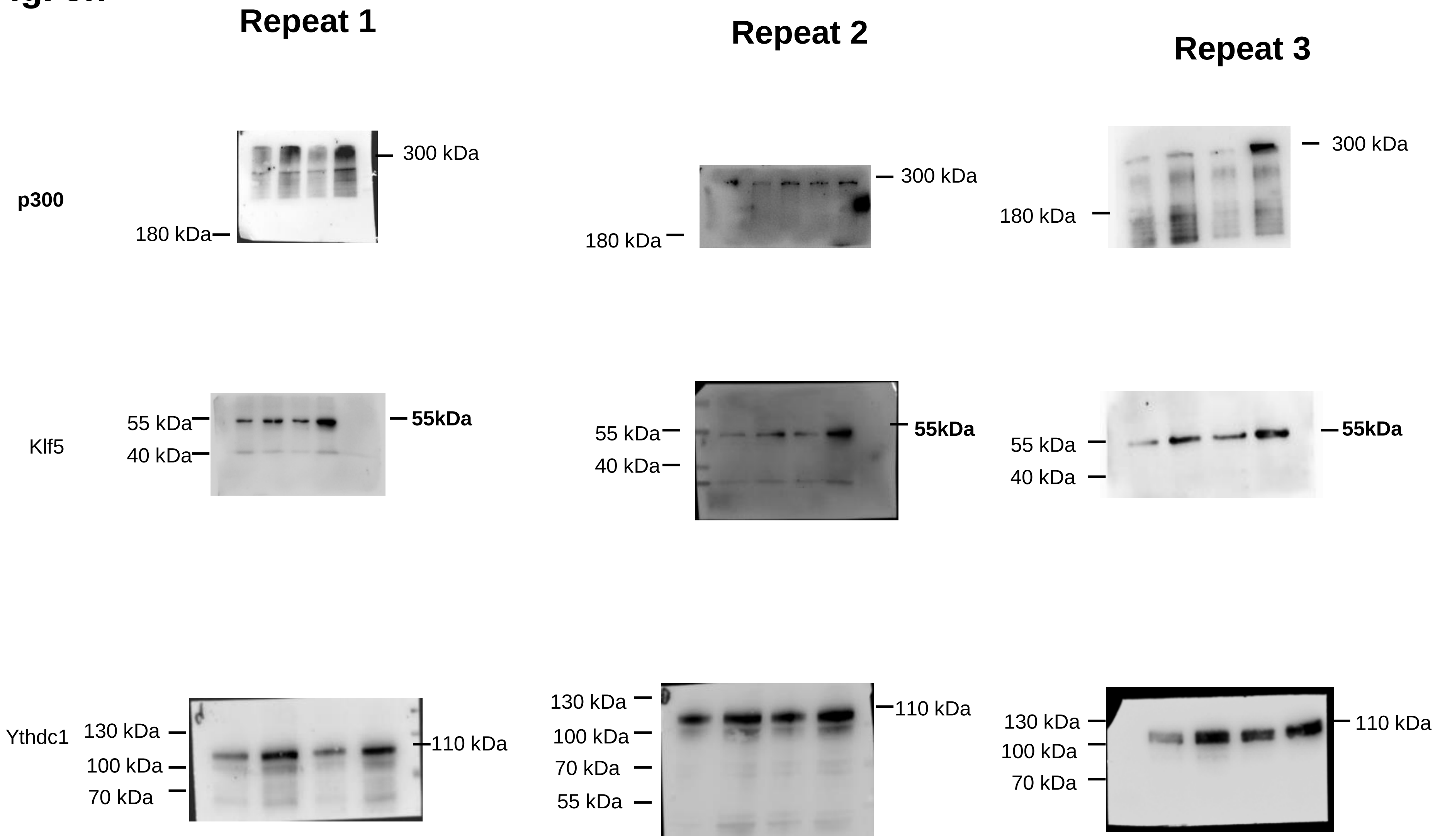
Repeat 1
Repeat 2
Repeat 3
p300
300 kDa
180 kDa
300 kDa
180 kDa
300 kDa
180 kDa
Klf5
55 kDa
40 kDa
55kDa
55 kDa
40 kDa
55kDa
55 kDa
40 kDa
55kDa
Ythdc1
130 kDa
100 kDa
70 kDa
110 kDa
130 kDa
100 kDa
70 kDa
55 kDa
110 kDa
130 kDa
100 kDa
70 kDa
110 kDa

Fig. 5h

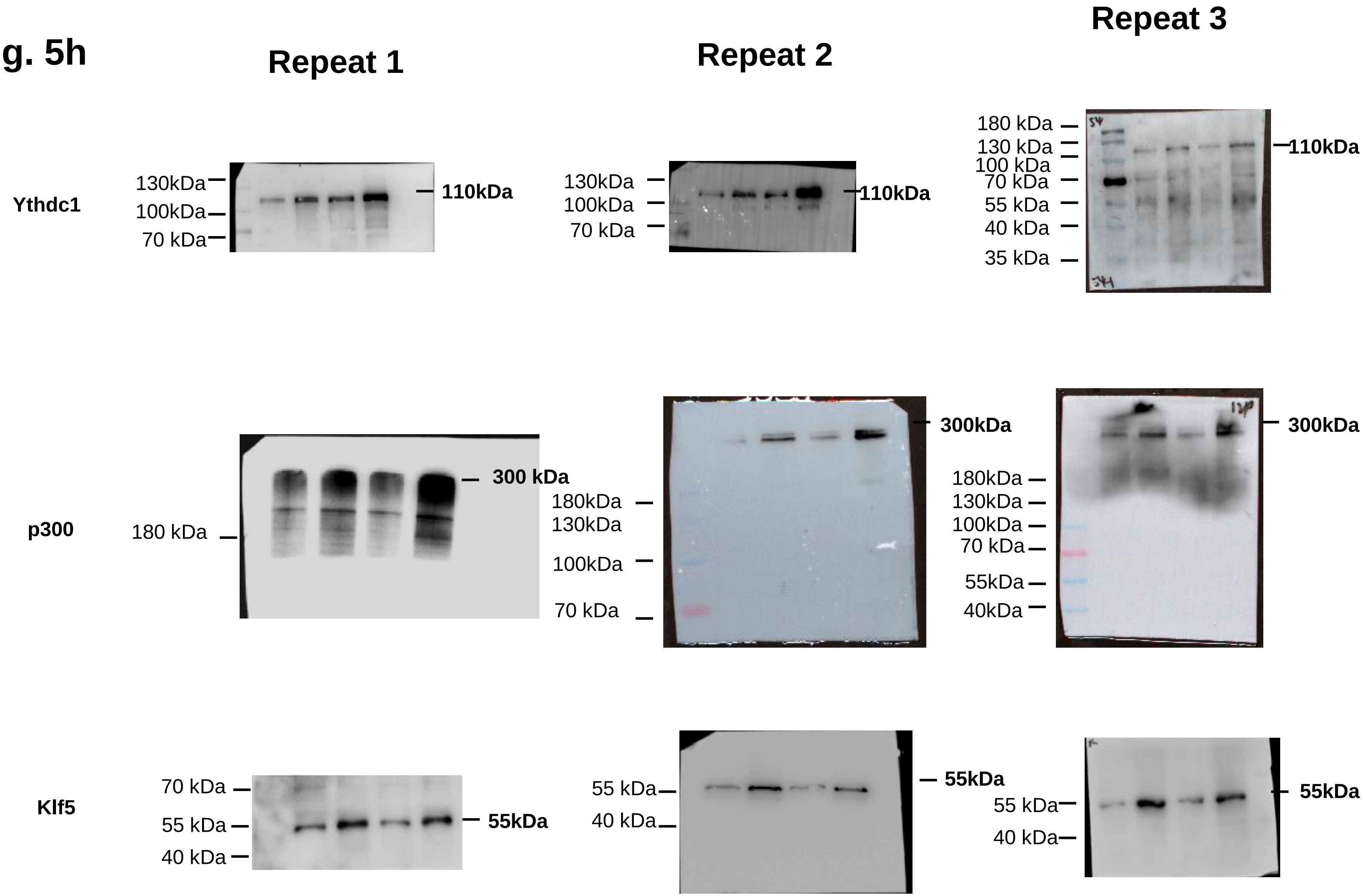
Repeat 1
Repeat 2
Repeat 3
Ythdc1
130kDa
100kDa
70 kDa
110kDa
130kDa
100kDa
70 kDa
110kDa
180 kDa
130 kDa
100 kDa
70 kDa
55 kDa
40 kDa
35 kDa
110kDa
p300
180 kDa
300 kDa
180kDa
130kDa
100kDa
70 kDa
300kDa
180kDa
130kDa
100kDa
70 kDa
55kDa
40kDa
300kDa
Klf5
70 kDa
55 kDa
40 kDa
55kDa
55 kDa
40 kDa
55kDa
55 kDa
40 kDa
55kDa

Fig. 5L

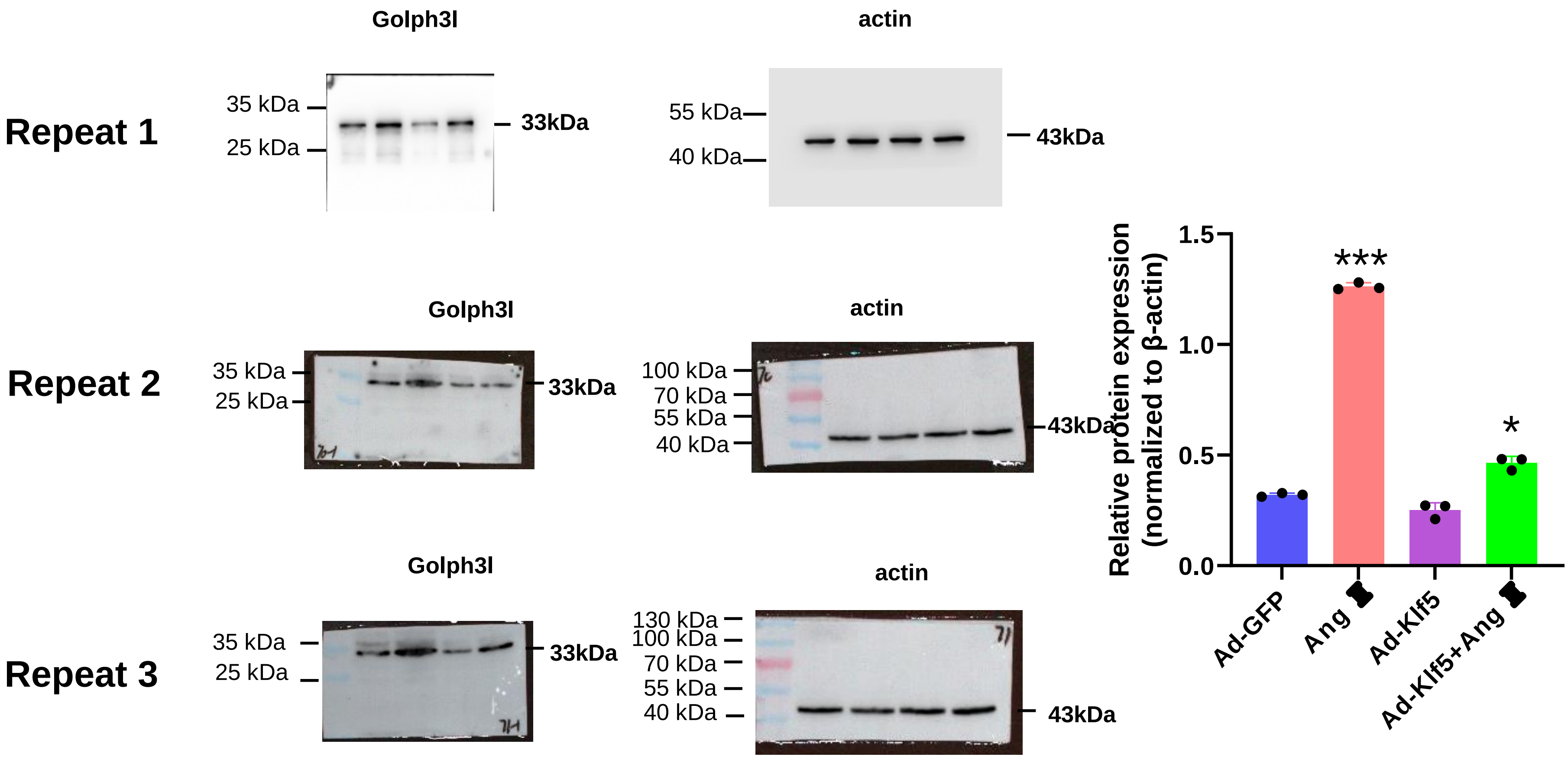
Golph3l
actin
Repeat 1
35 kDa
25 kDa
33kDa
55 kDa
40 kDa
43kDa
Repeat 2
Golph3l
35 kDa
25 kDa
33kDa
actin
100 kDa
70 kDa
55 kDa
40 kDa
43kDa
Repeat 3
Golph3l
35 kDa
25 kDa
33kDa
actin
130 kDa
100 kDa
70 kDa
55 kDa
40 kDa
43kDa
Relative protein expression
(normalized to β-actin)
1.5
1.0
0.5
0.0
***
*
Ad-GFP
Ang
Ad-Klf5
Ad-Klf5+Ang

Fig. 5m

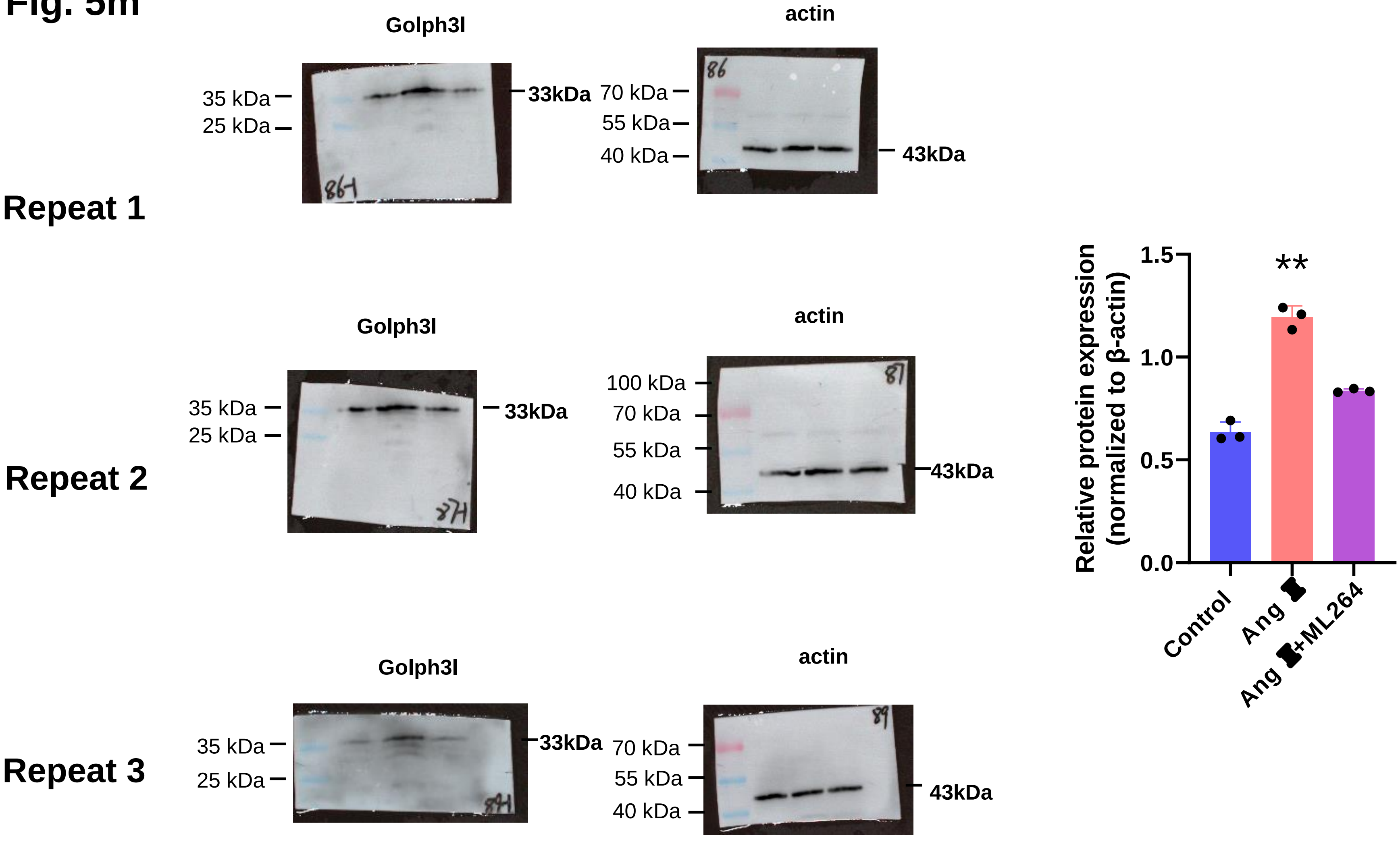

Golph3l
actin
Repeat 1
35 kDa
25 kDa
33kDa
70 kDa
55 kDa
40 kDa
43kDa
Repeat 2
Golph3l
actin
35 kDa
25 kDa
33kDa
100 kDa
70 kDa
55 kDa
40 kDa
43kDa
Repeat 3
Golph3l
actin
35 kDa
25 kDa
33kDa
70 kDa
55 kDa
40 kDa
43kDa
Relative protein expression
(normalized to β-actin)
1.5
1.0
0.5
0.0
**
Control
Ang
+ML264

Fig. 6k

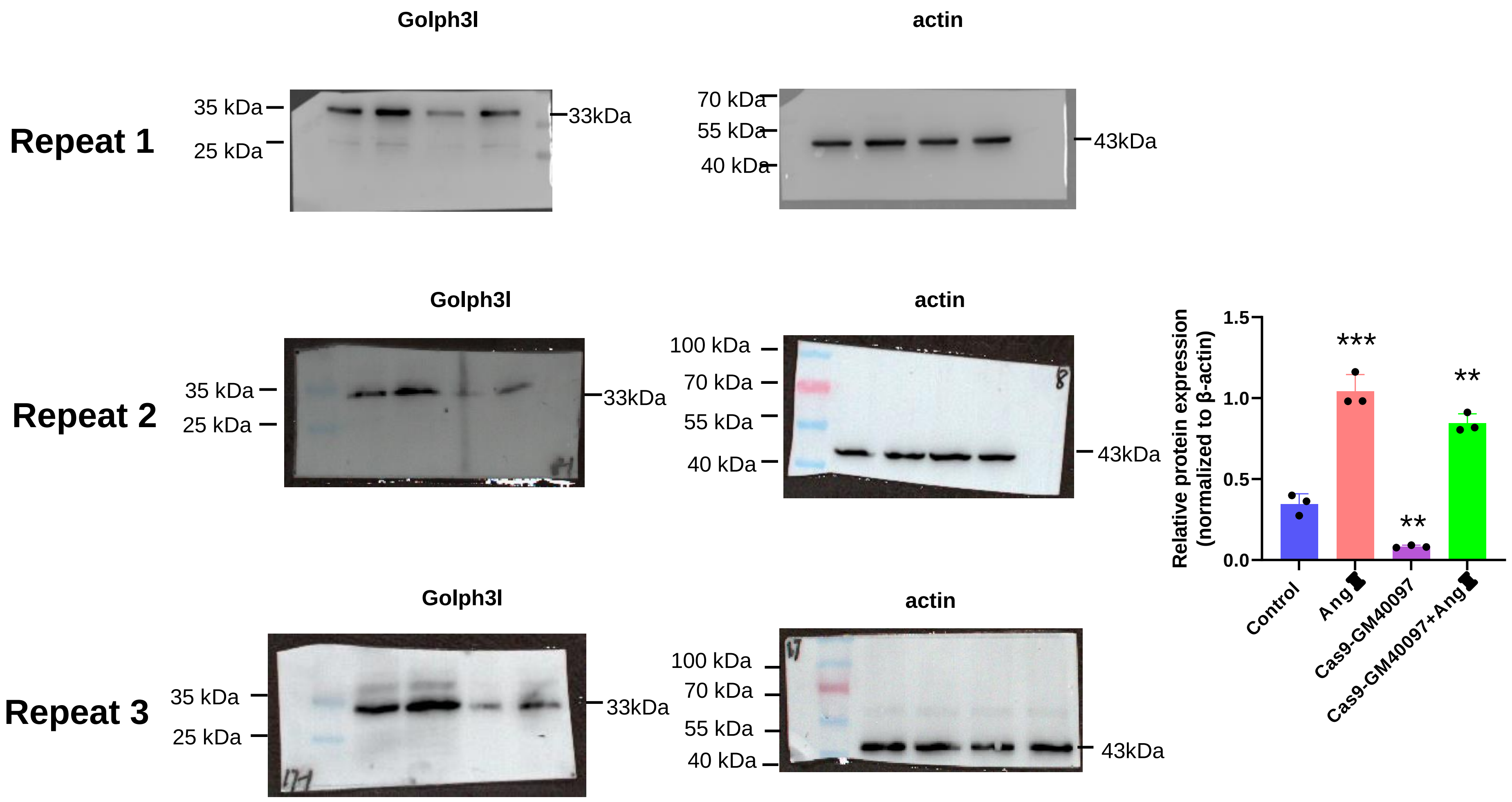

Golph3l
actin
Repeat 1
35 kDa
25 kDa
33kDa
70 kDa
55 kDa
40 kDa
43kDa
Golph3l
actin
Repeat 2
35 kDa
25 kDa
33kDa
100 kDa
70 kDa
55 kDa
40 kDa
43kDa
Golph3l
actin
Repeat 3
35 kDa
25 kDa
33kDa
100 kDa
70 kDa
55 kDa
40 kDa
43kDa
Relative protein expression
(normalized to β-actin)
1.5
1.0
0.5
0.0
***
**
**
Control
Ang
Cas9-GM40097
Cas9-GM40097+Ang

Fig. 7a

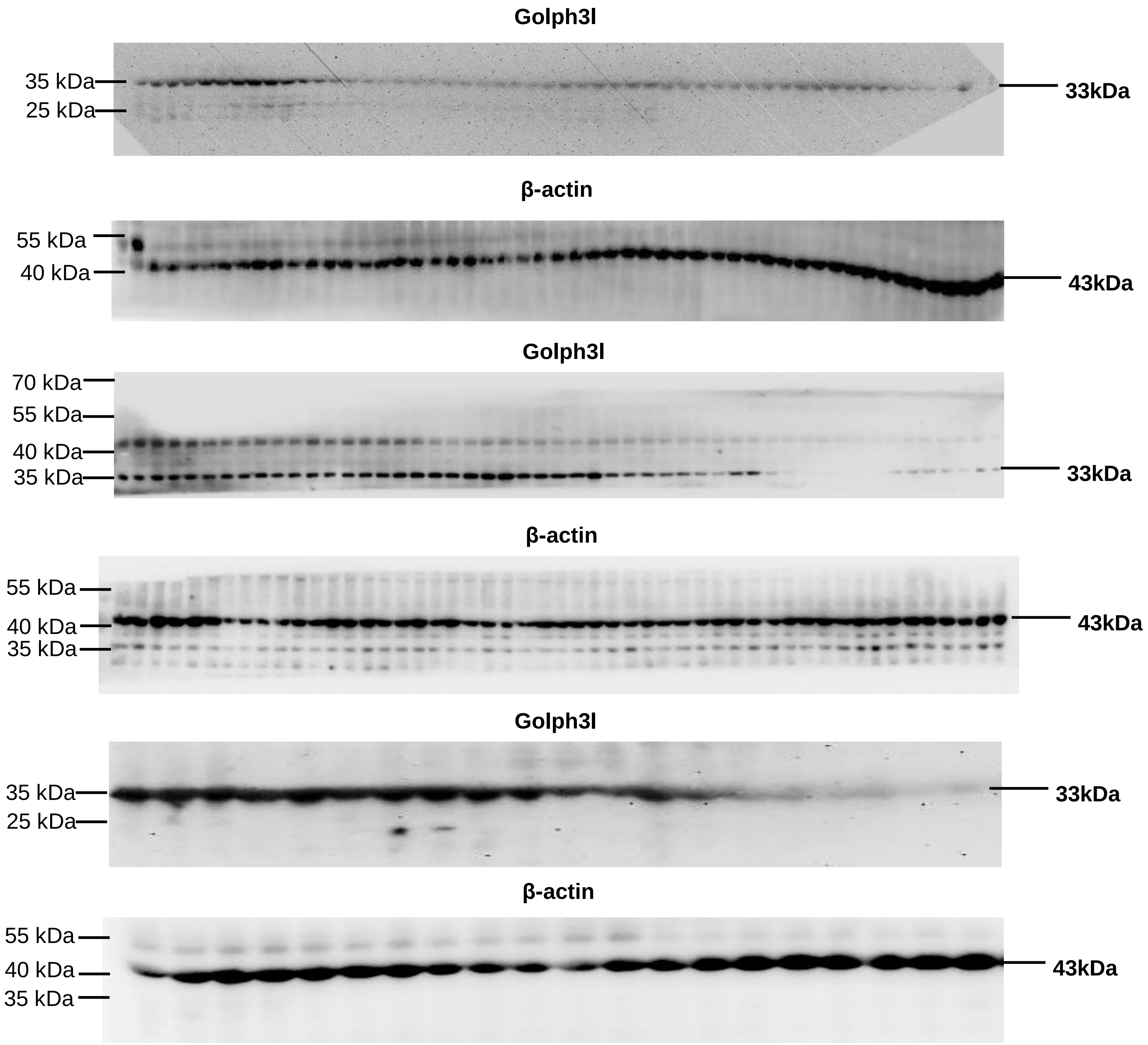
Golph3l
35 kDa
25 kDa
33kDa
β-actin
55 kDa
40 kDa
43kDa
Golph3l
70 kDa
55 kDa
40 kDa
35 kDa
33kDa
β-actin
55 kDa
40 kDa
35 kDa
43kDa
Golph3l
35 kDa
25 kDa
33kDa
β-actin
55 kDa
40 kDa
35 kDa
43kDa

Extended Data fig. 5c

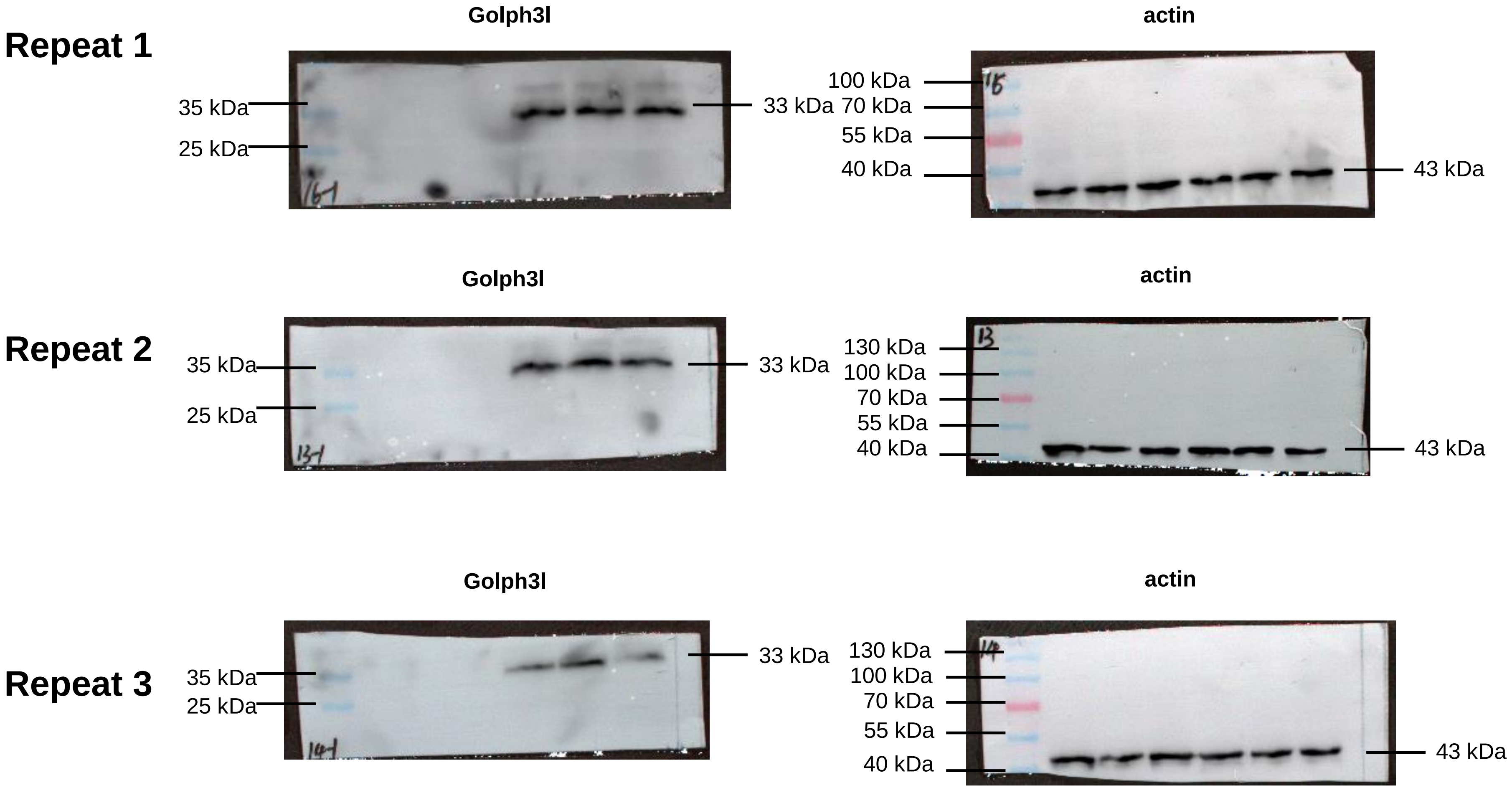

Extended Data fig. 10

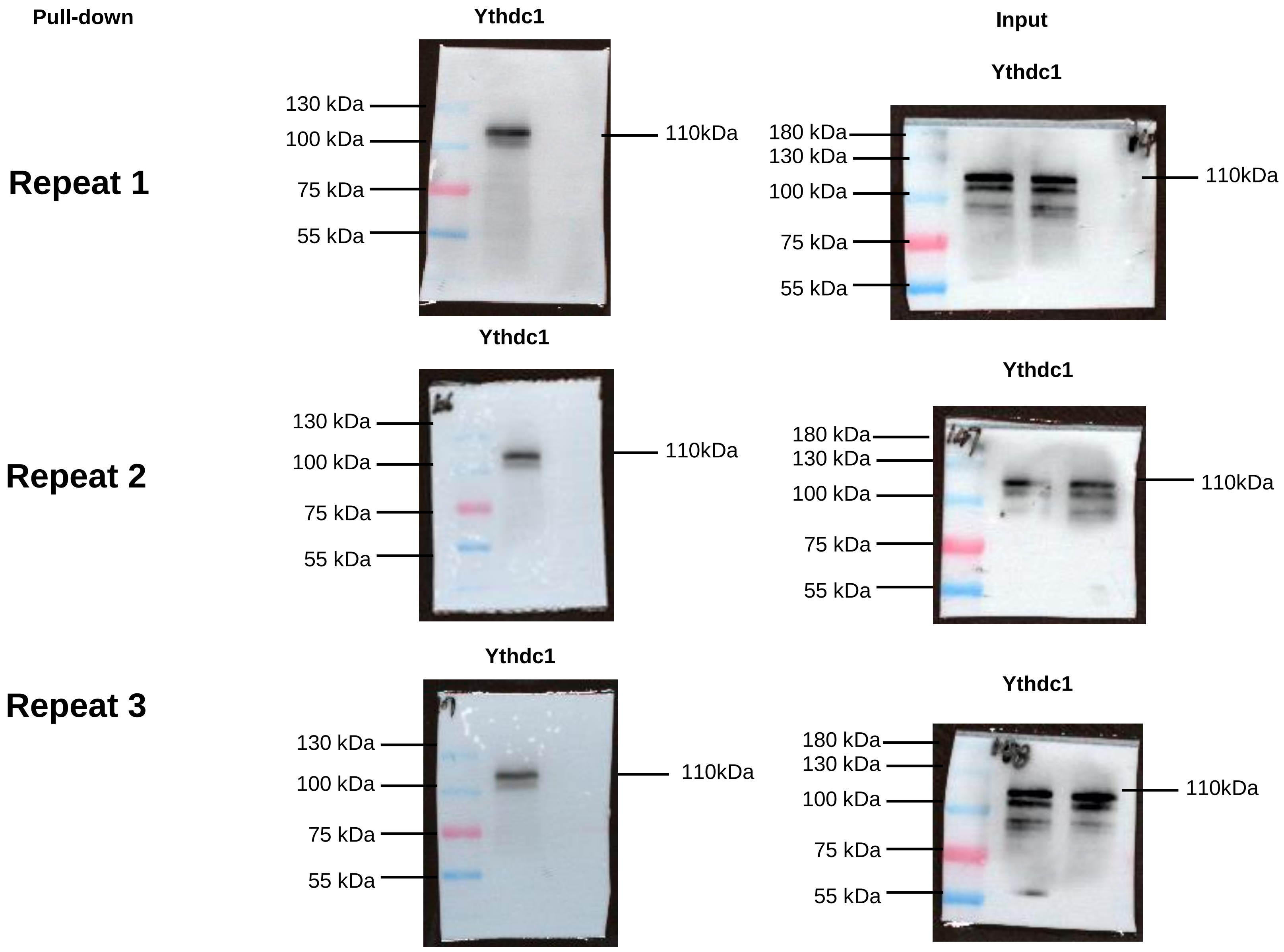

Supplement: Supplementary file 2 — Supporting Information [file ADVS-13-e12116-s002.pdf]
